# Loss of the BRCA1-Interacting Helicase BRIP1 Results in Abnormal Mammary Acinar Morphogenesis

**DOI:** 10.1371/journal.pone.0074013

**Published:** 2013-09-06

**Authors:** Kazuhiro Daino, Tatsuhiko Imaoka, Takamitsu Morioka, Shusuke Tani, Daisuke Iizuka, Mayumi Nishimura, Yoshiya Shimada

**Affiliations:** 1 Radiobiology for Children’s Health Program, Research Center for Radiation Protection, National Institute of Radiological Sciences, Chiba, Japan; 2 Radiation Effect Accumulation and Prevention Project, Fukushima Project Headquarters, National Institute of Radiological Sciences, Chiba, Japan; 3 Department of Experimental Oncology, Division of Genome Biology, Research Institute for Radiation Biology and Medicine, Hiroshima University, Hiroshima, Japan; University of Chicago, United States of America

## Abstract

BRIP1 is a DNA helicase that directly interacts with the C-terminal BRCT repeat of the breast cancer susceptibility protein BRCA1 and plays an important role in BRCA1-dependent DNA repair and DNA damage–induced checkpoint control. Recent studies implicate *BRIP1* as a moderate/low-penetrance breast cancer susceptibility gene. However, the phenotypic effects of BRIP1 dysfunction and its role in breast cancer tumorigenesis remain unclear. To explore the function of BRIP1 in acinar morphogenesis of mammary epithelial cells, we generated BRIP1-knockdown MCF-10A cells by short hairpin RNA (shRNA)-mediated RNA interference and examined its effect in a three-dimensional culture model. Genome-wide gene expression profiling by microarray and quantitative RT-PCR were performed to identify alterations in gene expression in *BRIP1*-knockdown cells compared with control cells. The microarray data were further investigated using the pathway analysis and Gene Set Enrichment Analysis (GSEA) for pathway identification. *BRIP1* knockdown in non-malignant MCF-10A mammary epithelial cells by RNA interference induced neoplastic-like changes such as abnormal cell adhesion, increased cell proliferation, large and irregular-shaped acini, invasive growth, and defective lumen formation. Differentially expressed genes, including *MCAM*, *COL8A1*, *WIPF1*, *RICH2*, *PCSK5*, *GAS1*, *SATB1,* and *ELF3,* in *BRIP1*-knockdown cells compared with control cells were categorized into several functional groups, such as cell adhesion, polarity, growth, signal transduction, and developmental process. Signaling-pathway analyses showed dysregulation of multiple cellular signaling pathways, involving LPA receptor, Myc, Wnt, PI3K, PTEN as well as DNA damage response, in *BRIP1*-knockdown cells. Loss of BRIP1 thus disrupts normal mammary morphogenesis and causes neoplastic-like changes, possibly via dysregulating multiple cellular signaling pathways functioning in the normal development of mammary glands.

## Introduction

Breast cancer is one of the most common epithelial malignancies among woman worldwide. Approximately one in ten women will develop breast cancer during their lifetime in industrialized countries. Although the majority of breast cancers are sporadic in origin, an appreciable fraction is caused by inherited predisposition. Germline mutations in the two major susceptibility genes for breast cancer, namely *BRCA1* and *BRCA2*, confer a 60–85% lifetime risk of breast cancer but account for only about 20% of familial breast cancer cases [1]–[7]. To date, several other breast cancer susceptibility genes, which show low-to-moderate penetrance, have been identified. BRIP1 is one of these genes and truncating mutations confer a 2-fold increase in breast cancer risk [8], [9].

BRIP1 (also known as BACH1 and FANCJ) is a DNA helicase that interacts directly with the breast cancer susceptibility protein BRCA1. BRIP1 plays an important role in controlling BRCA1-dependent DNA repair, DNA damage–induced G2-M checkpoint control, and possibly tumor suppression [10]–[14]. *BRIP1* gene is located on chromosome 17q22, just distal to the *BRCA1* gene located at 17q21, a region that shows frequent loss of heterozygosity in breast cancers [15], [16]. Several loss-of-function mutations in *BRIP1* gene have been reported in breast cancers and also in the cancer-prone disease Fanconi anemia [17]. In addition, some of mutated BRIP1 proteins has been shown to be unstable, with reduced expression [10], [18]. However, the role of BRIP1 in tumorigenesis of mammary epithelial cells remains unclear.

Phenotypic analysis of mice that have a genetic defect in DNA double-strand break repair shows various developmental abnormalities as well as increased tumorigenesis, suggesting a strong link among DNA damage repair, development, and tumorigenesis [19], [20]. For instance, conditional *BRCA1*-knockout mice display incomplete and abnormal mammary gland development [21]. *BRCA1*-knockdown in human mammary epithelial cells abrogates the ability of the cells to form acini in three-dimensional (3D) culture, suggesting a possible role for BRCA1 in mammary cell differentiation [22]. This developmental process is shown to be mediated by the C-terminal BRCT repeat of BRCA1, which interacts with several proteins, including BRIP1. This suggests that BRIP1 plays a role in mammary gland development and tumorigenesis in a BRCA1-dependent or independent manner.

Using 3D basement membrane culture of human non-malignant mammary epithelial MCF-10A cells, we have investigated the roles for BRIP1 in mammary gland development and tumorigenesis. We here show for the first time that *BRIP1* knockdown by RNA interference promotes cell proliferation and disrupts acinar architecture, which may result from dysregulation of genes known to be involved in cell adhesion, polarity, growth, signal transduction, and developmental process, and signaling pathways such as LPA, Myc, Wnt, PI3K, and PTEN. In summary, loss of BRIP1 affects multiple cellular signaling pathways, which are known to be critical for normal development of mammary glands, and its dysfunction disrupts acinar formation.

## Materials and Methods

### Cell Culture and *BRIP1* Knockdown by Lentiviral Transduction of shRNA

MCF-10A cells obtained from American Type Culture Collection, were maintained in a 1∶1 mix of Dulbecco’s modified Eagle’s (DME) and Ham’s F12 media supplemented with 5% equine serum, 10 µg/ml bovine insulin, 20 ng/ml epidermal growth factor, 100 ng/ml cholera toxin, and 0.5 µg/ml hydrocortisone as described [23]. Lentiviral transduction of MCF-10A cells with particles for shRNAs targeting BRIP1 (SHCLNV-NM_032043, MISSION shRNA; Sigma-Aldrich), or scrambled non-target negative control (SHC002V) was performed according to the manufacturer’s protocol. The transduced MCF-10A cells were selected with 1 µg/ml puromycin. The shRNA that showed the highest knockdown efficiency among 5 designed sequences compared with the non-target control was selected.

### Western Blot Analysis

Cellular proteins were extracted with Cell Lysis Buffer (Cell Signaling Technology) containing 1 mM phenylmethylsulfonyl fluoride. Equal amounts of protein were fractionated by 7% SDS-PAGE, transferred to a polyvinylidene difluoride membrane, and reacted with antibodies against BRIP1 (B1310; Sigma-Aldrich), and β-actin (AC-74; Sigma-Aldrich). The protein bands were visualized by enhanced chemiluminescence using ECL Plus Western Blotting Detection System (GE Healthcare).

### 3D Morphogenesis Assays

Cells were cultured on top of a polymerized layer of 100% Matrigel in a 1∶1 mix of DME and Ham’s F12 media supplemented with 2% equine serum, 10 µg/ml insulin, 5 ng/ml epidermal growth factor, 100 ng/ml cholera toxin, 0.5 µg/ml hydrocortisone, and 2% reconstituted basement membrane (Matrigel; BD Biosciences) in a four- or eight-well chamber slide as described [23]. Cells were seeded as single cells at low density of 2.5×10^3^ cells per 0.7 cm^2^ well. At this low initial cell density, individual cells were not in contact under the microscope, and therefore, a possible contribution of cell aggregation to form larger acini structures is excluded. For the acinar structure size calculations, the sizes of at least 150 distinct structures were measured on photomicrographs using ImageJ software (http://rsb.info.nih.gov/ij/). After fixing cells with formalin, slides were mounted with Vectashield mounting medium containing 4′,6-diamidino-2-phenylindole (DAPI; Vector Laboratories) for nuclear staining. Images were acquired with an all-in-one fluorescence microscope (Biozero BZ-8000; Keyence, Osaka, Japan) and analyzed with BZ-Analyzer (Keyence).

### Histology and Immunohistochemistry

Paraffin-embedded/formalin-fixed sections (4 µm thick) of the acini were stained with hematoxylin and eosin or antibody to Ki-67 (clone 7B11; Invitrogen). Antigen–antibody complexes were detected using an anti-mouse secondary antibody and an avidin-biotin complex system (Vector Laboratories) using 3,3′-diaminobenzidine as the chromogen. The percentage of Ki-67-positive cells was determined by counting cells of at least 100 distinct acini in the section.

### DNA Microarray Analysis

Samples were harvested from three different clones of non-target and *BRIP1* shRNA-transduced cells at three time points, 4, 8, and 12 days, after cell seeding in Matrigel. Total RNA was extracted, labeled with Cyanine 3-CTP, and hybridized with human oligonucleotide microarrays (whole-human genome microarray; Agilent Technologies) as described [24]. The raw microarray data were normalized and analyzed using GeneSpring GX 11.5.1 software (Agilent Technologies). Expression data for selected genes were visualized using TIGR MultiExperiment Viewer software (available at http://www.tm4.org/mev.html). The microarray data have been deposited in the Gene Expression Omnibus database (www.ncbi.nlm.nih.gov/geo) under accession number GSE33218. Microarray data were analyzed using GSEA v2.0 [25], [26]. Detailed information is provided in Methods S1.

### Quantitative RT-PCR

Total RNA was isolated from exponentially growing cells in Petri dishes and from 3D cultured cells using the RNeasy kit (Qiagen). First-strand cDNA was synthesized from total RNA as described (Ishida et al., 2010) [24]. The PCR reaction was performed on the Mx3000P real-time PCR system (Stratagene, La Jolla, CA). Primer sequence information is available in Table S5.

### Statistical Analysis

Statistical analysis was carried out with GraphPad StatMate software version 3.0 (ATMS, Tokyo, Japan). Comparison between two groups was done using Student’s *t*-test or non-parametric Mann–Whitney *U*-test. *P*<0.05 was considered to be statistically significant.

### Tumor Growth in Nude Mice

All animal studies were approved by the Institutional Animal Care and Use Committee of the National Institute of Radiological Sciences. 5×10^6^ cells from two different clones of non-target shRNA-transduced cells, three different clones of *BRIP1* shRNA-transduced cells, MCF-7 and MDA-MB-231 human breast cancer cells, as positive controls, were resuspended in 25 µl of phosphate-buffered saline (PBS), mixed with 25 µl of Matrigel, and injected into bilateral abdominal fat pads containing mammary glands of 6-week-old female nude mice (BALB/cAJcl-nu/nu; Clea Japan, Tokyo, Japan; n = 20) under isoflurane anesthesia, and monitored for 10 weeks. Mice were then killed by exsanguination under isoflurane anesthesia and autopsied. Abdominal fat pads were extended on glass slides and fixed in 10% buffered formalin. Hematoxylin-stained whole-mount preparations were observed under a dissection microscope for the occurence of tumors.

## Results

### Generation of BRIP1-knockdown Mammary Epithelial Cells

**Figure 1:**
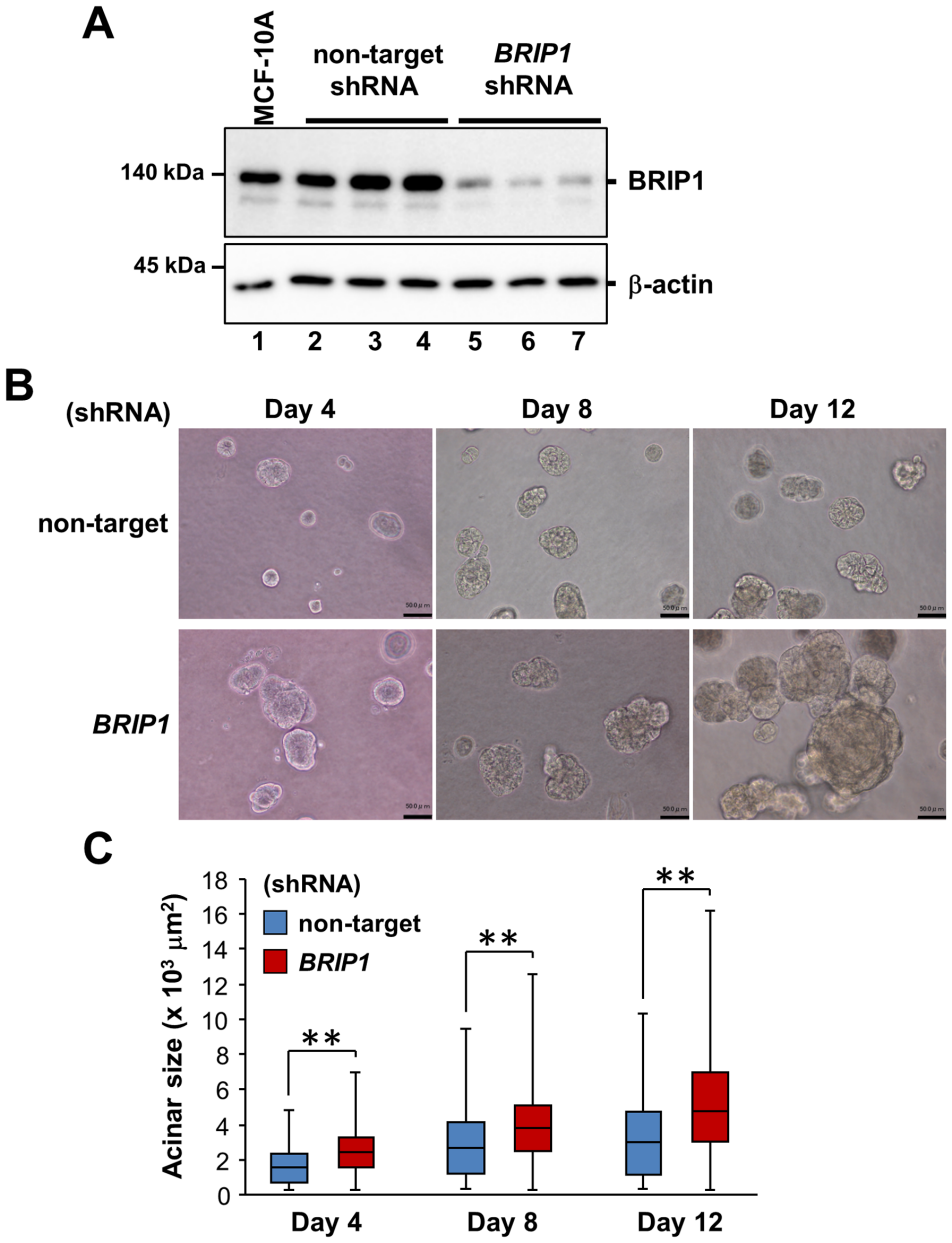
*BRIP1* knockdown in mammary epithelial cells impairs acinus formation. (**A**) *BRIP1* knockdown by lentiviral-mediated delivery of shRNA. Expression of BRIP1 protein in the parental MCF-10A cells (lane 1), three different clones of non-target (lanes 2–4), and *BRIP1* shRNA-transduced (lanes 5–7) cells was analyzed by western blotting with anti-BRIP1. β-actin was used as a loading control. (**B**) Phase-contrast images of cells transduced with non-target shRNA or *BRIP1*-specific shRNA in 3D culture at days 4, 8, and 12. Scale bars, 50 µm. (**C**) Quantification of the size of the acinar structure of the cell lines in 3D culture at days 4, 8, and 12. The median values are indicated with horizontal bars in the boxes. The boxes contain the values between the 25th and 75th percentiles. The whiskers extend to the maximum and minimum values. Comparison between two groups was done using the non-parametric Mann–Whitney *U*-test. **P<0.001.

To explore the function of BRIP1 in acinar morphogenesis of mammary epithelial cells, we generated stable *BRIP1*-knockdown clones by shRNA vector transduction into MCF-10A human non-malignant mammary epithelial cells. *BRIP1* knockdown was confirmed by western blotting (Fig. 1A). In conventional 2D culture, *BRIP1*-knockdown cells formed loose sheets with rounded and loosely attached cells, suggesting weak cellular adherence (Fig. S1).

### Loss of BRIP1 Causes Abnormal Acinar Morphogenesis

**Figure 2:**
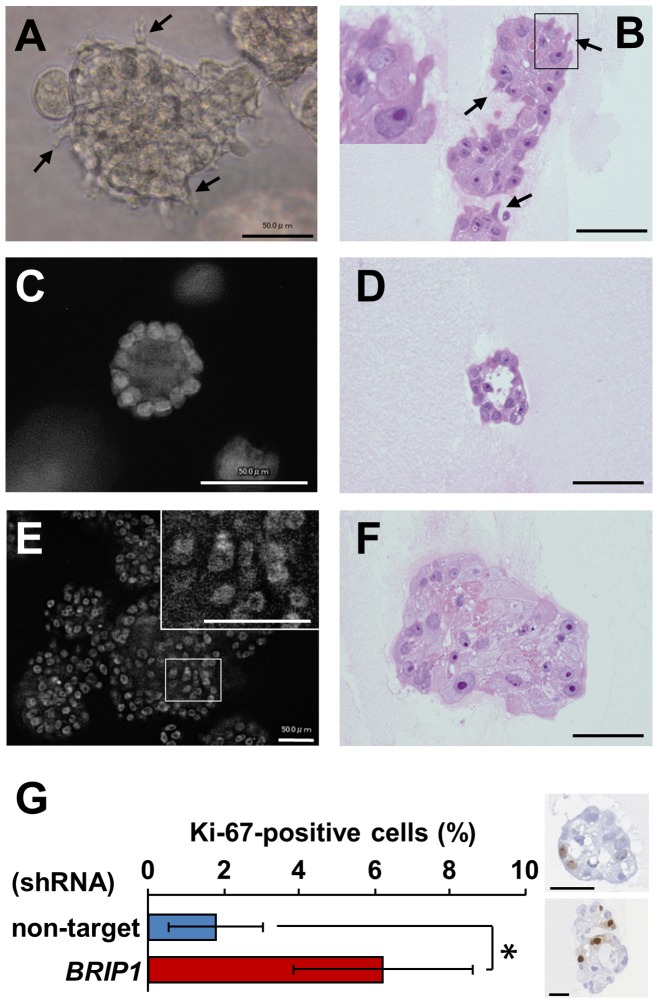
*BRIP1* knockdown induces neoplastic-like changes in mammary acini. Phase-contrast image (**A**) and hematoxylin and eosin-stained section (**B**) of the *BRIP1* shRNA–transduced cells in 3D culture at day 17. Arrows indicate cells with filopodia. The boxed area is enlarged in the inset (**B**). DAPI-stained nuclei (**C, E**) and hematoxylin and eosin-stained section (**D, F**) of cells transduced with the non-target shRNA (**C, D**) or *BRIP1*-specific shRNA (**E, F**) after 20 days in 3D culture. The area indicated by the box is enlarged in the inset (**E**). Scale bars, 50 µm. (**G**) Ki-67 immunostaining of cells transduced with non-target shRNA or *BRIP1*-specific shRNA in 3D culture at day 20. Representative image of Ki-67-stained section of acinar cells transduced with non-target shRNA (*upper right*) or BRIP1-specific shRNA (*lower right*) was shown. Scale bars, 50 µm. Error bars represent the ± SD of the means determined from two independent experiments. Statistical significance was calculated using the Student’s t-test. *P<0.01.

To determine whether *BRIP1* knockdown affects the formation of polarized spherical acini, mammary epithelial cells were grown and monitored in 3D culture. We observed that the acini formed by *BRIP1*-knockdown cells were significantly larger than the control acini after 4 days of culture (Fig. 1B and C). Additionally, *BRIP1*-knockdown cells formed irregular-shaped acinar structures after 8 days of culture (Fig. 1B). In 20 days, the control cells developed into organized acini with hollow lumens (Fig. 2C and D). In contrast, the *BRIP1*-knockdown cells formed large irregular-shaped aggregates with filled lumens and were also characterized by increased cell size, filopodia formation, loss of cellular polarity, nuclear atypia, and nuclear stratification (Fig. 2A, B, E and F). *BRIP1*-knockdown cells also had a 3-fold higher rate of proliferation fraction than the control cells as assessed by Ki-67 immunohistochemistry, even after the control cells formed growth-arrested acini by day 20 (Fig. 2G). These observations suggested that BRIP1 plays a key role in maintaining acinar architecture and that loss of BRIP1 induces a neoplastic-like phenotype in mammary epithelial cells.

### Loss of BRIP1 Induces Dysregulation of Multiple Cellular Signaling Pathways

**Figure 3:**
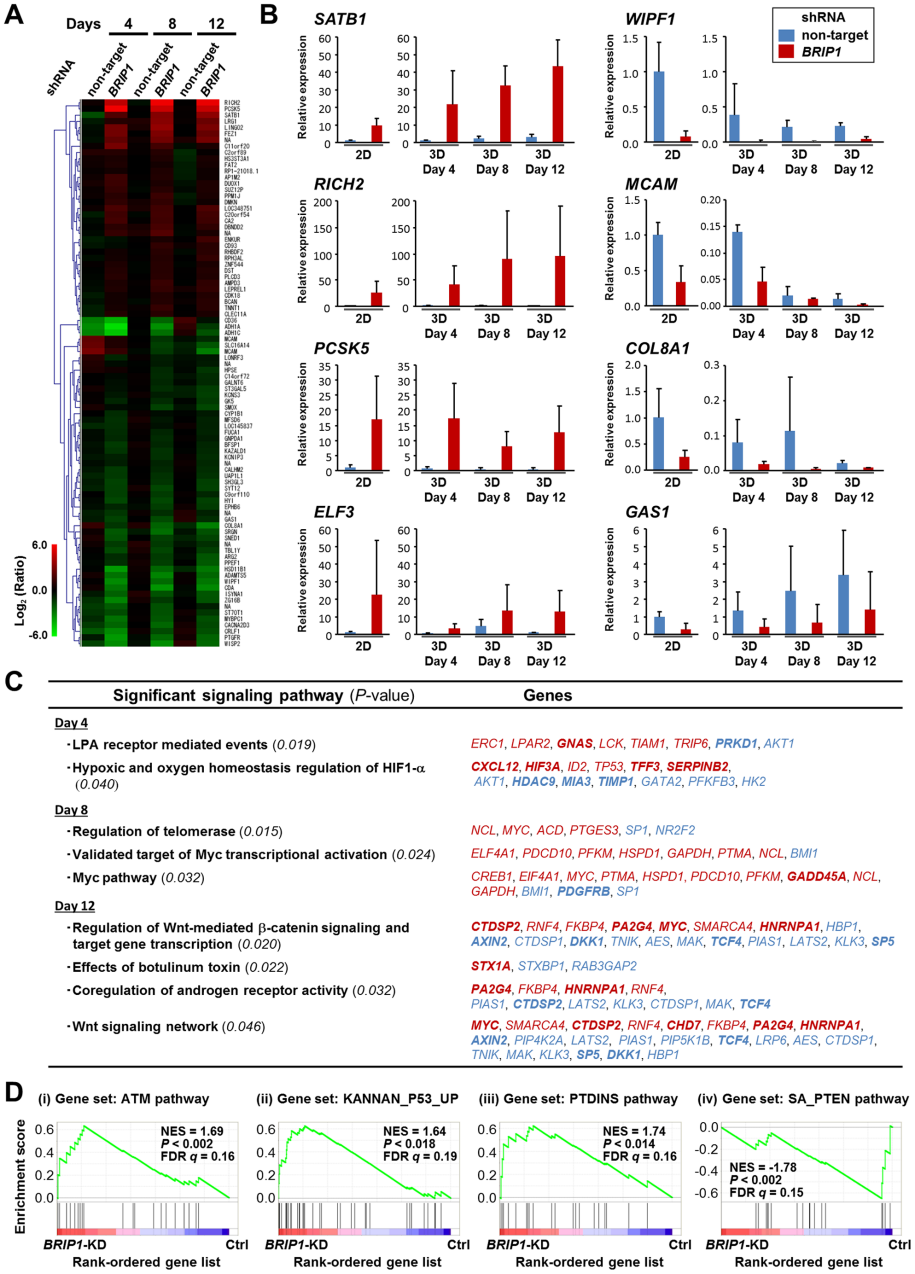
Differentially expressed genes during acinar morphogenesis of *BRIP1*-knockdown mammary epithelial cells. (**A**) Heat map representing the different expression changes of 88 transcripts in *BRIP1* shRNA–transduced cells during acinar morphogenesis compared with non-target shRNA–transduced cells (*P*<0.05; ≥2-fold change). (**B**) Quantitative RT-PCR validation of selected gene expression changes in *BRIP1*-knockdown cells during acinar morphogenesis. The data shown are the mean ± SD of at least two independent experiments with three different clones of shRNA–transduced cells. (**C**) Significantly enriched genes in signaling pathways in the *BRIP1*-knockdown cells during acinar morphogenesis (*P*<0.05). Up- or down-regulated genes are shown in red or blue, respectively. Genes exhibiting a ≥2-fold change in the *BRIP1* shRNA–transduced cells compared with non-target shRNA–transduced cells are indicated in bold. (**D**) GSEA of gene sets up- or down-regulated in 3D-cultured *BRIP1*-knockdown cells compared with control cells. Gene sets from the C2 collection of the Molecular Signatures Database were tested for enrichment in the list of genes ranked by expression change in *BRIP1*-knockdown cells versus control cells. Representative GSEA plots of the enriched gene sets are displayed (*P*<0.05 and FDR *q*<0.25). NES, normalized enrichment score; FDR, false discovery rate; KD, knockdown; Ctrl, control; PTDINS, phosphatidylinositol.

To reveal the genes affected by the loss of BRIP1 during acinar morphogenesis, we performed microarray analysis to clarify the genes differentially expressed during acinar morphogenesis of *BRIP1*-knockdown cells comparing with control cells. Principal component analysis showed that the gene expression profile in the *BRIP1*-knockdown cells during acinar morphogenesis was distinct from that of control cells (Fig. S2A). We identified 379, 288, and 314 transcripts, which were differentially expressed (≥2-fold difference) in 3D culture of *BRIP1*-knockdown cells compared with control cells at 4, 8, and 12 days after culture, respectively (Tables S1–3). Gene Ontology (GO) analysis revealed that subsets of the differentially expressed genes were involved in signal transduction, metabolic processes, developmental process, and cell adhesion, suggesting a link between DNA damage repair and morphogenetic process (Fig. S2B). We also identified 88 transcripts that were consistently up- or down-regulated (fold change ≥2) throughout acinar morphogenesis (Fig. 3A). Table 1 lists selected differentially expressed genes (fold change ≥5). Among them, *SATB1*, which is regarded as a key transcriptional regulator in breast cancer development and metastasis [27], showed high-level expression in *BRIP1*-knockdown cells compared with control cells. We selected several other tumor-associated genes, namely *RICH2*, *PCSK5*, *ELF3*, *WIPF1*, *MCAM*, *COL8A1*, and *GAS1*, based on a literature search, and the changes in gene expression were validated by quantitative real-time reverse transcription-PCR (RT-PCR) (Fig. 3B). Signaling-pathway analysis of the differentially expressed genes revealed enrichment for pathways such as lysophosphatidic acid (LPA) receptor signaling, and oxygen homeostasis, followed by telomerase regulation, Myc signaling and Wnt signaling (Fig. 3C). To further explore the microarray data, gene set enrichment analysis (GSEA) was used to identify groups of functionally related genes for their degree of global up- or down-regulation following *BRIP1* knockdown. This approach identified 33 gene sets that were significantly correlated with *BRIP1* knockdown (Table S4). As shown in Fig. 3D, the gene sets that correlated positively with *BRIP1* knockdown included signaling pathways involved in DNA damage response and cell proliferation, such as ATM, p53 and phosphatidylinositol pathways. In contrast, the negatively correlated gene sets included phosphatase and tensin homologue deleted from chromosome 10 (PTEN) and several cellular metabolic pathways (Fig. 3D and Table S4).

**Table 1:** Dysregulated genes in *BRIP1*-knockdown cells during acinar morphogenesis (ANOVA, *P*<0.05; fold change ≥5.0).

Gene symbol	Name	GenBank ID	Fold change*		
			4d	8d	12d
*PCSK5*	Proprotein convertase subtilisin/kexin type 5	NM_006200	54.3	25.8	22.7
*RICH2*	Rho-type GTPase-activating protein RICH2	NM_014859	17.2	64.5	44.3
*SATB1*	SATB homeobox 1, transcript variant 1	NM_002971	12.9	8.8	9.1
*LINGO2*	Leucine rich repeat and Ig domain containing 2	NM_152570	7.9	7.4	7.4
*FEZ1*	Fasciculation and elongation protein zeta 1 (zygin I), transcript variant 1	NM_005103	7.4	7.6	5.3
*none*	cDNA FLJ61137 complete cds, highly similar to Zinc finger protein 302	AK297551	6.2	4.8	4.2
*C11orf20*	Chromosome 11 open reading frame 20	NM_001039496	6.0	2.6	1.9
*LRG1*	Leucine-rich alpha-2-glycoprotein 1	NM_052972	2.8	5.3	4.9
*CACNA2D3*	Calcium channel alpha2-delta3 subunit	AJ272268	–2.0	–5.3	–4.7
*CRLF1*	Cytokine receptor-like factor 1	NM_004750	–2.2	–4.2	–6.0
*CD36*	CD36 molecule (thrombospondin receptor), transcript variant 2	NM_001001547	–2.4	–5.1	–3.9
*MCAM*	Melanoma cell adhesion molecule	NM_006500	–3.0	–2.2	–5.0
*ADH1C*	Alcohol dehydrogenase 1C (class I), gamma polypeptide	NM_000669	–3.0	–5.7	–6.6
*WISP2*	WNT1 inducible signaling pathway protein 2	NM_003881	–3.2	–8.1	–11.5
*ISYNA1*	Inositol-3-phosphate synthase 1, transcript variant 1	NM_016368	–3.2	–6.7	–3.3
*ADH1A*	Alcohol dehydrogenase 1A (class I), alpha polypeptide	NM_000667	–3.9	–10.6	–5.5
*SRGN*	Serglycin	NM_002727	–5.2	–4.0	–2.8
*COL8A1*	Collagen, type VIII, alpha 1, transcript variant 1	NM_001850	–5.8	–8.6	–4.1
*SNED1*	Sushi, nidogen and EGF-like domains 1	NM_001080437	–6.0	–2.8	–1.7
*COL8A1*	Collagen, type VIII, alpha 1	AL359062	–6.4	–9.7	–7.7
*PTGFR*	Prostaglandin F receptor (FP), transcript variant 2	NM_001039585	–8.3	–4.6	–7.0
*CDA*	Cytidine deaminase	NM_001785	–8.8	–11.0	–8.1
*ADAMTS5*	ADAM metallopeptidase with thrombospondin type 1 motif, 5	NM_007038	–9.2	–10.0	–6.3
*WIPF1*	WAS/WASL interacting protein family, member 1, transcript variant 2	NM_001077269	–10.2	–6.3	–8.0
*HSD11B1*	Hydroxysteroid (11-beta) dehydrogenase 1, transcript variant 2	NM_181755	–11.9	–6.8	–3.6

* A positive fold change indicates increased expression in the *BRIP1*-knockdown cells, and a negative fold change indicates decreased expression in the cells compared with control cells.

### Tumor Growth in Nude Mice

To determine whether the *BRIP1*-knockdown cells become tumorigenic, we injected two different clones of non-target shRNA-transduced cells and three different clones of *BRIP1* shRNA-transduced cells into both sides of the mammary fat pads of 6-week-old female nude mice (5×10^6^ cells on each side at 6 sites for each non-target shRNA-transduced clones and 6 to 8 sites for each *BRIP1* shRNA-transduced clones), and observed for 10 weeks. The *BRIP1*-knockdown cells did not induce tumor formation in nude mice. In this study, we also transplanted MCF-7 and MDA-MB-231 human breast cancer cells into the mammary fat pad as positive controls and these cells did form tumors (data not shown). These results indicate that loss of BRIP1 alone is insufficient to induce tumor formation.

## Discussion

The development of mammary epithelial acini follows a series of morphogenetic processes, including cell migration, cell-cell communication, epithelial polarity establishment, differentiation, and hollow lumen formation by luminal cell death [23]. Here, we revealed that the BRCA1-interacting helicase BRIP1 has important roles in promoting normal acinar morphogenesis and loss of BRIP1 disrupts acinar formation possibly through the dysregulation of multiple celluler signaling pathways.

Our microarray analysis revealed abnormalities in the expression of multiple genes in several signaling pathways in *BRIP1*-knockdown cells (Fig. 3). Alterations in components of the LPA, Myc, Wnt signaling pathways have been implicated in breast cancer development. LPA has a variety of biological actions in cell proliferation, survival, motility, and invasion [28], [29]. Up-regulation of LPA receptors are observed in breast cancer and activation of MAPK and phosphatidylinositol 3-kinase (PI3K)/AKT pathways by LPA are implicated in breast cancer development and progression [30], [31]. Myc is a downstream target of multiple signaling pathways, including MAPK and Wnt, and functions as a transcription factor that regulates numerous genes involved in cell growth, transformation, and angiogenesis. An abnormality in the Myc-regulated pathway is frequently reported in breast cancer [32]. Interestingly, loss of BRCA1 accompanied with Myc over-expression accelerates breast cancer development, especially basal-like breast cancer [33]. Wnt signals play a critical role in regulating several stages of mammary gland growth and differentiation, and dysregulation of Wnt signaling causes breast cancer [34]. In addition, GSEA of *BRIP1*-knockdown cells indicated a significant increase in the expression of genes related to the ATM, p53, and phosphatidylinositol signaling pathways as well as a decrease in the expression of genes involved in PTEN signaling pathway. Activation of the DNA-damage response mediated by the ATM-p53 pathway in *BRIP1*-knockdown cells is consistent with a role of BRIP1 in maintaining genomic stability [35], and activation of the ATM-p53 pathway is also linked to genomic instability in precancerous cells [36]–[38]. On the other hand, reduced expression of the tumor suppressor PTEN correlates with breast cancer progression [39]. Down-regulation of genes associated with PTEN signaling and up-regulation of phosphatidylinositol signaling in *BRIP1*-knockdown cells are consistent with PTEN’s role as a negative regulator of phosphatidylinositol 3-kinase (PI3K) activity. In fact, abnormalities in *PTEN* or *PI3K* expression induce hyperproliferation of epithelial cells and disruption of cell polarity [40]–[42]. Thus, BRIP1 may function as tumor suppressor by affecting these tumor suppressive signaling pathways in the mammary glands.

Notably, transcriptional regulator *SATB1*, Rho-type GTPase-activating protein RICH2 and pro-protein convertase PCSK5 were over-expressed in *BRIP1*-knockdown cells (Table 1 and Fig. 3B). SATB1 is reportedly up-regulated in aggressive breast cancer and is proposed to reprogram the global expression profile of multiple genes involved in cell adhesion, polarity, and growth, as well as the above-mentioned phosphatidylinositol signaling [27], [43]. Ectopic expression of SATB1 is shown to induce tumor-like morphology in 3D culture and lead to tumor formation and lung metastasis in nude mice [44]. RICH2 and PCSK5 play a role in organizing the actin cytoskeleton and cell polarity [45], [46]. The gene for the epithelial-specific Ets transcription factor ELF3 (also known as ESX), involved in mammary gland development [47], was highly expressed during acinar morphogenesis of *BRIP1*-knockdown cells (Table S1 and Fig. 3B). Consistent with this observation, *ELF3* is over-expressed at the early stage of development of ductal carcinoma in situ [48]. We also found down-regulation of genes encoding the adhesion-related molecules COL8A1 and MCAM in *BRIP1*-knockdown cells (Table 1 and Fig. 3B), similar to previous reports [49], [50]. The gene encoding WIPF1, a member of the WASP and WAVE family of proteins, and growth arrest–specific gene *GAS1* were also down-regulated (Table 1, Tables S2, S3 and Fig. 3B). The former is involved in organizing the actin cytoskeleton, cell-cell adhesion, and cell motility [51], and the latter is associated with the higher rate of proliferation (Fig. 1C and Fig. 2G). Taken together, dysregulation of these genes may, at least in part, be associated with the induction of the neoplastic-like changes of *BRIP1*-knockdown cells by the disruption of proper cell adhesion, polarity, growth, and differentiation.

Previously, BRCA1 and SATB1 were shown to be involved in acinar formation of mammary epithelial cells in 3D culture [22], [27], [44]. Analogous to the findings in the *BRCA1*-depleted and *SATB1*-overexpressed cells, abnormalities in acinar formation and proliferation were observed in *BRIP1*-knockdown cells. However, the groups of dysregulated genes in the *BRIP1*-knockdown cells did not coincide with those reported in the *BRCA1*-depleted cells [22], suggesting BRCA1-independent functions of BRIP1 in mammary gland morphogenesis. In support with this, it has been suggested that the majority of BRIP1 exists in a native complex without BRCA1 [18]. On the other hands, it would be interesting as a future study to further investigate the physical interaction between BRIP1 and BRCA1 by comparing side by side the phenotype and dysregulation of genes in single- and double-knockdown cells of both proteins. In contrast, the major downstream signaling of SATB1 involving cell adhesion, polarity, growth and phosphatidylinositol signaling [27] was dysregulated in *BRIP1*-knockdown cells.

In the present study, we further investigated the tumorigenic potential of BRIP1-depleted cells *in vivo*. These cells, which were transplanted into mammary fat pads, did not develop into tumor in nude mice. This result indicates that loss of BRIP1 alone is insufficient to induce tumor formation. This may be related to the fact that BRIP1 is categorized as a moderate risk gene for breast cancer. However, it remains to be established whether the complete inactivation of BRIP1 could induce malignancy. Recently, Ordinario *et al* reported that ATM suppresses SATB1-induced malignant phenotypes of MCF-10A cells [44]. Since our microarray analysis showed an activation of ATM signaling pathway in *BRIP1*-knockdown cells, a part of SATB 1-mediated signaling events for full malignancy may be prevented by ATM in these cells.

In conclusion, the present findings suggest critical roles of BRIP1 helicase in mammary gland development by affecting multiple cellular signaling pathways, and provide a possible mechanism to explain how BRIP1 deficiency contributes to breast tumorigenesis.

## Supporting Information

Figure S1 Morphological change in the *BRIP1*-knockdown mammary epithelial cells. Phase-contrast images of the non-target shRNA–transduced (**A**, **B**) and the *BRIP1* shRNA–transduced (**C**, **D**) cells in conventional 2D culture. Scale bars, 50 µm.
(TIF)

Figure S2 Expression profiling of the *BRIP1*-knockdown mammary epithelial cells. (**A**) Principal component analysis distinguished the *BRIP1* shRNA–transduced cells from the non-target shRNA–transduced cells on the basis of their expression profiles. (**B**) Distribution of the GO biological processes in the dysregulated genes in 3D culture of the *BRIP1*-knockdown mammary epithelial cells. Genes that were significantly up- or down-regulated in cells transduced with the *BRIP1*-specified shRNA compared with those transduced with the non-target shRNA are categorized by their GO biological processes.
(TIF)

Table S1 Up- and down-regulated genes (≥2-fold, P<0.05) in 3D culture of BRIP1-knockdown cells compared with control cells at day 4.
(PDF)

Table S2 Up- and down-regulated genes (≥2-fold, P<0.05) in 3D culture of BRIP1-knockdown cells compared with control cells at day 8.
(PDF)

Table S3 Up- and down-regulated genes (≥2-fold, P<0.05) in 3D culture of BRIP1-knockdown cells compared with control cells at day 12.
(PDF)

Table S4 Gene Set Enrichment Analysis (GSEA) gene sets positively or negatively correlated with 3D cultured BRIP1-knockdown cells compared with control cells.
(PDF)

Table S5 Oligonucleotide sequences used for quantitative RT-PCR.
(PDF)

Methods S1 DNA microarray data analysis.
(DOC)

## References

[1] Easton DF (1999) How many more breast cancer predisposition genes are there? Breast Cancer Res. 1; 14–17. 10.1186/bcr6 PMC138504 11250676

[2] Ponder BA, Antoniou A, Dunning A, Easton DF, Pharoah PD (2005) Polygenic inherited predisposition to breast cancer. Cold Spring Harb Symp Quant Biol 70; 35–41. 10.1101/sqb.2005.70.029 16869736

[3] Szabo CI, King MC (1997) Population genetics of BRCA1 and BRCA2. Am J Hum Genet 60; 1013–1020. PMC1712447 9150148

[4] Antoniou A, Pharoah PD, Narod S, Risch HA, Eyfjord JE, et al. (2003) Average risks of breast and ovarian cancer associated with BRCA1 or BRCA2 mutations detected in case Series unselected for family history: a combined analysis of 22 studies. Am J Hum Genet 72; 1117–1130. 10.1086/375033 PMC1180265 12677558

[5] Serova OM, Mazoyer S, Puget N, Dubois V, Tonin P, et al. (1997) Mutations in BRCA1 and BRCA2 in breast cancer families: are there more breast cancer-susceptibility genes? Am J Hum Genet 60; 486–495. PMC1712515 9042907

[6] Schubert EL, Lee MK, Mefford HC, Argonza RH, Morrow JE, et al. (1997) BRCA2 in American families with four or more cases of breast or ovarian cancer: recurrent and novel mutations, variableexpression, penetrance, and the possibility of families whose cancer is not attributable to BRCA1 or BRCA2. Am J Hum Genet 60; 1031–1040. PMC1712449 9150150

[7] Peto J, Collins N, Barfoot R, Seal S, Warren W, et al. (1999) Prevalence of BRCA1 and BRCA2 gene mutations in patients with early-onset breast cancer. J Natl Cancer Inst 91; 943–949. 10.1093/jnci/91.11.943 10359546

[8] Seal S, Thompson D, Renwick A, Elliott A, Kelly P, et al. (2006) Truncating mutations in the Fanconi anemia J gene BRIP1 are low-penetrance breast cancer susceptibility alleles. Nat Genet 38; 1239–1241. 10.1038/ng1902 17033622

[9] Byrnes GB, Southey MC, Hopper JL (2008) Are the so-called low penetrance breast cancer genes, ATM, BRIP1, PALB2 and CHEK2, high risk for women with strong family histories? Breast Cancer Res 10; 208. 10.1186/bcr2099 PMC2481495 18557994

[10] Cantor SB, Bell DW, Ganesan S, Kass EM, Drapkin R, et al. (2001) BACH1, a novel helicase-like protein, interacts directly with BRCA1 and contributes to its DNA repair function. Cell 105; 149–160. 10.1016/s0092-8674(01)00304-x 11301010

[11] Yu X, Chini CC, He M, Mer G, Chen J (2003) The BRCT domain is a phospho-protein binding domain. Science 302; 639–642. 10.1126/science.1088753 14576433

[12] Cantor S, Drapkin R, Zhang F, Lin Y, Han J, et al. (2004) The BRCA1-associated protein BACH1 is a DNA helicase targeted by clinically relevant inactivating mutations. Proc Natl Acad Sci U S A 101; 2357–2362. 10.1073/pnas.0308717101 PMC356955 14983014

[13] Peng M, Litman R, Jin Z, Fong G, Cantor SB (2006) BACH1 is a DNA repair protein supporting BRCA1 damage response. Oncogene 25; 2245–2253. 10.1038/sj.onc.1209257 16462773

[14] Tu Z, Aird KM, Bitler BG, Nicodemus JP, Beeharry N, et al. (2011) Oncogenic Ras Regulates BRIP1 Expression to Induce Dissociation of BRCA1 from Chromatin, Inhibit DNA Repair, and Promote Senescence. Dev Cell 21; 1077–1091. 10.1016/j.devcel.2011.10.010 PMC3241855 22137763

[15] Phelan CM, Borg A, Cuny M, Crichton DN, Baldersson T, et al. (1998) Consortium study on 1280 breast carcinomas: allelic loss on chromosome 17 targets subregions associated with family history and clinical parameters. Cancer Res 58; 1004–1012. 9500463

[16] Callahan R (1998) Somatic mutations that contribute to breast cancer. Biochem Soc Symp 63; 211–221. 9513725

[17] Cantor SB, Guillemette S (2011) Hereditary breast cancer and the BRCA1-associated FANCJ/BACH1/BRIP1. Future Oncol 7; 253–261. 10.2217/fon.10.191 PMC3109611 21345144

[18] De Nicolo A, Tancredi M, Lombardi G, Flemma CC, Barbuti S, et al. (2008) A novel breast cancer-associated BRIP1 (FANCJ/BACH1) germ-line mutation impairs protein stability and function. Clin Cancer Res 14; 4672–4680. 10.1158/1078-0432.CCR-08-0087 PMC2561321 18628483

[19] Deng CX, Wang RH (2003) Roles of BRCA1 in DNA damage repair: a link between development and cancer. Hum Mol Genet 12; R113–123. 10.1093/hmg/ddg082 12668603

[20] Phillips ER, McKinnon PJ (2007) DNA double-strand break repair and development. Oncogene 26; 7799–7808. 10.1038/sj.onc.1210877 18066093

[21] Xu X, Wagner KU, Larson D, Weaver Z, Li C, et al. (1999) Conditional mutation of Brca1 in mammary epithelial cells results in blunted ductal morphogenesis and tumour formation. Nat Genet 22; 37–43. 10.1038/8743 10319859

[22] Furuta S, Jiang X, Gu B, Cheng E, Chen PL, et al. (2005) Depletion of BRCA1 impairs differentiation but enhances proliferation of mammary epithelial cells. Proc Natl Acad Sci U S A 102; 9176–9181. 10.1073/pnas.0503793102 PMC1166629 15967981

[23] Debnath J, Muthuswamy SK, Brugge JS (2003) Morphogenesis and oncogenesis of MCF-10A mammary epithelial acini grown in three-dimensional basement membrane cultures. Methods 30; 256–268. 10.1016/s1046-2023(03)00032-x 12798140

[24] Ishida Y, Takabatake T, Kakinuma S, Doi K, Yamauchi K, et al. (2010) Genomic and gene expression signatures of radiation in medulloblastomas after low-dose irradiation in Ptch1 heterozygous mice. Carcinogenesis 31; 1694–1701. 10.1093/carcin/bgq145 20616149

[25] Mootha VK, Lindgren CM, Eriksson KF, Subramanian A, Sihag S, et al. (2003) PGC-1alpha-responsive genes involved in oxidative phosphorylation are coordinately downregulated in human diabetes. Nat Genet 34; 267–273. 10.1038/ng1180 12808457

[26] Subramanian A, Tamayo P, Mootha VK, Mukherjee S, Ebert BL, et al. (2005) Gene set enrichment analysis: a knowledge-based approach for interpreting genome-wide expression profiles. Proc Natl Acad Sci U S A 102; 15545–15550. 10.1073/pnas.0506580102 PMC1239896 16199517

[27] Han HJ, Russo J, Kohwi Y, Kohwi-Shigematsu T (2008) SATB1 reprogrammes gene expression to promote breast tumour growth and metastasis. Nature 452; 187–193. 10.1038/nature06781 18337816

[28] Contos JJ, Ishii I, Chun J (2000) Lysophosphatidic acid receptors. Mol Pharmacol 58; 1188–1196. 10.1124/mol.58.6.1188 11093753

[29] Moolenaar WH, van Meeteren LA, Giepmans BN (2004) The ins and outs of lysophosphatidic acid signaling. Bioessays 26; 870–881. 10.1002/bies.20081 15273989

[30] Kitayama J, Shida D, Sako A, Ishikawa M, Hama K, et al. (2004) Over-expression of lysophosphatidic acid receptor-2 in human invasive ductal carcinoma. Breast Cancer Res 6; R640–646. 10.1186/bcr935 PMC1064082 15535846

[31] Liu S, Umezu-Goto M, Murph M, Lu Y, Liu W, et al. (2009) Expression of autotaxin and lysophosphatidic acid receptors increases mammary tumorigenesis, invasion, and metastases. Cancer Cell 15; 539–550. 10.1016/j.ccr.2009.03.027 PMC4157573 19477432

[32] Chen Y, Olopade OI (2008) MYC in breast tumor progression. Expert Rev Anticancer Ther 8; 1689–1698. 10.1586/14737140.8.10.1689 PMC3027840 18925859

[33] Xu J, Chen Y, Olopade OI (2010) MYC and Breast Cancer. Genes Cancer 1; 629–640. 10.1177/1947601910378691 PMC3092228 21779462

[34] Boras-Granic K, Wysolmerski JJ (2008) Wnt signaling in breast organogenesis. Organogenesis 4; 116–122. 10.4161/org.4.2.5858 PMC2634257 19279723

[35] Roy R, Chun J, Powell SN (2012) BRCA1 and BRCA2: different roles in a common pathway of genome protection. Nat Rev Cancer 12; 68–78. 10.1038/nrc3181 PMC4972490 22193408

[36] Gorgoulis VG, Vassiliou LV, Karakaidos P, Zacharatos P, Kotsinas A, Liloglou T, et al. (2005) Activation of the DNA damage checkpoint and genomic instability in human precancerous lesions. Nature 434; 907–913. 10.1038/nature03485 15829965

[37] Bartkova J, Horejsi Z, Koed K, Kramer A, Tort F, et al. (2005) DNA damage response as a candidate anti-cancer barrier in early human tumorigenesis. Nature 434; 864–870. 10.1038/nature03482 15829956

[38] Halazonetis TD, Gorgoulis VG, Bartek J (2008) An oncogene-induced DNA damage model for cancer development. Science 319; 1352–1355. 10.1126/science.1140735 18323444

[39] Cully M, You H, Levine AJ, Mak TW (2006) Beyond PTEN mutations: the PI3K pathway as an integrator of multiple inputs during tumorigenesis. Nat Rev Cancer 6; 184–192. 10.1038/nrc1819 16453012

[40] Fournier MV, Fata JE, Martin KJ, Yaswen P, Bissell MJ (2009) Interaction of E-cadherin and PTEN regulates morphogenesis and growth arrest in human mammary epithelial cells. Cancer Res 69; 4545–4552. 10.1158/0008-5472.CAN-08-1694 PMC2746025 19417140

[41] Liu H, Radisky DC, Wang F, Bissell MJ (2004) Polarity and proliferation are controlled by distinct signaling pathways downstream of PI3-kinase in breast epithelial tumor cells. J Cell Biol 164; 603–612. 10.1083/jcb.200306090 PMC2171976 14769856

[42] Isakoff SJ, Engelman JA, Irie HY, Luo J, Brachmann SM, et al. (2005) Breast cancer-associated PIK3CA mutations are oncogenic in mammary epithelial cells. Cancer Res 65; 10992–11000. 10.1158/0008-5472.CAN-05-2612 16322248

[43] Patani N, Jiang W, Mansel R, Newbold R, Mokbel K (2009) The mRNA expression of SATB1 and SATB2 in human breast cancer. Cancer Cell Int 9; 18. 10.1186/1475-2867-9-18 PMC2731048 19642980

[44] Ordinario E, Han HJ, Furuta S, Heiser LM, Jakkula LR, et al. (2012) ATM Suppresses SATB1-Induced Malignant Progression in Breast Epithelial Cells. PLoS One 7; e51786. 10.1371/journal.pone.0051786 PMC3519734 23251624

[45] Rollason R, Korolchuk V, Hamilton C, Jepson M, Banting G (2009) A CD317/tetherin-RICH2 complex plays a critical role in the organization of the subapical actin cytoskeleton in polarized epithelial cells. J Cell Biol 184; 721–736. 10.1083/jcb.200804154 PMC2686410 19273615

[46] Heng S, Cervero A, Simon C, Stephens AN, Li Y, et al. (2011) Proprotein convertase 5/6 is critical for embryo implantation in women: regulating receptivity by cleaving EBP50, modulating ezrin binding, and membrane-cytoskeletal interactions. Endocrinology 152; 5041–5052. 10.1210/en.2011-1273 21971156

[47] Neve R, Chang CH, Scott GK, Wong A, Friis RR, et al. (1998) The epithelium-specific ets transcription factor ESX is associated with mammary gland development and involution. FASEB J 12; 1541–1550. 10.1096/fasebj.12.14.1541 9806763

[48] Chang CH, Scott GK, Kuo WL, Xiong X, Suzdaltseva Y, et al. (1997) ESX: a structurally unique Ets overexpressed early during human breast tumorigenesis. Oncogene 14; 1617–1622. 10.1038/sj.onc.1200978 9129154

[49] Cechowska-Pasko M, Palka J, Wojtukiewicz MZ (2006) Enhanced prolidase activity and decreased collagen content in breast cancer tissue. Int J Exp Pathol 87; 289–296. 10.1111/j.1365-2613.2006.00486.x PMC2517371 16875494

[50] Shih LM, Hsu MY, Palazzo JP, Herlyn M (1997) The cell-cell adhesion receptor Mel-CAM acts as a tumor suppressor in breast carcinoma. Am J Pathol 151; 745–751. PMC1857834 9284823

[51] Kurisu S, Takenawa T (2009) The WASP and WAVE family proteins. Genome Biol 10; 226. 10.1186/gb-2009-10-6-226 PMC2718491 19589182

