# Supplementary figures and images for: Loss of the BRCA1-Interacting Helicase BRIP1 Results in Abnormal Mammary Acinar Morphogenesis
Source: PLoS One. 2013 Sep 6;8(9):e74013. doi: 10.1371/journal.pone.0074013 (PMC3765252; doi:10.1371/journal.pone.0074013)

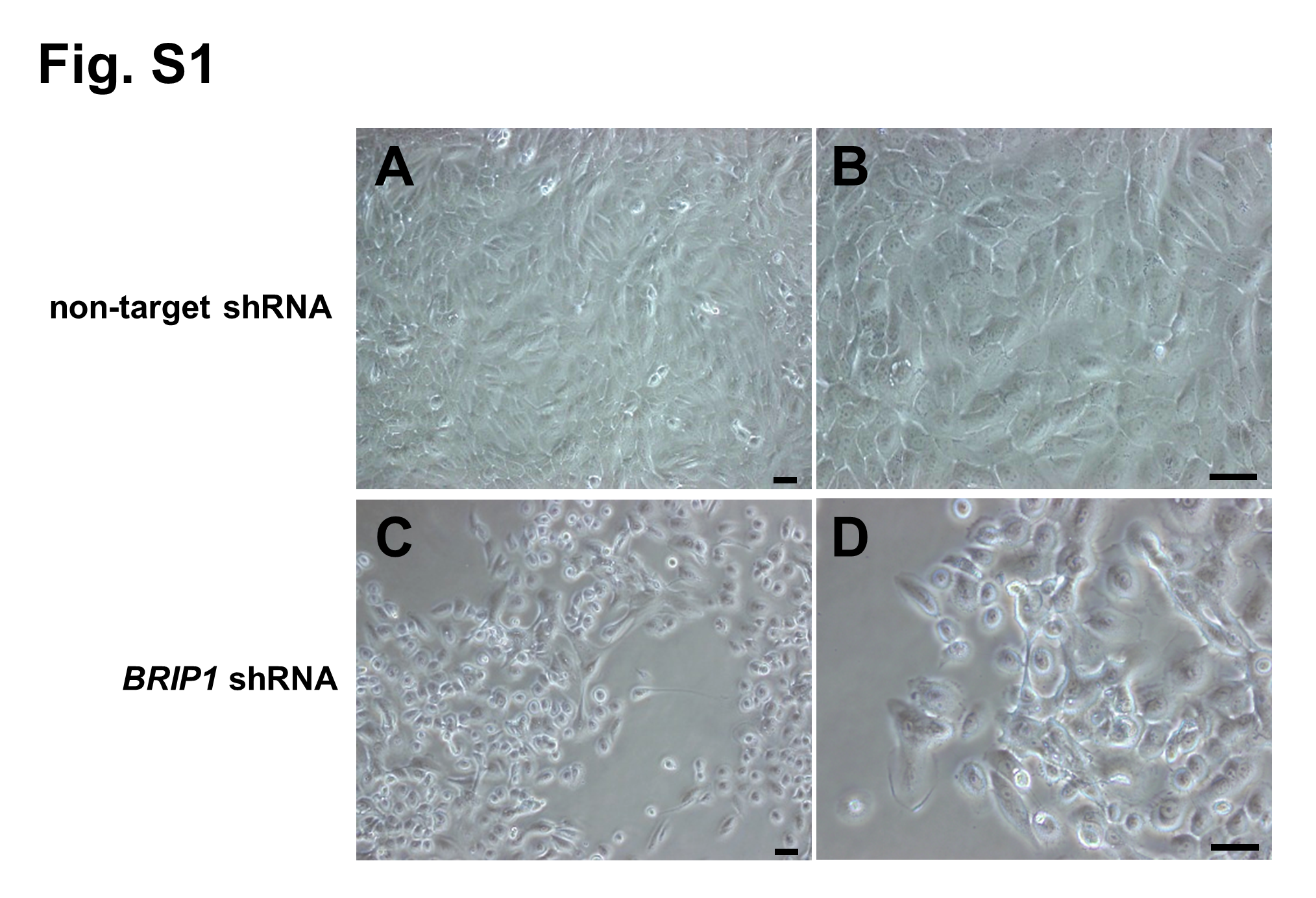

Supplement: Figure S1 — Morphological change in the BRIP1-knockdown mammary epithelial cells. Phase-contrast images of the non-target shRNA–transduced (A, B) and the BRIP1 shRNA–transduced (C, D) cells in conventional 2D culture. Scale bars, 50 µm. (TIF) [file pone.0074013.s001.tif]

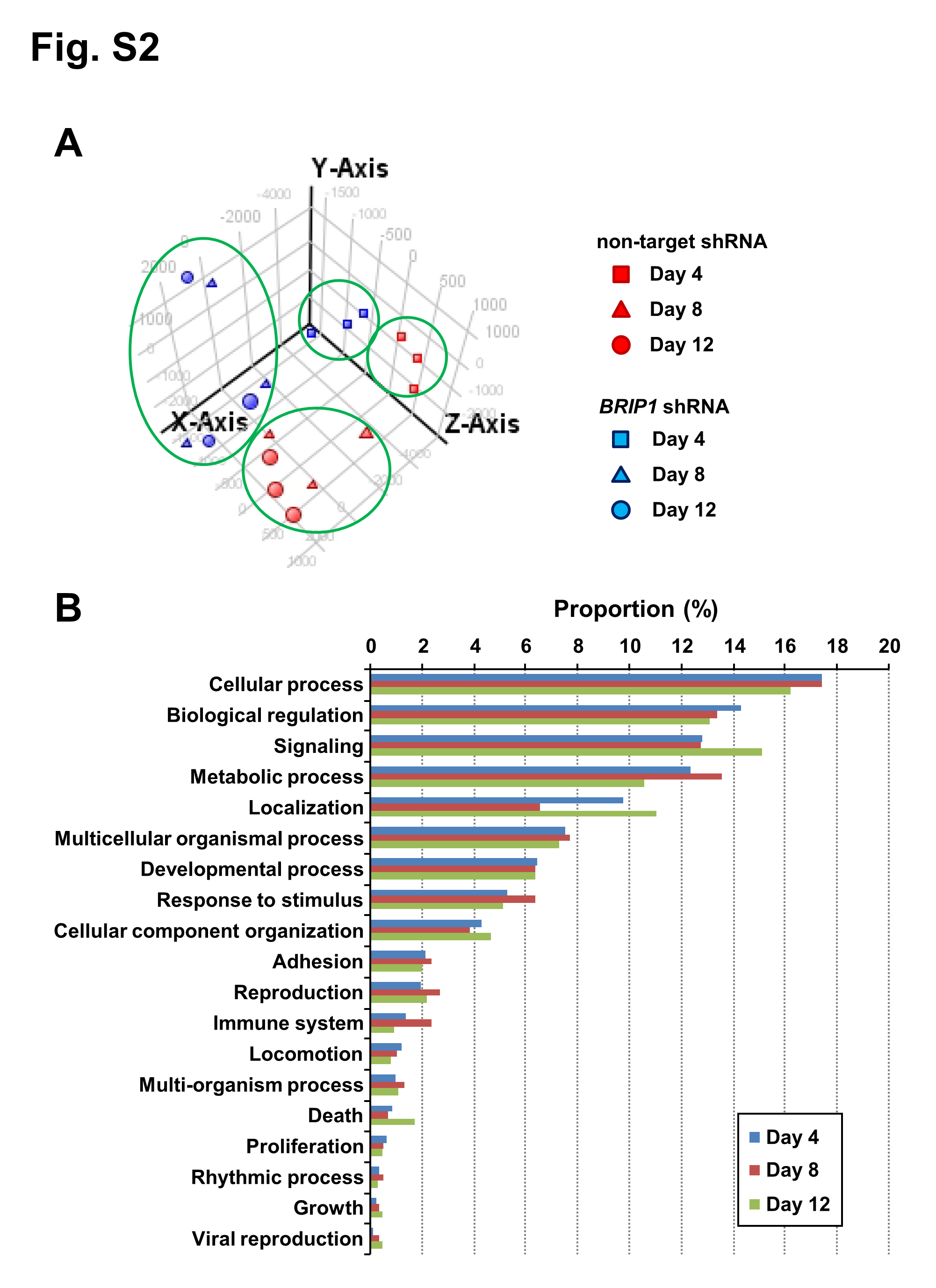

Supplement: Figure S2 — Expression profiling of the BRIP1-knockdown mammary epithelial cells. (A) Principal component analysis distinguished the BRIP1 shRNA–transduced cells from the non-target shRNA–transduced cells on the basis of their expression profiles. (B) Distribution of the GO biological processes in the dysregulated genes in 3D culture of the BRIP1-knockdown mammary epithelial cells. Genes that were significantly up- or down-regulated in cells transduced with the BRIP1-specified shRNA compared with those transduced with the non-target shRNA are categorized by their GO biological processes. (TIF) [file pone.0074013.s002.tif]
