# Supplementary material for: Loss of the BRCA1-Interacting Helicase BRIP1 Results in Abnormal Mammary Acinar Morphogenesis
Source: PLoS One. 2013 Sep 6;8(9):e74013. doi: 10.1371/journal.pone.0074013 (PMC3765252; doi:10.1371/journal.pone.0074013)
Supplement: Table S1 — Up- and down-regulated genes (≥2-fold, P<0.05) in 3D culture of BRIP1-knockdown cells compared with control cells at day 4. (PDF) [file pone.0074013.s003.pdf]

Table S1. Up- and down-regulated genes (≥ 2-fold, $P < 0.05$) in 3D culture of *BRIP1*-knockdown cells compared with control cells at day 4

ProbeName	GeneSymbol	Description	GenbankAccession	Fold change
A_23_P257003	*PCSK5*	Homo sapiens proprotein convertase subtilisin/kexin type 5 (PCSK5), mRNA [NM_006200]	NM_006200	54.35
A_23_P259741	*SATB1*	Homo sapiens SATB homeobox 1 (SATB1), transcript variant 1, mRNA [NM_002971]	NM_002971	12.91
A_23_P101505	*KLK11*	Homo sapiens kallikrein-related peptidase 11 (KLK11), transcript variant 2, mRNA [NM_144947]	NM_144947	8.89
A_23_P29684	*VILL*	Homo sapiens villin-like (VILL), mRNA [NM_015873]	NM_015873	8.30
A_23_P104188	*ELF3*	Homo sapiens E74-like factor 3 (ets domain transcription factor, epithelial-specific) (ELF3), transcript variant 1, mRNA [NM_004433]	NM_004433	8.09
A_23_P202881	*FEZ1*	Homo sapiens fasciculation and elongation protein zeta 1 (zygin I) (FEZ1), transcript variant 1, mRNA	NM_005103	7.42
A_23_P90453	*KRTDAP*	Homo sapiens keratinocyte differentiation-associated protein (KRTDAP), mRNA [NM_207392]	NM_207392	6.93
A_24_P159837		Homo sapiens cDNA FLJ61137 complete cds, highly similar to Zinc finger protein 302. [AK297551]	AK297551	6.18
A_23_P202773	*C11orf20*	Homo sapiens chromosome 11 open reading frame 20 (C11orf20), mRNA [NM_001039496]	NM_001039496	5.97
A_32_P405902	*C21orf15*	Homo sapiens chromosome 21 open reading frame 15 (C21orf15), non-coding RNA [NR_026755]	NR_026755	4.77
A_32_P107617	*SFTPD*	Homo sapiens surfactant protein D (SFTPD), mRNA [NM_003019]	NM_003019	4.66
A_24_P201552	*FEZ1*	Homo sapiens fasciculation and elongation protein zeta 1 (zygin I) (FEZ1), transcript variant 1, mRNA	NM_005103	4.62
A_23_P161297	*OGDHL*	Homo sapiens oxoglutarate dehydrogenase-like (OGDHL), nuclear gene encoding mitochondrial protein, transcript variant 1, mRNA [NM_018245]	NM_018245	4.53
A_23_P57110	*C20orf54*	Homo sapiens chromosome 20 open reading frame 54 (C20orf54), mRNA [NM_033409]	NM_033409	4.43
A_23_P107351	*LOC728392*	Homo sapiens hypothetical protein LOC728392 (LOC728392), mRNA [NM_001162371]	NM_001162371	3.97
A_24_P501544	*YPEL2*	Homo sapiens yippee-like 2 (Drosophila) (YPEL2), mRNA [NM_001005404]	NM_001005404	3.95
A_23_P142815	*ATP6V1B1*	Homo sapiens ATPase, H+ transporting, lysosomal 56/58kDa, V1 subunit B1 (ATP6V1B1), mRNA	NM_001692	3.91
A_23_P383258	*GDA*	Homo sapiens guanine deaminase (GDA), mRNA [NM_004293]	NM_004293	3.86
A_32_P144999		Q2Q5T5_MOUSE (Q2Q5T5) Embryonic stem cell-and germ cell-specific protein ESGP, complete		3.82
A_23_P348208	*SPRR1A*	Homo sapiens small proline-rich protein 1A (SPRR1A), mRNA [NM_005987]	NM_005987	3.78
A_24_P52697	*H19*	Homo sapiens H19, imprinted maternally expressed transcript (non-protein coding) (H19), non-coding RNA	NR_002196	3.76
A_23_P152655	*ICAM2*	Homo sapiens intercellular adhesion molecule 2 (ICAM2), transcript variant 5, mRNA [NM_000873]	NM_000873	3.67
A_23_P8913	*CA2*	Homo sapiens carbonic anhydrase II (CA2), mRNA [NM_000067]	NM_000067	3.65
A_23_P209116	*CYP4F3*	Homo sapiens mRNA for leukotriene B4 omega-hydroxylase, complete cds. [AB002454]	AB002454	3.62
A_23_P374339	*HIF3A*	Homo sapiens hypoxia inducible factor 3, alpha subunit (HIF3A), transcript variant 2, mRNA [NM_022462]	NM_022462	3.56
A_23_P50710	*CYP4F2*	Homo sapiens cytochrome P450, family 4, subfamily F, polypeptide 2 (CYP4F2), mRNA [NM_001082]	NM_001082	3.51
A_23_P71268	*AZGP1*	Homo sapiens alpha-2-glycoprotein 1, zinc-binding (AZGP1), mRNA [NM_001185]	NM_001185	3.37
A_32_P74771				3.25
A_23_P349147	*MUC3B*	Homo sapiens MUC3B mRNA for intestinal mucin, partial cds. [AB038783]	AB038783	3.23
A_32_P186364		Homo sapiens cDNA clone IMAGE:5276765. [BC031314]	BC031314	3.19
A_24_P393571	*GDA*	Homo sapiens guanine deaminase (GDA), mRNA [NM_004293]	NM_004293	3.19
A_32_P191648	*NIN*	AA742651 ny91d02.s1 NCI_CGAP_GCB1 Homo sapiens cDNA clone IMAGE:1285635 3', mRNA sequence	AA742651	3.18
A_23_P36018	*VSIG2*	Homo sapiens V-set and immunoglobulin domain containing 2 (VSIG2), mRNA [NM_014312]	NM_014312	3.13
A_23_P130056	*LYZL6*	Homo sapiens lysozyme-like 6 (LYZL6), mRNA [NM_020426]	NM_020426	3.12
A_23_P71270	*AZGP1*	Homo sapiens alpha-2-glycoprotein 1, zinc-binding (AZGP1), mRNA [NM_001185]	NM_001185	3.11
A_32_P68942	*C21orf81*	Homo sapiens chromosome 21 open reading frame 81 (C21orf81), non-coding RNA [NR_027270]	NR_027270	3.07
A_23_P303833	*SCN4B*	Homo sapiens sodium channel, voltage-gated, type IV, beta (SCN4B), transcript variant 1, mRNA [NM_174934]	NM_174934	3.06
A_23_P33093	*ST6GALNAC5*	Homo sapiens ST6 (alpha-N-acetyl-neuraminyl-2,3-beta-galactosyl-1,3)-N-acetylgalactosaminide alpha-2,6-sialyltransferase 5 (ST6GALNAC5), mRNA [NM_030965]	NM_030965	3.05
A_23_P394986	*CREG2*	Homo sapiens cellular repressor of E1A-stimulated genes 2 (CREG2), mRNA [NM_153836]	NM_153836	3.02
A_24_P307042		Surfactant protein B-binding protein [Source:UniProtKB/TrEMBL;Acc:Q9UD29] [ENST00000422824]	L10404	3.01
A_24_P262127	*RRAD*	Homo sapiens Ras-related associated with diabetes (RRAD), transcript variant 2, mRNA [NM_004165]	NM_004165	2.99
A_23_P395001	*SLC2A12*	Homo sapiens solute carrier family 2 (facilitated glucose transporter), member 12 (SLC2A12), mRNA	NM_145176	2.99
A_23_P331748	*CD33*	Homo sapiens CD33 molecule (CD33), transcript variant 1, mRNA [NM_001772]	NM_001772	2.98
A_24_P118962	*NHEDC1*	Homo sapiens Na+/H+ exchanger domain containing 1 (NHEDC1), transcript variant 1, mRNA [NM_139173]	NM_139173	2.95
A_23_P153489	*CLEC11A*	Homo sapiens C-type lectin domain family 11, member A (CLEC11A), mRNA [NM_002975]	NM_002975	2.94
A_23_P115022	*TMEM125*	Homo sapiens transmembrane protein 125 (TMEM125), mRNA [NM_144626]	NM_144626	2.93
A_24_P849467	*ZNF585A*	Homo sapiens cDNA FLJ41302 fis, clone BRAMY2042122. [AK123296]	AK123296	2.90
A_23_P145786	*MLXIPL*	Homo sapiens MLX interacting protein-like (MLXIPL), transcript variant 1, mRNA [NM_032951]	NM_032951	2.87
A_24_P686247	*OCLN*	occludin [Source:HGNC Symbol;Acc:8104] [ENST00000355237]	DQ786238	2.84
A_24_P53595	*GNAS*	Homo sapiens GNAS complex locus (GNAS), transcript variant 4, mRNA [NM_016592]	NM_016592	2.84
A_23_P154818	*C21orf15*	Homo sapiens mRNA; cDNA DKFZp686M2227 (from clone DKFZp686M2227). [BX648100]	BX648100	2.84
A_24_P367439	*LOC440839*	Homo sapiens tigger transposable element derived 1 pseudogene (LOC440839), non-coding RNA	NR_029399	2.84
A_32_P162709		full-length cDNA clone CS0DC002YA18 of Neuroblastoma Cot 25-normalized of Homo sapiens (human).	CR624517	2.83
A_24_P895836		Homo sapiens, clone IMAGE:4720764, mRNA. [BC016022]	BC016022	2.80
A_32_P71456				2.76
A_23_P50638	*LRG1*	Homo sapiens leucine-rich alpha-2-glycoprotein 1 (LRG1), mRNA [NM_052972]	NM_052972	2.76
A_24_P204011	*FOXR1*	Homo sapiens forkhead box R1 (FOXR1), mRNA [NM_181721]	NM_181721	2.74
A_32_P168326	*ANKRD20A2*	Homo sapiens ankyrin repeat domain 20 family, member A2 (ANKRD20A2), mRNA [NM_001012421]	NM_001012421	2.74
A_23_P88849	*RRAD*	Homo sapiens Ras-related associated with diabetes (RRAD), transcript variant 2, mRNA [NM_004165]	NM_004165	2.74
A_24_P6125	*KCNJ4*	Homo sapiens potassium inwardly-rectifying channel, subfamily J, member 4 (KCNJ4), transcript variant 1, mRNA [NM_152868]	NM_152868	2.74
A_23_P157695		AA609749 af17d08.s1 Soares_testis_NHT Homo sapiens cDNA clone IMAGE:1031919 3' similar to TR:G804804 G804804 HYPOTHETICAL 4.7 KD PROTEIN. ;, mRNA sequence [AA609749]	AA609749	2.73
A_24_P862886	*DRD5*	Homo sapiens dopamine receptor D5 (DRD5), mRNA [NM_000798]	NM_000798	2.73
A_32_P81514		full-length cDNA clone CS0DI036YE11 of Placenta Cot 25-normalized of Homo sapiens (human). [CR593784]	CR593784	2.72
A_24_P247587	*LOC348751*	Homo sapiens hypothetical protein LOC348751, mRNA (cDNA clone IMAGE:5311172). [BC039445]	BC039445	2.71
A_32_P172882	*FLJ41350*	Homo sapiens hypothetical protein LOC399806 (FLJ41350), non-coding RNA [NR_029380]	NR_029380	2.70
A_23_P213336	*FGF1*	Homo sapiens fibroblast growth factor 1 (acidic) (FGF1), transcript variant 1, mRNA [NM_000800]	NM_000800	2.70
A_24_P315854		Homo sapiens clone 20270+t7 immunoglobulin mu heavy chain mRNA, partial cds. [AY671312]	AY671312	2.69
A_23_P139986	*OXGR1*	Homo sapiens oxoglutarate (alpha-ketoglutarate) receptor 1 (OXGR1), mRNA [NM_080818]	NM_080818	2.69
A_32_P132300		Synthetic construct Homo sapiens gateway clone IMAGE:100021839 3' read MS4A6A mRNA. [CU692741]	CU692741	2.69
A_23_P79302	*LYPD6B*	Homo sapiens LY6/PLAUR domain containing 6B (LYPD6B), mRNA [NM_177964]	NM_177964	2.67
A_24_P764690	*ZNF720*	full-length cDNA clone CS0DN002YO09 of Adult brain of Homo sapiens (human). [CR611323]	CR611323	2.67
A_23_P373017	*CCL3*	Homo sapiens mRNA for pLD78 peptide, complete cds. [D00044]	D00044	2.66
A_24_P332043	*SPAG17*	Homo sapiens sperm associated antigen 17 (SPAG17), mRNA [NM_206996]	NM_206996	2.65
A_24_P42140				2.65
A_24_P930418	*RBPMS*	Homo sapiens cDNA, FLJ17575. [AK310533]	AK310533	2.65
A_24_P374943	*CXADR*	Homo sapiens coxsackie virus and adenovirus receptor (CXADR), mRNA [NM_001338]	NM_001338	2.63
A_23_P99625	*FITM1*	Homo sapiens fat storage-inducing transmembrane protein 1 (FITM1), mRNA [NM_203402]	NM_203402	2.62
A_23_P53370	*RND1*	Homo sapiens Rho family GTPase 1 (RND1), mRNA [NM_014470]	NM_014470	2.62
A_23_P2674	*KRT4*	Homo sapiens keratin 4 (KRT4), mRNA [NM_002272]	NM_002272	2.61
A_23_P362798	*SLC30A8*	Homo sapiens solute carrier family 30 (zinc transporter), member 8 (SLC30A8), transcript variant 1, mRNA	NM_173851	2.59
A_24_P144666				2.58
A_24_P360722	*DIP2C*	Homo sapiens DIP2 disco-interacting protein 2 homolog C (Drosophila) (DIP2C), mRNA [NM_014974]	NM_014974	2.58
A_23_P121665	*SORCS2*	Homo sapiens sortilin-related VPS10 domain containing receptor 2 (SORCS2), mRNA [NM_020777]	NM_020777	2.58
A_32_P921149	*LOC100129476*	Homo sapiens cDNA FLJ25733 fis, clone TST05652. [AK098599]	AK098599	2.57
A_32_P194887				2.57
A_24_P942296	*CNOT2*	Homo sapiens mRNA; cDNA DKFZp434M0572 (from clone DKFZp434M0572); partial cds. [AL137674]	AL137674	2.57
A_24_P296274	*NCRNA00052*	Homo sapiens non-protein coding RNA 52 (NCRNA00052), non-coding RNA [NR_026869]	NR_026869	2.56
A_23_P89334	*MYH13*	Homo sapiens myosin, heavy chain 13, skeletal muscle (MYH13), mRNA [NM_003802]	NM_003802	2.56
A_32_P217853		602039925F2 NCI_CGAP_Brn67 Homo sapiens cDNA clone IMAGE:4177446 5', mRNA sequence [BF527031]	BF527031	2.55
A_32_P213615		Homo sapiens cDNA FLJ39459 fis, clone PROST2011439. [AK096778]	AK096778	2.55
A_32_P379467	*ISLR2*	Homo sapiens immunoglobulin superfamily containing leucine-rich repeat 2 (ISLR2), transcript variant 2, mRNA [NM_020851]	NM_020851	2.55
A_24_P37873	*ESM1*	Homo sapiens endothelial cell-specific molecule 1 (ESM1), transcript variant 1, mRNA [NM_007036]	NM_007036	2.54
A_23_P131060	*CYP4F8*	Homo sapiens cytochrome P450, family 4, subfamily F, polypeptide 8 (CYP4F8), mRNA [NM_007253]	NM_007253	2.54

A_24_P160586	*SERINC4*	Homo sapiens cDNA FLJ40363 fis, clone TESTI2034718, weakly similar to Mus musculus membrane protein TMS-2 mRNA. [AK097682]	AK097682	2.53
A_23_P312874	*SPATS1*	Homo sapiens spermatogenesis associated, serine-rich 1 (SPATS1), mRNA [NM_145026]	NM_145026	2.52
A_23_P389500	*REG1B*	Homo sapiens regenerating islet-derived 1 beta (REG1B), mRNA [NM_006507]	NM_006507	2.52
A_24_P167654	*SLC8A3*	Homo sapiens solute carrier family 8 (sodium/calcium exchanger), member 3 (SLC8A3), transcript variant c, mRNA [NM_183002]	NM_183002	2.51
A_23_P59960	*CRISPLD1*	Homo sapiens cysteine-rich secretory protein LCCL domain containing 1 (CRISPLD1), mRNA [NM_031461]	NM_031461	2.51
A_32_P138652		Homo sapiens IQCF protein-like mRNA, complete sequence. [GQ870251]	GQ870251	2.51
A_24_P531312		AA515157 ng68d08.s1 NCI_CGAP_Lip2 Homo sapiens cDNA clone IMAGE:939951 similar to contains Alu repetitive element;, mRNA sequence [AA515157]	AA515157	2.49
A_23_P8497	*GHRHR*	Homo sapiens growth hormone releasing hormone receptor (GHRHR), transcript variant 1, mRNA	NM_000823	2.49
A_24_P932646	*UNQ5830*	Homo sapiens clone DNA149986 AILT5830 (UNQ5830) mRNA, complete cds. [AY358123]	AY358123	2.48
A_23_P19529	*MLN*	Homo sapiens motilin (MLN), transcript variant 2, mRNA [NM_001040109]	NM_001040109	2.47
A_23_P15692	*GPR172B*	Homo sapiens G protein-coupled receptor 172B (GPR172B), transcript variant 2, mRNA [NM_017986]	NM_017986	2.47
A_24_P225534	*RHBDL2*	Homo sapiens rhomboid, veinlet-like 2 (Drosophila) (RHBDL2), mRNA [NM_017821]	NM_017821	2.45
A_23_P422240	*UROC1*	Homo sapiens urocanase domain containing 1 (UROC1), transcript variant 1, mRNA [NM_144639]	NM_144639	2.43
A_23_P167509	*CYFIP2*	Homo sapiens cytoplasmic FMR1 interacting protein 2 (CYFIP2), transcript variant 2, mRNA [NM_001037332]	NM_001037332	2.43
A_23_P152906	*ALOX12*	Homo sapiens arachidonate 12-lipoxygenase (ALOX12), mRNA [NM_000697]	NM_000697	2.42
A_23_P257786	*ECE2*	Homo sapiens endothelin converting enzyme 2 (ECE2), transcript variant 1, mRNA [NM_014693]	NM_014693	2.42
A_24_P891472				2.40
A_24_P256219	*MAF*	Homo sapiens short form transcription factor C-MAF (c-maf) mRNA, complete cds. [AF055376]	AF055376	2.40
A_24_P646279		AW998865 PM4-BN0067-240300-001-e11 BN0067 Homo sapiens cDNA, mRNA sequence [AW998865]	AW998865	2.39
A_23_P21363	*AHNAK*	Homo sapiens AHNAK nucleoprotein (AHNAK), transcript variant 2, mRNA [NM_024060]	NM_024060	2.39
A_23_P135486	*AHSP*	Homo sapiens alpha hemoglobin stabilizing protein (AHSP), mRNA [NM_016633]	NM_016633	2.38
A_23_P32115	*LCN12*	Homo sapiens lipocalin 12 (LCN12), mRNA [NM_178536]	NM_178536	2.38
A_24_P693433		Homo sapiens cDNA FLJ12302 fis, clone MAMMA1001864. [AK022364]	AK022364	2.38
A_23_P252874	*GPR116*	Homo sapiens G protein-coupled receptor 116 (GPR116), transcript variant 1, mRNA [NM_015234]	NM_015234	2.38
A_32_P222106				2.37
A_32_P213469				2.37
A_24_P99175	*ZDHHC22*	Homo sapiens zinc finger, DHHC-type containing 22 (ZDHHC22), mRNA [NM_174976]	NM_174976	2.37
A_32_P441492		Homo sapiens cDNA FLJ32062 fis, clone OCBBF1000042. [AK056624]	AK056624	2.36
A_23_P56050	*TNNT1*	Homo sapiens troponin T type 1 (skeletal, slow) (TNNT1), transcript variant 2, mRNA [NM_001126132]	NM_001126132	2.35
A_23_P42746	*NCF1*	Homo sapiens neutrophil cytosolic factor 1 (NCF1), mRNA [NM_000265]	NM_000265	2.34
A_32_P332317		Homo sapiens cDNA FLJ12532 fis, clone NT2RM4000200. [AK022594]	AK022594	2.33
A_23_P217269	*VSIG4*	Homo sapiens V-set and immunoglobulin domain containing 4 (VSIG4), transcript variant 1, mRNA	NM_007268	2.32
A_23_P99275	*KLRB1*	Homo sapiens killer cell lectin-like receptor subfamily B, member 1 (KLRB1), mRNA [NM_002258]	NM_002258	2.32
A_24_P920795		Q504S0_HUMAN (Q504S0) MGC3196 protein (Fragment), partial (49%) [THC2529417]	BI771744	2.32
A_24_P8165	*SLC15A1*	Homo sapiens mRNA for pH-sensing regulatory factor of peptide transporter, complete cds. [AB001328]	AB001328	2.32
A_23_P312415	*ADAMTSL1*	Homo sapiens ADAMTS-like 1 (ADAMTSL1), transcript variant 2, mRNA [NM_052866]	NM_052866	2.31
A_23_P10091				2.31
A_24_P119774		phosphoglycerate kinase {alternatively spliced} [human, phosphoglycerate kinase deficient patient with episodes of muscl, mRNA Partial Mutant, 307 nt]. [S81916]	S81916	2.30
A_23_P111737	*RAMP3*	Homo sapiens receptor (G protein-coupled) activity modifying protein 3 (RAMP3), mRNA [NM_005856]	NM_005856	2.30
A_23_P122508	*DPCR1*	Homo sapiens diffuse panbronchiolitis critical region 1 (DPCR1), mRNA [NM_080870]	NM_080870	2.30
A_32_P709998	*OTOP2*	Homo sapiens otopetrin 2 (OTOP2), mRNA [NM_178160]	NM_178160	2.29
A_23_P59582	*SEMA3E*	Homo sapiens sema domain, immunoglobulin domain (Ig), short basic domain, secreted, (semaphorin) 3E (SEMA3E), mRNA [NM_012431]	NM_012431	2.29
A_24_P352388	*CDHR5*	Homo sapiens cadherin-related family member 5 (CDHR5), transcript variant 3, mRNA [NM_031264]	NM_031264	2.28
A_24_P196216	*LOXHD1*	Homo sapiens lipoxygenase homology domains 1 (LOXHD1), transcript variant 1, mRNA [NM_144612]	NM_144612	2.28
A_24_P323868	*LOC100130456*	Homo sapiens cDNA FLJ37693 fis, clone BRHIP2014954. [AK095012]	AK095012	2.27
A_32_P384562	*CROCCL2*	Homo sapiens ciliary rootlet coiled-coil, rootletin-like 2 (CROCCL2), non-coding RNA [NR_023386]	NR_023386	2.27
A_23_P303238	*VN1R5*	Homo sapiens vomeronasal 1 receptor 5 (gene/pseudogene) (VN1R5), mRNA [NM_173858]	NM_173858	2.27
A_23_P306859	*ZIC4*	Homo sapiens Zic family member 4 (ZIC4), transcript variant 3, mRNA [NM_032153]	NM_032153	2.27
A_24_P203418		immunoglobulin heavy variable 2-70 [Source:HGNC Symbol;Acc:5577] [ENST00000390634]	AF174045	2.26
A_24_P123190	*PLD1*	Homo sapiens phospholipase D1, phosphatidylcholine-specific (PLD1), transcript variant 1, mRNA	NM_002662	2.26
A_24_P125561	*SIGLEC9*	Homo sapiens sialic acid binding Ig-like lectin 9 (SIGLEC9), mRNA [NM_014441]	NM_014441	2.25
A_24_P102053	*OCLN*	Homo sapiens occludin (OCLN), mRNA [NM_002538]	NM_002538	2.25
A_24_P945375				2.25
A_24_P349807	*FAM71E2*	Homo sapiens family with sequence similarity 71, member E2 (FAM71E2), mRNA [NM_001145402]	NM_001145402	2.25
A_32_P155832				2.24
A_32_P914221	*USH1G*	Homo sapiens Usher syndrome 1G (autosomal recessive) (USH1G), mRNA [NM_173477]	NM_173477	2.24
A_23_P391946	*C18orf15*	Homo sapiens cDNA FLJ31338 fis, clone MAMGL1000184. [AK055900]	AK055900	2.24
A_24_P374532	*RIMS4*	Homo sapiens regulating synaptic membrane exocytosis 4 (RIMS4), mRNA [NM_182970]	NM_182970	2.23
A_23_P383009	*IGFBP5*	Homo sapiens insulin-like growth factor binding protein 5 (IGFBP5), mRNA [NM_000599]	NM_000599	2.22
A_23_P217341	*MAGEB1*	Homo sapiens melanoma antigen family B, 1 (MAGEB1), transcript variant 1, mRNA [NM_002363]	NM_002363	2.22
A_23_P320261	*DMKN*	Homo sapiens dermokine (DMKN), transcript variant 1, mRNA [NM_001035516]	NM_001035516	2.21
A_24_P919224				2.21
A_23_P26582	*GNAO1*	Homo sapiens guanine nucleotide binding protein (G protein), alpha activating activity polypeptide O (GNAO1), transcript variant 1, mRNA [NM_020988]	NM_020988	2.21
A_23_P145336	*HLA-DRB3*	Human MHC class II HLA-DR-beta mRNA (DR3), partial cds. [M17380]	M17380	2.20
A_23_P66073		Homo sapiens PRO0806 mRNA, complete cds. [AF113013]	AF113013	2.20
A_24_P910688	*C19orf67*	UPF0575 protein C19orf67 [Source:UniProtKB/Swiss-Prot;Acc:A6NJJ6] [ENST00000343945]	XM_929382	2.20
A_32_P90730		BX110500 Soares_pregnant_uterus_NbHPU Homo sapiens cDNA clone IMAGp998G171156, mRNA sequence [BX110500]	BX110500	2.20
A_32_P199220		Homo sapiens cDNA FLJ30583 fis, clone BRAWH2007406. [AK055145]	AK055145	2.19
A_23_P28772	*DBNDD2*	Homo sapiens dysbindin (dystrobrevin binding protein 1) domain containing 2 (DBNDD2), transcript variant 6, mRNA [NM_001048226]	NM_001048226	2.19
A_23_P156550		triggering receptor expressed on myeloid cells-like 1 [Source:HGNC Symbol;Acc:20434] [ENST00000437044]	AY358357	2.19
A_24_P922430				2.19
A_32_P121751		Q5SLK5_THET8 (Q5SLK5) 2-oxoglutarate dehydrogenase E2 component (Dihydrolipoamide succinyltransferase), partial (5%) [THC2765213]		2.17
A_32_P210168	*C15orf59*	Homo sapiens chromosome 15 open reading frame 59 (C15orf59), mRNA [NM_001039614]	NM_001039614	2.16
A_24_P239515		Homo sapiens cDNA clone IMAGE:4818334. [BC045731]	BC045731	2.16
A_23_P201687	*HES2*	Homo sapiens hairy and enhancer of split 2 (Drosophila), mRNA (cDNA clone IMAGE:4634002), complete cds. [BC012091]	BC012091	2.15
A_32_P26461		BX112876 NCI_CGAP_Co8 Homo sapiens cDNA clone IMAGp998D133952, mRNA sequence [BX112876]	BX112876	2.15
A_24_P557351		Homo sapiens, clone IMAGE:3344449, mRNA. [BC025324]	BC025324	2.15
A_23_P408285	*PRICKLE1*	Homo sapiens prickle homolog 1 (Drosophila) (PRICKLE1), transcript variant 1, mRNA [NM_153026]	NM_153026	2.15
A_24_P44453	*DUOXA1*	Homo sapiens cDNA FLJ32334 fis, clone PROST2005426. [AK056896]	AK056896	2.14
A_23_P115726	*SLC16A9*	Homo sapiens solute carrier family 16, member 9 (monocarboxylic acid transporter 9) (SLC16A9), mRNA	NM_194298	2.14
A_32_P102935	*SPDYA*	Homo sapiens speedy homolog A (Xenopus laevis) (SPDYA), transcript variant 2, mRNA [NM_001008779]	NM_001008779	2.14
A_32_P53093		Q3JKQ7_BURP1 (Q3JKQ7) SctT, partial (4%) [THC2664135]		2.14
A_24_P247902	*PCLO*	Homo sapiens piccolo (presynaptic cytomatrix protein) (PCLO), transcript variant 2, mRNA [NM_014510]	NM_014510	2.13
A_24_P923432		UI-H-BI1-abx-a-01-0-UI.s1 NCI_CGAP_Sub3 Homo sapiens cDNA clone IMAGE:2713225 3', mRNA sequence [AW138500]	AW138500	2.13
A_24_P881562				2.13
A_23_P113738		Homo sapiens CES hBr3 mRNA for brain carboxylesterase hBr3, complete cds. [AB025028]	AB025028	2.12
A_32_P45285				2.12
A_32_P212939	*ZNF726*	zinc finger protein 726 [Source:HGNC Symbol;Acc:32462] [ENST00000322487]	XM_001726951	2.11
A_23_P307955	*SIRT5*	Homo sapiens sirtuin (silent mating type information regulation 2 homolog) 5 (S. cerevisiae) (SIRT5), transcript variant 2, mRNA [NM_031244]	NM_031244	2.11
A_23_P68669	*CHODL*	Homo sapiens chondrolectin (CHODL), mRNA [NM_024944]	NM_024944	2.11
A_24_P887857				2.11

A_23_P201863	*CDK18*	Homo sapiens cyclin-dependent kinase 18 (CDK18), transcript variant 1, mRNA [NM_212503]	NM_212503	2.10
A_23_P82775	*SOX17*	Homo sapiens SRY (sex determining region Y)-box 17 (SOX17), mRNA [NM_022454]	NM_022454	2.10
A_24_P801825				2.10
A_23_P78265	*KRT33A*	Homo sapiens keratin 33A (KRT33A), mRNA [NM_004138]	NM_004138	2.10
A_24_P289265		ATP-binding cassette, sub-family A (ABC1), member 1 [Source:HGNC Symbol;Acc:29] [ENST00000341579]	AK024328	2.10
A_23_P102634	*WFDC6*	Homo sapiens WAP four-disulfide core domain 6 (WFDC6), mRNA [NM_080827]	NM_080827	2.10
A_23_P351757	*PLCD3*	Homo sapiens phospholipase C, delta 3 (PLCD3), mRNA [NM_133373]	NM_133373	2.10
A_24_P467871				2.09
A_23_P1473	*PRF1*	Homo sapiens perforin 1 (pore forming protein) (PRF1), transcript variant 1, mRNA [NM_005041]	NM_005041	2.09
A_24_P492885				2.09
A_23_P315425	*FLJ31958*	Homo sapiens cDNA FLJ31958 fis, clone NT2RP7007406. [AK056520]	AK056520	2.09
A_32_P469683		Homo sapiens clone FLB5227 PRO1367 mRNA, complete cds. [AF130056]	AF130056	2.09
A_23_P69179	*LEPREL1*	Homo sapiens leprecan-like 1 (LEPREL1), transcript variant 1, mRNA [NM_018192]	NM_018192	2.09
A_24_P24371		immunoglobulin heavy constant gamma 4 (G4m marker) [Source:HGNC Symbol;Acc:5528]	AJ294733	2.08
A_23_P57020	*GTSF1L*	Homo sapiens gametocyte specific factor 1-like (GTSF1L), transcript variant 2, mRNA [NM_001008901]	NM_001008901	2.08
A_23_P137751	*FAM46C*	Homo sapiens family with sequence similarity 46, member C (FAM46C), mRNA [NM_017709]	NM_017709	2.08
A_23_P78543	*AP1M2*	Homo sapiens adaptor-related protein complex 1, mu 2 subunit (AP1M2), mRNA [NM_005498]	NM_005498	2.08
A_23_P376704	*CIDEA*	Homo sapiens cell death-inducing DFFA-like effector a (CIDEA), transcript variant 2, mRNA [NM_198289]	NM_198289	2.07
A_24_P363609	*IRF5*	Homo sapiens interferon regulatory factor 5 (IRF5), transcript variant 3, mRNA [NM_001098627]	NM_001098627	2.06
A_23_P13455	*OR51A7*	Homo sapiens olfactory receptor, family 51, subfamily A, member 7 (OR51A7), mRNA [NM_001004749]	NM_001004749	2.06
A_23_P145978	*VIPR2*	Homo sapiens vasoactive intestinal peptide receptor 2 (VIPR2), mRNA [NM_003382]	NM_003382	2.05
A_24_P911718		Homo sapiens cDNA clone IMAGE:5278089. [BC037919]	BC037919	2.05
A_23_P167464		Homo sapiens cDNA FLJ12078 fis, clone HEMBB1002457. [AK022140]	AK022140	2.05
A_23_P153185	*SERPINB2*	Homo sapiens serpin peptidase inhibitor, clade B (ovalbumin), member 2 (SERPINB2), transcript variant 1, mRNA [NM_001143818]	NM_001143818	2.05
A_23_P52868	*OR8G2*	Homo sapiens olfactory receptor, family 8, subfamily G, member 2, mRNA (cDNA clone MGC:168251 IMAGE:9020628), complete cds. [BC136638]	BC136638	2.05
A_24_P67741	*LOC100131554*	CDNA: FLJ23120 fis, clone LNG07989HCG1987724 ; [Source:UniProtKB/TrEMBL;Acc:Q9H5S2]	AK026773	2.04
A_24_P126406	*DNAH17*	Homo sapiens dynein, axonemal, heavy chain 17 (DNAH17), mRNA [NM_173628]	NM_173628	2.04
A_24_P464701				2.04
A_23_P92672	*OCLN*	Homo sapiens occludin (OCLN), mRNA [NM_002538]	NM_002538	2.04
A_24_P410610	*DPYD*	Homo sapiens dihydropyrimidine dehydrogenase (DPYD), transcript variant 2, mRNA [NM_001160301]	NM_001160301	2.03
A_32_P21848		ALU5_HUMAN (P39192) Alu subfamily SC sequence contamination warning entry, partial (5%) [THC2716178]		2.03
A_24_P332651	*LOC650157*	PREDICTED: Homo sapiens similar to TRIM5/CypA fusion protein (LOC650157), mRNA [XM_001726994]	XM_001726994	2.03
A_32_P15544	*PRIMA1*	Homo sapiens proline rich membrane anchor 1 (PRIMA1), mRNA [NM_178013]	NM_178013	2.02
A_23_P500501	*FGFR3*	Homo sapiens fibroblast growth factor receptor 3 (FGFR3), transcript variant 1, mRNA [NM_000142]	NM_000142	2.02
A_23_P72330				2.02
A_23_P308042	*C6orf195*	Homo sapiens chromosome 6 open reading frame 195 (C6orf195), mRNA [NM_152554]	NM_152554	2.02
A_32_P46571	*RHBDL2*	Homo sapiens rhomboid, veinlet-like 2 (Drosophila) (RHBDL2), mRNA [NM_017821]	NM_017821	2.02
A_32_P10886	*C6orf52*	Homo sapiens chromosome 6 open reading frame 52 (C6orf52), transcript variant 1, mRNA [NM_001145020]	NM_001145020	2.02
A_24_P478745		fh11b09.y1 NIH_MGC_17 Homo sapiens cDNA clone IMAGE:2964448 3', mRNA sequence [AW411241]	AW411241	2.02
A_23_P93940	*PGAM2*	Homo sapiens phosphoglycerate mutase 2 (muscle) (PGAM2), mRNA [NM_000290]	NM_000290	2.02
A_23_P133637	*HTR4*	Homo sapiens 5-hydroxytryptamine (serotonin) receptor 4 (HTR4), transcript variant d, mRNA [NM_001040171]	NM_001040171	2.01
A_23_P84230	*OTP*	Homo sapiens orthopedia homeobox (OTP), mRNA [NM_032109]	NM_032109	2.01
A_24_P943263	*RASA4*	Homo sapiens RAS p21 protein activator 4 (RASA4), transcript variant 1, mRNA [NM_006989]	NM_006989	2.01
A_23_P49448	*FA2H*	Homo sapiens fatty acid 2-hydroxylase (FA2H), mRNA [NM_024306]	NM_024306	2.01
A_32_P99399				2.00
A_32_P88163		ALU5_HUMAN (P39192) Alu subfamily SC sequence contamination warning entry, partial (9%) [THC2633438]		-2.00
A_23_P140316	*GPR137C*	Homo sapiens G protein-coupled receptor 137C (GPR137C), mRNA [NM_001099652]	NM_001099652	-2.01
A_24_P229447		Putative uncharacterized protein ENSP00000346720 Fragment [Source:UniProtKB/TrEMBL;Acc:A6NG04] [ENST00000354689]	AY944711	-2.01
A_24_P62668	*SERTAD4*	SERTA domain containing 4 [Source:HGNC Symbol;Acc:25236] [ENST00000367012]	AK021425	-2.01
A_32_P220109		BE924986 MR1-AN0036-280800-002-f08 AN0036 Homo sapiens cDNA, mRNA sequence [BE924986]	BE924986	-2.03
A_24_P257511	*BHMT*	Homo sapiens betaine-homocysteine methyltransferase (BHMT), mRNA [NM_001713]	NM_001713	-2.03
A_23_P434968	*KLF17*	Homo sapiens Kruppel-like factor 17 (KLF17), mRNA [NM_173484]	NM_173484	-2.04
A_23_P14083	*AMIGO2*	Homo sapiens adhesion molecule with Ig-like domain 2 (AMIGO2), transcript variant 2, mRNA [NM_181847]	NM_181847	-2.05
A_24_P396753	*TRIB2*	Homo sapiens tribbles homolog 2 (Drosophila) (TRIB2), transcript variant 1, mRNA [NM_021643]	NM_021643	-2.05
A_23_P67799	*TMEM37*	Homo sapiens transmembrane protein 37 (TMEM37), mRNA [NM_183240]	NM_183240	-2.06
A_23_P51082		ALU1_HUMAN (P39188) Alu subfamily J sequence contamination warning entry, partial (4%) [THC2488254]		-2.07
A_23_P167066	*UGDH*	Homo sapiens UDP-glucose dehydrogenase (UGDH), mRNA [NM_003359]	NM_003359	-2.07
A_23_P209625	*CYP1B1*	Homo sapiens cytochrome P450, family 1, subfamily B, polypeptide 1 (CYP1B1), mRNA [NM_000104]	NM_000104	-2.07
A_23_P156431	*MAN1A1*	Homo sapiens mannosidase, alpha, class 1A, member 1 (MAN1A1), mRNA [NM_005907]	NM_005907	-2.08
A_24_P342670	*GON4L*	Homo sapiens cDNA: FLJ23040 fis, clone LNG02277. [AK026693]	AK026693	-2.09
A_32_P125338	*FAM43B*	Homo sapiens family with sequence similarity 43, member B (FAM43B), mRNA [NM_207334]	NM_207334	-2.09
A_32_P184746		BX090390 Soares fetal liver spleen 1NFLS Homo sapiens cDNA clone IMAGp998I10652 ; IMAGE:292689, mRNA sequence [BX090390]	BX090390	-2.09
A_24_P362805	*GK5*	Homo sapiens glycerol kinase 5 (putative) (GK5), transcript variant 2, non-coding RNA [NR_033289]	NR_033289	-2.10
A_23_P133536	*CAPSL*	Homo sapiens calcyphosine-like (CAPSL), transcript variant 1, mRNA [NM_144647]	NM_144647	-2.12
A_32_P135634		Q2RZ67_SALRD (Q2RZ67) Glycosyl transferase, group 1 family protein , partial (6%) [THC2643352]		-2.12
A_23_P145606	*CHRM2*	Homo sapiens cholinergic receptor, muscarinic 2 (CHRM2), transcript variant 1, mRNA [NM_001006630]	NM_001006630	-2.12
A_23_P47148	*NOX4*	Homo sapiens NADPH oxidase 4 (NOX4), transcript variant 1, mRNA [NM_016931]	NM_016931	-2.13
A_24_P910255				-2.13
A_23_P13740	*NAV3*	Homo sapiens neuron navigator 3 (NAV3), mRNA [NM_014903]	NM_014903	-2.13
A_24_P68006	*MOBKL2B*	Homo sapiens cDNA FLJ23916 fis, clone COL00117. [AK172755]	AK172755	-2.14
A_23_P351215	*SKIL*	Homo sapiens SKI-like oncogene (SKIL), transcript variant 1, mRNA [NM_005414]	NM_005414	-2.15
A_23_P202013	*ZEB1*	Homo sapiens zinc finger E-box binding homeobox 1 (ZEB1), transcript variant 2, mRNA [NM_030751]	NM_030751	-2.15
A_23_P112634	*C4orf34*	Homo sapiens chromosome 4 open reading frame 34 (C4orf34), mRNA [NM_174921]	NM_174921	-2.15
A_23_P156928	*GPLD1*	Homo sapiens glycosylphosphatidylinositol specific phospholipase D1 (GPLD1), transcript variant 1, mRNA	NM_001503	-2.15
A_32_P167111				-2.15
A_23_P196188	*NXPH4*	Homo sapiens neurexophilin 4 (NXPH4), mRNA [NM_007224]	NM_007224	-2.15
A_24_P689119				-2.16
A_24_P123119	*EHHADH*	Homo sapiens enoyl-Coenzyme A, hydratase/3-hydroxyacyl Coenzyme A dehydrogenase (EHHADH), transcript variant 1, mRNA [NM_001966]	NM_001966	-2.17
A_23_P65918	*ITPKA*	Homo sapiens inositol 1,4,5-trisphosphate 3-kinase A (ITPKA), mRNA [NM_002220]	NM_002220	-2.17
A_23_P301925	*COX1*	Cytochrome c oxidase subunit 1 (EC 1.9.3.1)(Cytochrome c oxidase polypeptide I) [Source:UniProtKB/Swiss-Prot;Acc:P00395] [ENST00000361624]	CR606568	-2.17
A_23_P390621	*PACRGL*	Homo sapiens PARK2 co-regulated-like (PACRGL), transcript variant 1, mRNA [NM_145048]	NM_145048	-2.18
A_23_P155900	*NPFFR2*	Homo sapiens neuropeptide FF receptor 2 (NPFFR2), transcript variant 2, mRNA [NM_053036]	NM_053036	-2.19
A_23_P165457	*CRYBA2*	Homo sapiens crystallin, beta A2 (CRYBA2), transcript variant 1, mRNA [NM_005209]	NM_005209	-2.19
A_23_P65307	*SLITRK6*	Homo sapiens SLIT and NTRK-like family, member 6 (SLITRK6), mRNA [NM_032229]	NM_032229	-2.20
A_24_P920904	*C8orf59*	Homo sapiens chromosome 8 open reading frame 59, mRNA (cDNA clone IMAGE:5212747), complete cds.	BC032347	-2.23
A_32_P174365	*SATB2*	Homo sapiens SATB homeobox 2 (SATB2), transcript variant 2, mRNA [NM_015265]	NM_015265	-2.24
A_24_P697043		BX091936 Soares placenta Nb2HP Homo sapiens cDNA clone IMAGp998N02193 ; IMAGE:135745, mRNA sequence [BX091936]	BX091936	-2.24
A_24_P85045	*PNPLA8*	Homo sapiens patatin-like phospholipase domain containing 8 (PNPLA8), mRNA [NM_015723]	NM_015723	-2.25
A_24_P702020		AA195394 zp87f04.s1 Stratagene HeLa cell s3 937216 Homo sapiens cDNA clone IMAGE:627199 3' similar to contains Alu repetitive element;, mRNA sequence [AA195394]	AA195394	-2.25
A_32_P91906		Synthetic construct Homo sapiens gateway clone IMAGE:100018321 3' read MPP1 mRNA. [CU677943]	CU677943	-2.26
A_32_P139021				-2.27
A_24_P221660	*ZBTB41*	Homo sapiens zinc finger and BTB domain containing 41 (ZBTB41), mRNA [NM_194314]	NM_194314	-2.27
A_24_P852099				-2.27
A_24_P462656	*TACC2*	Homo sapiens transforming, acidic coiled-coil containing protein 2, mRNA (cDNA clone IMAGE:3450479), with apparent retained intron. [BC010219]	BC010219	-2.28

A_24_P33446	*LOC338651*	Homo sapiens hypothetical protein LOC338651 (LOC338651), non-coding RNA [NR_021489]	NR_021489	-2.28
A_24_P83798	*ALX1*	Homo sapiens ALX homeobox 1 (ALX1), mRNA [NM_006982]	NM_006982	-2.28
A_24_P930100	*CENPL*	Homo sapiens cDNA FLJ31786 fis, clone NT2RI2008526. [AK056348]	AK056348	-2.29
A_23_P371276	*RNF165*	Homo sapiens cDNA FLJ90080 fis, clone HEMBA1004797. [AK074561]	AK074561	-2.31
A_24_P930448		tj45f08.x1 Soares_NSF_F8_9W_OT_PA_P_S1 Homo sapiens cDNA clone IMAGE:2144487 3' similar to contains Alu repetitive element;contains element KER repetitive element ;, mRNA sequence [AI453629]	AI453629	-2.31
A_23_P409553	*PPM1A*	Homo sapiens protein phosphatase, Mg2+/Mn2+ dependent, 1A (PPM1A), transcript variant 2, mRNA	NM_177951	-2.33
A_24_P121406	*ZC3H13*	Homo sapiens zinc finger CCCH-type containing 13 (ZC3H13), mRNA [NM_015070]	NM_015070	-2.34
A_24_P922631	*C5orf58*	Homo sapiens chromosome 5 open reading frame 58 (C5orf58), mRNA [NM_001102609]	NM_001102609	-2.34
A_24_P582324		AA687366 nv62g05.s1 NCI_CGAP_GCB1 Homo sapiens cDNA clone IMAGE:1234424 3' similar to contains Alu repetitive element;, mRNA sequence [AA687366]	AA687366	-2.35
A_32_P41099				-2.36
A_24_P94351	*C1orf56*	Homo sapiens chromosome 1 open reading frame 56 (C1orf56), mRNA [NM_017860]	NM_017860	-2.36
A_23_P356717	*ANKS1B*	Homo sapiens ankyrin repeat and sterile alpha motif domain containing 1B (ANKS1B), transcript variant 2, mRNA [NM_181670]	NM_181670	-2.36
A_24_P512054	*DDX50*	Homo sapiens DEAD (Asp-Glu-Ala-Asp) box polypeptide 50 (DDX50), mRNA [NM_024045]	NM_024045	-2.37
A_32_P200303	*tcag7.1307*	Homo sapiens hypothetical LOC154822 (LOC154822), non-coding RNA [NR_024394]	NR_024394	-2.37
A_24_P927311				-2.38
A_23_P203702		Q214U3_RHOPA (Q214U3) Penicillin-binding protein 1C precursor, partial (3%) [THC2707284]		-2.38
A_24_P74508	*SLC25A18*	Homo sapiens solute carrier family 25 (mitochondrial carrier), member 18 (SLC25A18), nuclear gene encoding mitochondrial protein, mRNA [NM_031481]	NM_031481	-2.39
A_32_P223661		as48d05.x1 Barstead aorta HPLRB6 Homo sapiens cDNA clone IMAGE:2320425 3', mRNA sequence	AI719994	-2.40
A_24_P273742	*TTC9*	Homo sapiens tetratricopeptide repeat domain 9 (TTC9), mRNA [NM_015351]	NM_015351	-2.40
A_23_P6822	*ITIH3*	Homo sapiens inter-alpha (globulin) inhibitor H3 (ITIH3), mRNA [NM_002217]	NM_002217	-2.41
A_23_P168610	*TSPAN13*	Homo sapiens tetraspanin 13 (TSPAN13), mRNA [NM_014399]	NM_014399	-2.41
A_23_P69206	*SLC6A6*	Homo sapiens solute carrier family 6 (neurotransmitter transporter, taurine), member 6 (SLC6A6), transcript variant 1, mRNA [NM_003043]	NM_003043	-2.42
A_23_P201179	*PHTF1*	Homo sapiens mRNA for homeobox protein LSX. [AJ011863]	AJ011863	-2.43
A_23_P1387	*ARHGAP19*	Homo sapiens Rho GTPase activating protein 19 (ARHGAP19), mRNA [NM_032900]	NM_032900	-2.43
A_24_P402510	*SAMD11*	Homo sapiens sterile alpha motif domain containing 11 (SAMD11), mRNA [NM_152486]	NM_152486	-2.46
A_32_P131342				-2.46
A_23_P136573	*ST3GAL5*	Homo sapiens ST3 beta-galactoside alpha-2,3-sialyltransferase 5 (ST3GAL5), transcript variant 1, mRNA	NM_003896	-2.48
A_23_P134566	*OR2A7*	Homo sapiens olfactory receptor, family 2, subfamily A, member 7 (OR2A7), mRNA [NM_001005328]	NM_001005328	-2.48
A_23_P417014	*ZNF311*	Homo sapiens zinc finger protein 311 (ZNF311), mRNA [NM_001010877]	NM_001010877	-2.50
A_32_P197621	*GEMIN8P4*	Homo sapiens gem (nuclear organelle) associated protein 8 pseudogene 4 (GEMIN8P4), non-coding RNA	NR_002830	-2.51
A_23_P131394	*SLC16A14*	Homo sapiens solute carrier family 16, member 14 (monocarboxylic acid transporter 14) (SLC16A14), mRNA [NM_152527]	NM_152527	-2.51
A_32_P2807		Homo sapiens cDNA clone IMAGE:4361039, partial cds. [BC064349]	BC064349	-2.52
A_24_P359100	*LOC100130193*	Homo sapiens cDNA FLJ38783 fis, clone LIVER2001191. [AK096102]	AK096102	-2.52
A_32_P23187	*LOC100127984*	PREDICTED: Homo sapiens hypothetical LOC100127984 (LOC100127984), mRNA [XM_001719916]	XM_001719916	-2.53
A_23_P131208	*NR4A2*	Homo sapiens nuclear receptor subfamily 4, group A, member 2 (NR4A2), mRNA [NM_006186]	NM_006186	-2.54
A_24_P798431				-2.56
A_24_P192933	*UBASH3B*	Homo sapiens ubiquitin associated and SH3 domain containing, B (UBASH3B), mRNA [NM_032873]	NM_032873	-2.58
A_23_P109934	*HEMK1*	Homo sapiens HemK methyltransferase family member 1 (HEMK1), mRNA [NM_016173]	NM_016173	-2.58
A_24_P402438	*TGFB2*	Homo sapiens transforming growth factor, beta 2 (TGFB2), transcript variant 2, mRNA [NM_003238]	NM_003238	-2.60
A_23_P160582	*HYI*	Homo sapiens hydroxypyruvate isomerase homolog (E. coli) (HYI), transcript variant 1, mRNA [NM_031207]	NM_031207	-2.62
A_24_P926382		Homo sapiens full length insert cDNA clone ZE05D05. [AF086528]	AF086528	-2.63
A_23_P104237	*GPAM*	Homo sapiens glycerol-3-phosphate acyltransferase, mitochondrial (GPAM), nuclear gene encoding mitochondrial protein, mRNA [NM_020918]	NM_020918	-2.65
A_23_P87952	*SOX5*	Homo sapiens SRY (sex determining region Y)-box 5 (SOX5), transcript variant 2, mRNA [NM_152989]	NM_152989	-2.68
A_23_P15844	*BRIP1*	Homo sapiens BRCA1 interacting protein C-terminal helicase 1 (BRIP1), mRNA [NM_032043]	NM_032043	-2.68
A_32_P75399		HUMSEF21A SEF2-1A protein {Homo sapiens} (exp=-1; wgp=0; cg=0), partial (5%) [THC2551774]		-2.68
A_32_P232198				-2.72
A_32_P124887				-2.72
A_23_P258612	*ATP8A2*	Homo sapiens ATPase, aminophospholipid transporter-like, class I, type 8A, member 2 (ATP8A2), mRNA	NM_016529	-2.74
A_32_P122494		7e14b03.x1 NCI_CGAP_Lu24 Homo sapiens cDNA clone IMAGE:3282413 3' similar to SW:NMA_HUMAN Q13145 PUTATIVE TRANSMEMBRANE PROTEIN NMA PRECURSOR. ;, mRNA sequence [BE669520]	BE669520	-2.77
A_23_P200976	*HYI*	Homo sapiens hydroxypyruvate isomerase homolog (E. coli) (HYI), transcript variant 1, mRNA [NM_031207]	NM_031207	-2.78
A_32_P99347	*C9orf110*	Homo sapiens chromosome 9 open reading frame 110 (C9orf110), non-coding RNA [NR_024376]	NR_024376	-2.79
A_23_P145644	*DDC*	Homo sapiens dopa decarboxylase (aromatic L-amino acid decarboxylase) (DDC), transcript variant 2, mRNA [NM_000790]	NM_000790	-2.81
A_32_P229132	*FMN2*	Homo sapiens formin 2 (FMN2), mRNA [NM_020066]	NM_020066	-2.81
A_23_P108492	*MARS2*	Homo sapiens methionyl-tRNA synthetase 2, mitochondrial (MARS2), nuclear gene encoding mitochondrial protein, mRNA [NM_138395]	NM_138395	-2.82
A_24_P944928		BI047192 RC5-FT0194-050101-015-D09 FT0194 Homo sapiens cDNA, mRNA sequence [BI047192]	BI047192	-2.83
A_23_P159325	*ANGPTL4*	Homo sapiens angiopoietin-like 4 (ANGPTL4), transcript variant 1, mRNA [NM_139314]	NM_139314	-2.85
A_24_P225862		yi73c12.r1 Soares placenta Nb2HP Homo sapiens cDNA clone IMAGE:144886 3', mRNA sequence [R78584]	R78584	-2.88
A_23_P5654	*IL1F7*	Homo sapiens interleukin 1 family, member 7 (zeta) (IL1F7), transcript variant 1, mRNA [NM_014439]	NM_014439	-2.91
A_24_P212926	*LOC728800*	Homo sapiens mRNA for FLJ00402 protein. [AK090480]	AK090480	-2.91
A_23_P170719				-2.96
A_23_P162171	*MCAM*	Homo sapiens melanoma cell adhesion molecule (MCAM), mRNA [NM_006500]	NM_006500	-2.97
A_24_P326660	*MCAM*	Homo sapiens melanoma cell adhesion molecule (MCAM), mRNA [NM_006500]	NM_006500	-2.97
A_23_P201181	*PTPN22*	Homo sapiens protein tyrosine phosphatase, non-receptor type 22 (lymphoid) (PTPN22), transcript variant 2, mRNA [NM_012411]	NM_012411	-3.01
A_32_P166031		Homo sapiens cDNA clone IMAGE:6166085, partial cds. [BC052811]	BC052811	-3.04
A_32_P437735	*LOC256021*	Homo sapiens cDNA FLJ30877 fis, clone FEBRA2004443. [AK055439]	AK055439	-3.09
A_32_P119726	*DST*	Homo sapiens cDNA FLJ54409 complete cds, highly similar to Bullous pemphigoid antigen 1 isoforms 1/2/3/4/5/8. [AK295864]	AK295864	-3.09
A_32_P31963		Q9BVD9_HUMAN (Q9BVD9) FTO protein, partial (9%) [THC2527460]		-3.09
A_24_P709476	*LOC150568*	Homo sapiens hypothetical LOC150568 (LOC150568), non-coding RNA [NR_015399]	NR_015399	-3.10
A_24_P342829	*SLC16A14*	Homo sapiens solute carrier family 16, member 14 (monocarboxylic acid transporter 14) (SLC16A14), mRNA [NM_152527]	NM_152527	-3.17
A_23_P5131	*ISYNA1*	Homo sapiens inositol-3-phosphate synthase 1 (ISYNA1), transcript variant 1, mRNA [NM_016368]	NM_016368	-3.24
A_23_P325690	*ANKRD35*	Homo sapiens ankyrin repeat domain 35 (ANKRD35), mRNA [NM_144698]	NM_144698	-3.36
A_32_P185682		full-length cDNA clone CS0DJ002YF02 of T cells (Jurkat cell line) Cot 10-normalized of Homo sapiens (human). [CR613361]	CR613361	-3.44
A_23_P48740	*DIO2*	Homo sapiens deiodinase, iodothyronine, type II (DIO2), transcript variant 1, mRNA [NM_013989]	NM_013989	-3.52
A_23_P148600	*INE1*	Homo sapiens inactivation escape 1 (non-protein coding) (INE1), non-coding RNA [NR_024616]	NR_024616	-3.54
A_23_P114414	*LONRF3*	Homo sapiens LON peptidase N-terminal domain and ring finger 3 (LONRF3), transcript variant 1, mRNA [NM_001031855]	NM_001031855	-3.59
A_23_P114466	*TBL1Y*	Homo sapiens transducin (beta)-like 1, Y-linked (TBL1Y), transcript variant 1, mRNA [NM_033284]	NM_033284	-3.64
A_23_P137504	*ZBTB37*	Homo sapiens zinc finger and BTB domain containing 37 (ZBTB37), transcript variant 2, mRNA [NM_032522]	NM_032522	-3.66
A_32_P144281				-3.71
A_23_P67278	*ZNF443*	Homo sapiens zinc finger protein 443 (ZNF443), mRNA [NM_005815]	NM_005815	-3.85
A_23_P118203	*ZG16B*	Homo sapiens zymogen granule protein 16 homolog B (rat) (ZG16B), mRNA [NM_145252]	NM_145252	-3.93
A_23_P211762	*COL8A1*	collagen, type VIII, alpha 1 [Source:HGNC Symbol;Acc:2215] [ENST00000261037]	CN371015	-3.96
A_23_P17655	*KCNJ15*	Homo sapiens potassium inwardly-rectifying channel, subfamily J, member 15 (KCNJ15), transcript variant 1, mRNA [NM_170736]	NM_170736	-3.96
A_23_P161458	*OLAH*	Homo sapiens oleoyl-ACP hydrolase (OLAH), transcript variant 2, mRNA [NM_001039702]	NM_001039702	-4.01
A_24_P243329	*ITGA2*	Homo sapiens integrin, alpha 2 (CD49B, alpha 2 subunit of VLA-2 receptor) (ITGA2), mRNA [NM_002203]	NM_002203	-4.13
A_24_P297302				-4.52
A_23_P38732	*CDH2*	Homo sapiens cadherin 2, type 1, N-cadherin (neuronal) (CDH2), mRNA [NM_001792]	NM_001792	-4.52
A_24_P40626	*GREM2*	Homo sapiens gremlin 2, cysteine knot superfamily, homolog (Xenopus laevis) (GREM2), mRNA [NM_022469]	NM_022469	-4.70

A_32_P934840	*C8orf34*	Homo sapiens chromosome 8 open reading frame 34 (C8orf34), mRNA [NM_052958]	NM_052958	-4.71
A_23_P86653	*SRGN*	Homo sapiens serglycin (SRGN), mRNA [NM_002727]	NM_002727	-5.25
A_24_P810290	*PPAPDC1A*	Homo sapiens phosphatidic acid phosphatase type 2 domain containing 1A (PPAPDC1A), mRNA	NM_001030059	-5.81
A_23_P69030	*COL8A1*	Homo sapiens collagen, type VIII, alpha 1 (COL8A1), transcript variant 1, mRNA [NM_001850]	NM_001850	-5.81
A_23_P365267	*SNED1*	Homo sapiens sushi, nidogen and EGF-like domains 1 (SNED1), mRNA [NM_001080437]	NM_001080437	-6.03
A_24_P185909	*LONRF3*	Homo sapiens LON peptidase N-terminal domain and ring finger 3 (LONRF3), transcript variant 1, mRNA [NM_001031855]	NM_001031855	-6.19
A_24_P139152	*COL8A1*	collagen, type VIII, alpha 1 [Source:HGNC Symbol;Acc:2215] [ENST00000261037]	AL359062	-6.35
A_23_P342869	*FMN2*	Homo sapiens formin 2 (FMN2), mRNA [NM_020066]	NM_020066	-6.80
A_32_P55241	*SHISA2*	Homo sapiens shisa homolog 2 (Xenopus laevis) (SHISA2), mRNA [NM_001007538]	NM_001007538	-6.92
A_23_P76386	*SLC6A12*	Homo sapiens solute carrier family 6 (neurotransmitter transporter, betaine/GABA), member 12 (SLC6A12), transcript variant 1, mRNA [NM_003044]	NM_003044	-7.51
A_23_P34597	*CDA*	Homo sapiens cytidine deaminase (CDA), mRNA [NM_001785]	NM_001785	-8.75
A_24_P334130	*FN1*	Homo sapiens fibronectin 1 (FN1), transcript variant 7, mRNA [NM_054034]	NM_054034	-10.12
A_23_P330611	*WIPF1*	Homo sapiens WAS/WASL interacting protein family, member 1 (WIPF1), transcript variant 2, mRNA	NM_001077269	-10.24
A_23_P63209	*HSD11B1*	Homo sapiens hydroxysteroid (11-beta) dehydrogenase 1 (HSD11B1), transcript variant 2, mRNA [NM_181755]	NM_181755	-11.93
