# Supplementary material for: Loss of the BRCA1-Interacting Helicase BRIP1 Results in Abnormal Mammary Acinar Morphogenesis
Source: PLoS One. 2013 Sep 6;8(9):e74013. doi: 10.1371/journal.pone.0074013 (PMC3765252; doi:10.1371/journal.pone.0074013)
Supplement: Table S2 — Up- and down-regulated genes (≥2-fold, P<0.05) in 3D culture of BRIP1-knockdown cells compared with control cells at day 8. (PDF) [file pone.0074013.s004.pdf]

Table S2. Up- and down-regulated genes (≥ 2-fold, P < 0.05) in 3D culture of *BRIP1*-knockdown cells compared with control cells at day 8

ProbeName	GeneSymbol	Description	GenbankAccession	Fold change
A_23_P26854	*RICH2*	Homo sapiens Rho-type GTPase-activating protein RICH2 (RICH2), mRNA [NM_014859]	NM_014859	64.54
A_23_P257003	*PCSK5*	Homo sapiens proprotein convertase subtilisin/kexin type 5 (PCSK5), mRNA [NM_006200]	NM_006200	25.79
A_23_P502413	*SERPINB4*	Homo sapiens serpin peptidase inhibitor, clade B (ovalbumin), member 4 (SERPINB4), mRNA [NM_002974]	NM_002974	9.64
A_23_P383258	*GDA*	Homo sapiens guanine deaminase (GDA), mRNA [NM_004293]	NM_004293	8.87
A_23_P259741	*SATB1*	Homo sapiens SATB homeobox 1 (SATB1), transcript variant 1, mRNA [NM_002971]	NM_002971	8.80
A_23_P202881	*FEZ1*	Homo sapiens fasciculation and elongation protein zeta 1 (zygin I) (FEZ1), transcript variant 1, mRNA	NM_005103	7.62
A_23_P107911	*KLK10*	Homo sapiens kallikrein-related peptidase 10 (KLK10), transcript variant 1, mRNA [NM_002776]	NM_002776	6.54
A_23_P212869	*GABRA2*	Homo sapiens gamma-aminobutyric acid (GABA) A receptor, alpha 2 (GABRA2), transcript variant 1, mRNA [NM_000807]	NM_000807	6.24
A_23_P167121	*GABRA2*	Homo sapiens gamma-aminobutyric acid (GABA) A receptor, alpha 2 (GABRA2), transcript variant 1, mRNA [NM_000807]	NM_000807	5.39
A_23_P50638	*LRG1*	Homo sapiens leucine-rich alpha-2-glycoprotein 1 (LRG1), mRNA [NM_052972]	NM_052972	5.31
A_24_P25063				4.48
A_23_P17065	*CCL20*	Homo sapiens chemokine (C-C motif) ligand 20 (CCL20), transcript variant 1, mRNA [NM_004591]	NM_004591	4.22
A_23_P110531	*FST*	Homo sapiens follistatin (FST), transcript variant FST344, mRNA [NM_013409]	NM_013409	4.22
A_32_P155841		Homo sapiens mRNA full length insert cDNA clone EUROIMAGE 362780. [AL079294]	AL079294	4.14
A_24_P913930		BY798802 Homo sapiens eye Homo sapiens cDNA clone HE3861.seq 5', mRNA sequence [BY798802]	BY798802	3.33
A_23_P57110	*C20orf54*	Homo sapiens chromosome 20 open reading frame 54 (C20orf54), mRNA [NM_033409]	NM_033409	3.29
A_24_P116587	*SEZ6L2*	Homo sapiens seizure related 6 homolog (mouse)-like 2 (SEZ6L2), transcript variant 2, mRNA [NM_201575]	NM_201575	3.15
A_23_P155052	*APOL6*	Homo sapiens apolipoprotein L, 6 (APOL6), mRNA [NM_030641]	NM_030641	3.13
A_24_P168398	*ZNF177*	Homo sapiens zinc finger protein 177 (ZNF177), transcript variant 3, mRNA [NM_003451]	NM_003451	3.10
A_23_P74575	*CD1D*	Homo sapiens CD1d molecule (CD1D), mRNA [NM_001766]	NM_001766	3.06
A_23_P251881	*NCR3*	Homo sapiens natural cytotoxicity triggering receptor 3 (NCR3), transcript variant 1, mRNA [NM_147130]	NM_147130	2.99
A_23_P10194	*SEZ6L2*	Homo sapiens seizure related 6 homolog (mouse)-like 2 (SEZ6L2), transcript variant 2, mRNA [NM_201575]	NM_201575	2.98
A_23_P107981	*SULT2B1*	Homo sapiens sulfotransferase family, cytosolic, 2B, member 1 (SULT2B1), transcript variant 1, mRNA	NM_004605	2.97
A_24_P11208	*CD1D*	Homo sapiens CD1d molecule (CD1D), mRNA [NM_001766]	NM_001766	2.78
A_32_P459533	*FCHO1*	Homo sapiens FCH domain only 1 (FCHO1), transcript variant 2, mRNA [NM_015122]	NM_015122	2.69
A_23_P201687	*HES2*	Homo sapiens hairy and enhancer of split 2 (Drosophila), mRNA (cDNA clone IMAGE:4634002), complete cds. [BC012091]	BC012091	2.64
A_23_P201939	*PPM1J*	Homo sapiens protein phosphatase, Mg2+/Mn2+ dependent, 1J (PPM1J), mRNA [NM_005167]	NM_005167	2.62
A_23_P8913	*CA2*	Homo sapiens carbonic anhydrase II (CA2), mRNA [NM_000067]	NM_000067	2.62
A_24_P221154	*ABCD3*	Homo sapiens ATP-binding cassette, sub-family D (ALD), member 3 (ABCD3), transcript variant 1, mRNA	NM_002858	2.61
A_23_P202773	*C11orf20*	Homo sapiens chromosome 11 open reading frame 20 (C11orf20), mRNA [NM_001039496]	NM_001039496	2.59
A_23_P211428	*SMTN*	Homo sapiens smoothelin (SMTN), transcript variant 2, mRNA [NM_134269]	NM_134269	2.59
A_23_P148308	*RBM3*	Homo sapiens RNA binding motif (RNP1, RRM) protein 3 (RBM3), mRNA [NM_006743]	NM_006743	2.58
A_23_P397910	*CBLC*	Homo sapiens Cas-Br-M (murine) ecotropic retroviral transforming sequence c (CBLC), transcript variant 1, mRNA [NM_012116]	NM_012116	2.57
A_23_P400310	*REG4*	Homo sapiens regenerating islet-derived family, member 4 (REG4), transcript variant 2, mRNA [NM_032044]	NM_032044	2.55
A_24_P194881	*SHANK3*	Homo sapiens SH3 and multiple ankyrin repeat domains 3 (SHANK3), mRNA [NM_001080420]	NM_001080420	2.52
A_23_P62768	*TMEM54*	Homo sapiens transmembrane protein 54 (TMEM54), mRNA [NM_033504]	NM_033504	2.48
A_32_P155984		Q40J89_EHRCH (Q40J89) Cation efflux protein, partial (6%) [THC2733597]		2.47
A_24_P237686				2.42
A_24_P342312	*ODZ4*	Homo sapiens odz, odd Oz/ten-m homolog 4 (Drosophila) (ODZ4), mRNA [NM_001098816]	NM_001098816	2.41
A_24_P243633	*DCBLD1*	Homo sapiens discoidin, CUB and LCCL domain containing 1 (DCBLD1), mRNA [NM_173674]	NM_173674	2.39
A_24_P188447	*ELAVL4*	Homo sapiens ELAV (embryonic lethal, abnormal vision, Drosophila)-like 4 (Hu antigen D) (ELAVL4), transcript variant 1, mRNA [NM_021952]	NM_021952	2.38
A_23_P43810	*LTBP1*	Homo sapiens latent transforming growth factor beta binding protein 1 (LTBP1), transcript variant 1, mRNA	NM_206943	2.37
A_23_P154818	*C21orf15*	Homo sapiens mRNA; cDNA DKFZp686M2227 (from clone DKFZp686M2227). [BX648100]	BX648100	2.35
A_32_P24762		he40h05.x1 NCI_CGAP_CML1 Homo sapiens cDNA clone IMAGE:2921529 3' similar to contains Alu repetitive element;contains element MER11 repetitive element ;, mRNA sequence [AW589254]	AW589254	2.35
A_32_P207802				2.34
A_32_P162709		full-length cDNA clone CS0DC002YA18 of Neuroblastoma Cot 25-normalized of Homo sapiens (human).	CR624517	2.33
A_24_P376029	*NAG18*	Homo sapiens NAG18 (NAG18) mRNA, complete cds. [AF210651]	AF210651	2.32
A_24_P371276	*RNF165*	Homo sapiens cDNA FLJ90080 fis, clone HEMBA1004797. [AK074561]	AK074561	2.32
A_23_P324453	*ZNF254*	Homo sapiens zinc finger protein 254 (ZNF254), mRNA [NM_203282]	NM_203282	2.30
A_24_P552450				2.27
A_24_P399490	*KLK10*	Homo sapiens kallikrein-related peptidase 10 (KLK10), transcript variant 1, mRNA [NM_002776]	NM_002776	2.27
A_23_P168828	*KLF10*	Homo sapiens Kruppel-like factor 10 (KLF10), transcript variant 1, mRNA [NM_005655]	NM_005655	2.26
A_23_P76823	*ADSSL1*	Homo sapiens adenylosuccinate synthase like 1 (ADSSL1), transcript variant 1, mRNA [NM_199165]	NM_199165	2.26
A_23_P71415	*WDYHV1*	Homo sapiens WDYHV motif containing 1 (WDYHV1), mRNA [NM_018024]	NM_018024	2.26
A_24_P304154	*AMPD3*	Homo sapiens adenosine monophosphate deaminase 3 (AMPD3), transcript variant 3, mRNA	NM_001025390	2.24
A_23_P170667	*ASPHD1*	Homo sapiens aspartate beta-hydroxylase domain containing 1 (ASPHD1), mRNA [NM_181718]	NM_181718	2.22
A_23_P210811	*CD93*	Homo sapiens CD93 molecule (CD93), mRNA [NM_012072]	NM_012072	2.22
A_23_P136619	*CA13*	Homo sapiens carbonic anhydrase XIII (CA13), mRNA [NM_198584]	NM_198584	2.20
A_23_P69179	*LEPREL1*	Homo sapiens leprecan-like 1 (LEPREL1), transcript variant 1, mRNA [NM_018192]	NM_018192	2.20
A_24_P889103	*SUZ12P*	full-length cDNA clone CS0DC012YL18 of Neuroblastoma Cot 25-normalized of Homo sapiens (human).	CR597846	2.19
A_23_P353125	*FSIP1*	Homo sapiens fibrous sheath interacting protein 1 (FSIP1), mRNA [NM_152597]	NM_152597	2.19
A_24_P828818				2.18
A_23_P433798	*PODNL1*	Homo sapiens podocan-like 1 (PODNL1), transcript variant 1, mRNA [NM_024825]	NM_024825	2.17
A_24_P829934		ALU2_HUMAN (P39189) Alu subfamily SB sequence contamination warning entry, partial (7%) [THC2683069]		2.15
A_23_P7965	*PGC*	Homo sapiens progastricsin (pepsinogen C) (PGC), transcript variant 1, mRNA [NM_002630]	NM_002630	2.15
A_23_P62999	*EXTL1*	Homo sapiens exostoses (multiple)-like 1 (EXTL1), mRNA [NM_004455]	NM_004455	2.14
A_32_P2730	*BCAN*	Homo sapiens brevican, mRNA (cDNA clone IMAGE:3618761), with apparent retained intron. [BC005081]	BC005081	2.11
A_23_P76992	*PGF*	Homo sapiens placental growth factor (PGF), mRNA [NM_002632]	NM_002632	2.10
A_23_P380271	*FLJ23867*	Homo sapiens hypothetical protein FLJ23867 (FLJ23867), non-coding RNA [NR_026900]	NR_026900	2.09
A_24_P936963		CV390614 QV2-HT0577-160500-216-e01 HT0577 Homo sapiens cDNA, mRNA sequence [CV390614]	CV390614	2.08
A_24_P922877	*KLC1*	Homo sapiens kinesin light chain 1 (KLC1), transcript variant 2, mRNA [NM_182923]	NM_182923	2.08
A_23_P121037	*PODXL2*	Homo sapiens podocalyxin-like 2 (PODXL2), mRNA [NM_015720]	NM_015720	2.07
A_23_P208737	*FOXA3*	Homo sapiens forkhead box A3 (FOXA3), mRNA [NM_004497]	NM_004497	2.05
A_24_P148762	*IL1RAP*	Homo sapiens interleukin 1 receptor accessory protein (IL1RAP), transcript variant 1, mRNA [NM_002182]	NM_002182	2.05
A_24_P910703		H.sapiens mRNA for heavy chain variable region, SM1-VH. [X87893]	X87893	2.04
A_24_P324640	*ZNF544*	Homo sapiens zinc finger protein 544 (ZNF544), mRNA [NM_014480]	NM_014480	2.04
A_23_P116286	*AMPD3*	Homo sapiens adenosine monophosphate deaminase 3 (AMPD3), transcript variant 3, mRNA	NM_001025390	2.04
A_23_P59388	*DST*	Homo sapiens dystonin (DST), transcript variant 1e, mRNA [NM_001723]	NM_001723	2.04
A_24_P791040	*FBXW2*	Homo sapiens F-box and WD repeat domain containing 2 (FBXW2), mRNA [NM_012164]	NM_012164	2.03
A_24_P170983	*ESPNL*	Homo sapiens espin-like (ESPNL), mRNA [NM_194312]	NM_194312	2.02
A_24_P354715	*NT5E*	Homo sapiens 5'-nucleotidase, ecto (CD73) (NT5E), mRNA [NM_002526]	NM_002526	2.02
A_23_P170857	*IL1RAP*	Homo sapiens interleukin 1 receptor accessory protein (IL1RAP), transcript variant 1, mRNA [NM_002182]	NM_002182	2.02
A_24_P860248		nq03d05.s1 NCI_CGAP_Lu1 Homo sapiens cDNA clone IMAGE:1142793 3' similar to contains Alu repetitive element;contains element MIR repetitive element ;, mRNA sequence [AA604529]	AA604529	2.02
A_32_P515920	*LOC400573*	Homo sapiens hypothetical gene supported by BC015790; BC041634, mRNA (cDNA clone IMAGE:4865886). [BC041634]	BC041634	2.01
A_24_P923028	*TAT*	Homo sapiens tyrosine aminotransferase, mRNA (cDNA clone IMAGE:4710626), complete cds. [BC020707]	BC020707	-2.00
A_23_P70719	*LAMA2*	Homo sapiens laminin, alpha 2 (LAMA2), transcript variant 1, mRNA [NM_000426]	NM_000426	-2.01
A_32_P10083		Homo sapiens mRNA; cDNA DKFZp547E184 (from clone DKFZp547E184). [AL390170]	AL390170	-2.01
A_32_P37461		Homo sapiens cDNA clone IMAGE:5262677. [BC035106]	BC035106	-2.01
A_32_P51082		AW188368 xj95g10.x1 Soares_NFL_T_GBC_S1 Homo sapiens cDNA clone IMAGE:2665026 3', mRNA sequence [AW188368]	AW188368	-2.01
A_23_P57482		Em:AC005003.4 protein [Source:UniProtKB/TrEMBL;Acc:Q6ICM0] [ENST00000436050]	AK000471	-2.02
A_23_P143514	*C21orf122*	Homo sapiens chromosome 21 open reading frame 122 (C21orf122), non-coding RNA [NR_027292]	NR_027292	-2.02

A_32_P52018	*PHACTR1*	Homo sapiens phosphatase and actin regulator 1 (PHACTR1), mRNA [NM_030948]	NM_030948	-2.02
A_32_P428129	*CDKN1C*	Homo sapiens cyclin-dependent kinase inhibitor 1C (p57, Kip2) (CDKN1C), transcript variant 1, mRNA	NM_000076	-2.02
A_23_P380298	*ProSAPiP1*	Homo sapiens ProSAPiP1 protein (ProSAPiP1), mRNA [NM_014731]	NM_014731	-2.03
A_23_P386254	*NKX3-2*	Homo sapiens NK3 homeobox 2 (NKX3-2), mRNA [NM_001189]	NM_001189	-2.03
A_32_P76255		UI-E-EJ1-ajr-d-04-0-UI.r1 UI-E-EJ1 Homo sapiens cDNA clone UI-E-EJ1-ajr-d-04-0-UI 5', mRNA sequence	BQ186660	-2.03
A_24_P937265		AW975013 EST387118 MAGE resequences, MAGN Homo sapiens cDNA, mRNA sequence [AW975013]	AW975013	-2.04
A_23_P136721		Human endogenous retrovirus H protease/integrase-derived ORF1, ORF2, and putative envelope protein mRNA, complete cds. [U88896]	U88896	-2.04
A_24_P910842				-2.04
A_24_P620951	*LOC219731*	Homo sapiens cDNA FLJ32141 fis, clone PLACE5000067. [AK056703]	AK056703	-2.05
A_24_P763407		Q9GKC6_THEGE (Q9GKC6) Testis-specific protein (Fragment), partial (79%) [THC2784707]		-2.06
A_23_P342727	*STARD13*	Homo sapiens StAR-related lipid transfer (START) domain containing 13 (STARD13), transcript variant alpha, mRNA [NM_178006]	NM_178006	-2.06
A_23_P120103	*KCNS3*	Homo sapiens potassium voltage-gated channel, delayed-rectifier, subfamily S, member 3 (KCNS3), mRNA [NM_002252]	NM_002252	-2.07
A_23_P413051	*NOXO1*	Homo sapiens NADPH oxidase organizer 1 (NOXO1), transcript variant a, mRNA [NM_144603]	NM_144603	-2.07
A_32_P3131		Homo sapiens cDNA FLJ14181 fis, clone NT2RP2004300. [AK024243]	AK024243	-2.07
A_23_P391344	*RASGEF1A*	Homo sapiens RasGEF domain family, member 1A, mRNA (cDNA clone MGC:26821 IMAGE:4814750), complete cds. [BC022548]	BC022548	-2.07
A_24_P306594	*C1orf167*	Homo sapiens mRNA; cDNA DKFZp434F1313 (from clone DKFZp434F1313). [AL834308]	AL834308	-2.07
A_32_P62469		BQ307884 MR0-BT4501-250601-102-c01 BT4501 Homo sapiens cDNA, mRNA sequence [BQ307884]	BQ307884	-2.08
A_32_P223616				-2.08
A_32_P56463		BX101252 NCI_CGAP_Lu24 Homo sapiens cDNA clone IMAGp998I115625, mRNA sequence [BX101252]	BX101252	-2.08
A_32_P10383				-2.09
A_23_P64873	*DCN*	Homo sapiens decorin (DCN), transcript variant A1, mRNA [NM_001920]	NM_001920	-2.09
A_32_P185361		Homo sapiens mRNA full length insert cDNA clone EUROIMAGE 122871. [AL109784]	AL109784	-2.09
A_24_P620066		AA905316 ok01a08.s1 Soares_NFL_T_GBC_S1 Homo sapiens cDNA clone IMAGE:1506518 3', mRNA sequence [AA905316]	AA905316	-2.09
A_32_P4934				-2.09
A_23_P11543	*FUCA1*	Homo sapiens fucosidase, alpha-L- 1, tissue (FUCA1), mRNA [NM_000147]	NM_000147	-2.09
A_23_P106280	*FLJ10038*	Homo sapiens hypothetical protein FLJ10038 (FLJ10038), non-coding RNA [NR_026891]	NR_026891	-2.10
A_23_P204133	*GALNT6*	Homo sapiens UDP-N-acetyl-alpha-D-galactosamine:polypeptide N-acetylgalactosaminyltransferase 6 (GalNAc-T6) (GALNT6), mRNA [NM_007210]	NM_007210	-2.11
A_32_P834726		Homo sapiens cDNA FLJ31117 fis, clone IMR322000609. [AK055679]	AK055679	-2.11
A_32_P31757		BX094773 NCI_CGAP_Lu5 Homo sapiens cDNA clone IMAGp998F243582, mRNA sequence [BX094773]	BX094773	-2.11
A_32_P69639				-2.11
A_23_P203053	*NCAM1*	Homo sapiens neural cell adhesion molecule 1 (NCAM1), transcript variant 3, mRNA [NM_001076682]	NM_001076682	-2.12
A_32_P46351				-2.13
A_24_P340112	*PDK3*	Homo sapiens pyruvate dehydrogenase kinase, isozyme 3 (PDK3), nuclear gene encoding mitochondrial protein, transcript variant 1, mRNA [NM_001142386]	NM_001142386	-2.14
A_23_P113056				-2.15
A_32_P119878				-2.15
A_32_P3932		Q4RS58_TETNG (Q4RS58) Chromosome 13 SCAF15000, whole genome shotgun sequence, partial (11%) [THC2692456]		-2.15
A_32_P86739	*C10orf114*	Homo sapiens chromosome 10 open reading frame 114 (C10orf114), mRNA [NM_001010911]	NM_001010911	-2.15
A_32_P790284	*KATNAL2*	Homo sapiens katanin p60 subunit A-like 2 (KATNAL2), mRNA [NM_031303]	NM_031303	-2.16
A_23_P28530	*MFSD6*	Homo sapiens major facilitator superfamily domain containing 6 (MFSD6), mRNA [NM_017694]	NM_017694	-2.16
A_32_P51119	*STOX1*	Homo sapiens storkhead box 1 (STOX1), transcript variant 1, mRNA [NM_152709]	NM_152709	-2.17
A_24_P911860	*DUSP19*	Homo sapiens dual specificity phosphatase 19 (DUSP19), transcript variant 1, mRNA [NM_080876]	NM_080876	-2.18
A_24_P603453				-2.19
A_23_P130974	*KIAA1683*	Homo sapiens KIAA1683 (KIAA1683), transcript variant 2, mRNA [NM_025249]	NM_025249	-2.20
A_23_P162171	*MCAM*	Homo sapiens melanoma cell adhesion molecule (MCAM), mRNA [NM_006500]	NM_006500	-2.20
A_23_P52121	*PDZK1*	Homo sapiens PDZ domain containing 1 (PDZK1), mRNA [NM_002614]	NM_002614	-2.20
A_23_P372308	*RGMA*	Homo sapiens RGM domain family, member A (RGMA), transcript variant 4, mRNA [NM_020211]	NM_020211	-2.20
A_23_P144622	*GNPDA1*	Homo sapiens glucosamine-6-phosphate deaminase 1 (GNPDA1), mRNA [NM_005471]	NM_005471	-2.20
A_23_P259621	*LAT2*	Homo sapiens linker for activation of T cells family, member 2 (LAT2), transcript variant 1, mRNA	NM_032464	-2.21
A_24_P669822				-2.21
A_24_P393051		Homo sapiens cDNA FLJ39732 fis, clone SMINT2015810. [AK097051]	AK097051	-2.21
A_32_P205478		AGENCOURT_8859642 NIH_MGC_71 Homo sapiens cDNA clone IMAGE:6473897 5', mRNA sequence	BQ948285	-2.22
A_24_P102539	*KRIT1*	Homo sapiens KRIT1, ankyrin repeat containing (KRIT1), transcript variant 5, mRNA [NM_001013406]	NM_001013406	-2.22
A_32_P77252				-2.23
A_23_P44132	*FASN*	Homo sapiens fatty acid synthase (FASN), mRNA [NM_004104]	NM_004104	-2.24
A_23_P55828	*CCL25*	Homo sapiens chemokine (C-C motif) ligand 25 (CCL25), mRNA [NM_005624]	NM_005624	-2.24
A_23_P75024	*MSMB*	Homo sapiens microseminoprotein, beta- (MSMB), transcript variant PSP94, mRNA [NM_002443]	NM_002443	-2.24
A_32_P171061	*ASCL2*	Homo sapiens achaete-scute complex homolog 2 (Drosophila) (ASCL2), mRNA [NM_005170]	NM_005170	-2.25
A_23_P311869	*ST3GAL5*	Homo sapiens ST3 beta-galactoside alpha-2,3-sialyltransferase 5 (ST3GAL5), transcript variant 1, mRNA	NM_003896	-2.25
A_24_P282383	*MYH7*	Homo sapiens myosin, heavy chain 7, cardiac muscle, beta (MYH7), mRNA [NM_000257]	NM_000257	-2.26
A_32_P139815		BX094072 Soares fetal liver spleen 1NFLS Homo sapiens cDNA clone IMAGp998J18385, mRNA sequence	BX094072	-2.26
A_23_P145935	*EPHB6*	Homo sapiens EPH receptor B6 (EPHB6), mRNA [NM_004445]	NM_004445	-2.27
A_23_P251002				-2.28
A_32_P179615				-2.28
A_23_P126266	*HLX*	Homo sapiens H2.0-like homeobox (HLX), mRNA [NM_021958]	NM_021958	-2.28
A_32_P213948				-2.30
A_23_P6722	*SPATA16*	Homo sapiens spermatogenesis associated 16 (SPATA16), mRNA [NM_031955]	NM_031955	-2.30
A_32_P176911	*C14orf72*	Homo sapiens chromosome 14 open reading frame 72 (C14orf72), non-coding RNA [NR_026774]	NR_026774	-2.30
A_23_P420431	*XKR3*	Homo sapiens XK, Kell blood group complex subunit-related family, member 3 (XKR3), mRNA [NM_175878]	NM_175878	-2.31
A_32_P11325				-2.31
A_24_P60887	*ANGPT1*	Homo sapiens angiopoietin 1 (ANGPT1), mRNA [NM_001146]	NM_001146	-2.33
A_23_P135164	*UAP1L1*	Homo sapiens UDP-N-acteylglucosamine pyrophosphorylase 1-like 1 (UAP1L1), mRNA [NM_207309]	NM_207309	-2.33
A_23_P134237	*RARRES2*	Homo sapiens retinoic acid receptor responder (tazarotene induced) 2 (RARRES2), mRNA [NM_002889]	NM_002889	-2.34
A_23_P405815	*PYGM*	Homo sapiens phosphorylase, glycogen, muscle (PYGM), transcript variant 1, mRNA [NM_005609]	NM_005609	-2.34
A_24_P146683	*MSMB*	Homo sapiens microseminoprotein, beta- (MSMB), transcript variant PSP94, mRNA [NM_002443]	NM_002443	-2.35
A_23_P101374	*CYP2S1*	Homo sapiens cytochrome P450, family 2, subfamily S, polypeptide 1 (CYP2S1), mRNA [NM_030622]	NM_030622	-2.36
A_24_P714984		Q8N329_HUMAN (Q8N329) C3orf64 protein, partial (18%) [THC2618237]		-2.37
A_32_P71032				-2.37
A_23_P66732	*GSG2*	Homo sapiens germ cell associated 2 (haspin) (GSG2), mRNA [NM_031965]	NM_031965	-2.39
A_32_P101860				-2.40
A_32_P23626				-2.41
A_32_P115349				-2.42
A_24_P221007	*ANKRD30B*	Homo sapiens ankyrin repeat domain 30B (ANKRD30B), mRNA [NM_001145029]	NM_001145029	-2.45
A_32_P118675		Q3AUE7_CHLCH (Q3AUE7) Glucosamine-fructose-6-phosphate aminotransferase, isomerising , partial (4%) [THC2660784]		-2.45
A_24_P920227				-2.46
A_23_P380240	*CEACAM8*	Homo sapiens carcinoembryonic antigen-related cell adhesion molecule 8 (CEACAM8), mRNA [NM_001816]	NM_001816	-2.47
A_24_P68908	*LOC344887*	Homo sapiens mRNA; cDNA DKFZp686B14224 (from clone DKFZp686B14224). [BX640843]	BX640843	-2.48
A_32_P57486		Q3BCG3_9ARAC (Q3BCG3) Tubuliform spidroin (Fragment), partial (5%) [THC2721785]		-2.48
A_23_P40611	*TCN2*	Homo sapiens transcobalamin II; macrocytic anemia (TCN2), mRNA [NM_000355]	NM_000355	-2.49
A_32_P179859	*LOC728705*	Homo sapiens cDNA FLJ31150 fis, clone IMR322001534. [AK055712]	AK055712	-2.49
A_23_P431569	*LOC100049716*	Homo sapiens hypothetical protein LOC100049716, mRNA (cDNA clone IMAGE:3621928), partial cds.	BC033133	-2.49
A_23_P250951	*SLC26A2*	Homo sapiens solute carrier family 26 (sulfate transporter), member 2 (SLC26A2), mRNA [NM_000112]	NM_000112	-2.51
A_23_P109171	*BFSP1*	Homo sapiens beaded filament structural protein 1, filensin (BFSP1), transcript variant 1, mRNA [NM_001195]	NM_001195	-2.53
A_32_P14582	*C3P1*	Homo sapiens complement component 3 precursor pseudogene (C3P1), non-coding RNA [NR_027300]	NR_027300	-2.53
A_24_P316381		Homo sapiens KIAA1267, mRNA (cDNA clone IMAGE:3948445), **** WARNING: chimeric clone ****.	BC006271	-2.54

A_23_P257043	*GEM*	Homo sapiens GTP binding protein overexpressed in skeletal muscle (GEM), transcript variant 1, mRNA	NM_005261	-2.55
A_23_P356004	*KCNIP3*	Homo sapiens Kv channel interacting protein 3, calsenilin (KCNIP3), transcript variant 1, mRNA [NM_013434]	NM_013434	-2.55
A_23_P19529	*MLN*	Homo sapiens motilin (MLN), transcript variant 2, mRNA [NM_001040109]	NM_001040109	-2.58
A_24_P758256				-2.59
A_23_P156890	*TCF21*	Homo sapiens transcription factor 21 (TCF21), transcript variant 2, mRNA [NM_003206]	NM_003206	-2.59
A_23_P421011	*KAZALD1*	Homo sapiens Kazal-type serine peptidase inhibitor domain 1 (KAZALD1), mRNA [NM_030929]	NM_030929	-2.59
A_23_P51213	*MYOM3*	Homo sapiens myomesin family, member 3 (MYOM3), mRNA [NM_152372]	NM_152372	-2.61
A_32_P17484		Homo sapiens mRNA; cDNA DKFZp686J0581 (from clone DKFZp686J0581). [BX647685]	BX647685	-2.61
A_23_P40108	*COL9A3*	Homo sapiens collagen, type IX, alpha 3 (COL9A3), mRNA [NM_001853]	NM_001853	-2.61
A_24_P76675	*MFAP3L*	Homo sapiens microfibrillar-associated protein 3-like (MFAP3L), transcript variant 1, mRNA [NM_021647]	NM_021647	-2.62
A_32_P182045		full-length cDNA clone CS0DF003YI18 of Fetal brain of Homo sapiens (human). [CR598370]	CR598370	-2.63
A_23_P102731	*SMOX*	Homo sapiens spermine oxidase (SMOX), transcript variant 1, mRNA [NM_175839]	NM_175839	-2.64
A_32_P207767		Putative uncharacterized protein ENSP00000381813 [Source:UniProtKB/TrEMBL;Acc:A8MZ04]		-2.65
A_23_P55682	*ZSCAN18*	Homo sapiens zinc finger and SCAN domain containing 18 (ZSCAN18), transcript variant 3, mRNA	NM_023926	-2.66
A_23_P154358	*PROM2*	Homo sapiens prominin 2 (PROM2), transcript variant 3, mRNA [NM_144707]	NM_144707	-2.67
A_32_P24832	*OLFML3*	Homo sapiens olfactomedin-like 3 (OLFML3), mRNA [NM_020190]	NM_020190	-2.68
A_24_P914573	*SULT1A3*	Homo sapiens cDNA FLJ37450 fis, clone BRAWH2010354, highly similar to Monoamine-sulfating phenol sulfotransferase (EC 2.8.2.1). [AK094769]	AK094769	-2.68
A_32_P197340	*LOC285141*	full-length cDNA clone CS0DI052YH08 of Placenta Cot 25-normalized of Homo sapiens (human). [CR596204]	CR596204	-2.69
A_32_P15958	*C21orf121*	Homo sapiens chromosome 21 open reading frame 121 (C21orf121), non-coding RNA [NR_027273]	NR_027273	-2.75
A_24_P885873	*LOC654433*	Homo sapiens cDNA FLJ26765 fis, clone PRS02774. [AK130275]	AK130275	-2.75
A_23_P153540	*FAM83E*	Homo sapiens family with sequence similarity 83, member E (FAM83E), mRNA [NM_017708]	NM_017708	-2.77
A_23_P36905	*MLNR*	Homo sapiens motilin receptor (MLNR), mRNA [NM_001507]	NM_001507	-2.80
A_23_P365267	*SNED1*	Homo sapiens sushi, nidogen and EGF-like domains 1 (SNED1), mRNA [NM_001080437]	NM_001080437	-2.80
A_23_P160559	*ECM1*	Homo sapiens extracellular matrix protein 1 (ECM1), transcript variant 1, mRNA [NM_004425]	NM_004425	-2.81
A_23_P207221	*SLC47A1*	Homo sapiens solute carrier family 47, member 1 (SLC47A1), mRNA [NM_018242]	NM_018242	-2.82
A_32_P102300		Q333X5_SPAJD (Q333X5) Heparanase, partial (3%) [THC2687538]		-2.82
A_23_P151297	*TENC1*	Homo sapiens tensin like C1 domain containing phosphatase (tensin 2) (TENC1), transcript variant 1, mRNA [NM_015319]	NM_015319	-2.84
A_23_P137665	*CHI3L1*	Homo sapiens chitinase 3-like 1 (cartilage glycoprotein-39) (CHI3L1), mRNA [NM_001276]	NM_001276	-2.84
A_23_P397208	*GSTM2*	Homo sapiens glutathione S-transferase mu 2 (muscle) (GSTM2), transcript variant 1, mRNA [NM_000848]	NM_000848	-2.84
A_23_P421401	*PDGFRB*	Homo sapiens platelet-derived growth factor receptor, beta polypeptide (PDGFRB), mRNA [NM_002609]	NM_002609	-2.85
A_32_P735429		RNSAP seum amyloid P component {Rattus norvegicus} (exp=-1; wgp=0; cg=0), partial (30%) [THC2654992]		-2.86
A_23_P148852		Homo sapiens neuronal thread protein AD7c-NTP mRNA, complete cds. [AF010144]	AF010144	-2.90
A_24_P927976	*KATNAL2*	Homo sapiens mRNA; cDNA DKFZp667C165 (from clone DKFZp667C165). [AL512748]	AL512748	-2.90
A_24_P231004	*TIMELESS*	Homo sapiens timeless homolog (Drosophila) (TIMELESS), mRNA [NM_003920]	NM_003920	-2.90
A_23_P216023	*ANGPT1*	Homo sapiens angiopoietin 1 (ANGPT1), mRNA [NM_001146]	NM_001146	-2.91
A_23_P143817	*MYLK*	Homo sapiens myosin light chain kinase (MYLK), transcript variant 1, mRNA [NM_053025]	NM_053025	-2.94
A_23_P141173	*MPO*	Homo sapiens myeloperoxidase (MPO), nuclear gene encoding mitochondrial protein, mRNA [NM_000250]	NM_000250	-2.95
A_23_P344125	*ISM2*	Homo sapiens isthmin 2 homolog (zebrafish) (ISM2), transcript variant 1, mRNA [NM_199296]	NM_199296	-2.95
A_32_P203029	*FAM9B*	Homo sapiens family with sequence similarity 9, member B (FAM9B), mRNA [NM_205849]	NM_205849	-2.96
A_23_P140821	*PARD6A*	Homo sapiens par-6 partitioning defective 6 homolog alpha (C. elegans) (PARD6A), transcript variant 1, mRNA [NM_016948]	NM_016948	-3.02
A_24_P119545	*ITPKB*	Homo sapiens inositol 1,4,5-trisphosphate 3-kinase B, mRNA (cDNA clone MGC:23636 IMAGE:4862726), complete cds. [BC015009]	BC015009	-3.10
A_23_P128728	*ARG2*	Homo sapiens arginase, type II (ARG2), nuclear gene encoding mitochondrial protein, mRNA [NM_001172]	NM_001172	-3.15
A_23_P136573	*ST3GAL5*	Homo sapiens ST3 beta-galactoside alpha-2,3-sialyltransferase 5 (ST3GAL5), transcript variant 1, mRNA	NM_003896	-3.24
A_24_P185909	*LONRF3*	Homo sapiens LON peptidase N-terminal domain and ring finger 3 (LONRF3), transcript variant 1, mRNA [NM_001031855]	NM_001031855	-3.25
A_23_P114713	*CYP4B1*	Homo sapiens cytochrome P450, family 4, subfamily B, polypeptide 1 (CYP4B1), transcript variant 2, mRNA [NM_000779]	NM_000779	-3.27
A_24_P153547	*RPS6KL1*	Homo sapiens ribosomal protein S6 kinase-like 1 (RPS6KL1), mRNA [NM_031464]	NM_031464	-3.31
A_23_P134734	*GOLSYN*	Homo sapiens Golgi-localized protein (GOLSYN), transcript variant 9, mRNA [NM_017786]	NM_017786	-3.41
A_23_P83134	*GAS1*	Homo sapiens growth arrest-specific 1 (GAS1), mRNA [NM_002048]	NM_002048	-3.44
A_32_P64716		Homo sapiens cDNA clone IMAGE:5262677. [BC035106]	BC035106	-3.45
A_32_P135634		Q2RZ67_SALRD (Q2RZ67) Glycosyl transferase, group 1 family protein , partial (6%) [THC2643352]		-3.52
A_23_P118203	*ZG16B*	Homo sapiens zymogen granule protein 16 homolog B (rat) (ZG16B), mRNA [NM_145252]	NM_145252	-3.53
A_32_P232198				-3.55
A_23_P140748	*NDRG4*	Homo sapiens NDRG family member 4 (NDRG4), transcript variant 3, mRNA [NM_022910]	NM_022910	-3.56
A_23_P111171	*B3GALT4*	Homo sapiens UDP-Gal:betaGlcNAc beta 1,3-galactosyltransferase, polypeptide 4 (B3GALT4), mRNA	NM_003782	-3.57
A_23_P404481	*S1PR1*	Homo sapiens sphingosine-1-phosphate receptor 1 (S1PR1), mRNA [NM_001400]	NM_001400	-3.65
A_23_P94800	*S100A4*	Homo sapiens S100 calcium binding protein A4 (S100A4), transcript variant 1, mRNA [NM_002961]	NM_002961	-3.66
A_23_P256033	*EEF1A2*	Homo sapiens eukaryotic translation elongation factor 1 alpha 2 (EEF1A2), mRNA [NM_001958]	NM_001958	-3.66
A_23_P17914	*PNPLA3*	Homo sapiens patatin-like phospholipase domain containing 3 (PNPLA3), mRNA [NM_025225]	NM_025225	-3.72
A_23_P420326	*FNDC5*	Homo sapiens fibronectin type III domain containing 5 (FNDC5), transcript variant 2, mRNA [NM_153756]	NM_153756	-3.74
A_23_P422071	*B3GALT4*	Homo sapiens UDP-Gal:betaGlcNAc beta 1,3-galactosyltransferase, polypeptide 4 (B3GALT4), mRNA	NM_003782	-3.74
A_23_P93122	*MEP1A*	Homo sapiens meprin A, alpha (PABA peptide hydrolase) (MEP1A), mRNA [NM_005588]	NM_005588	-3.75
A_23_P85008	*MAOB*	Homo sapiens monoamine oxidase B (MAOB), nuclear gene encoding mitochondrial protein, mRNA	NM_000898	-3.79
A_32_P207169	*C1orf133*	Homo sapiens chromosome 1 open reading frame 133 (C1orf133), non-coding RNA [NR_024337]	NR_024337	-3.82
A_24_P3016		602689867F1 NIH_MGC_97 Homo sapiens cDNA clone IMAGE:4822264 5', mRNA sequence [BG719660]	BG719660	-3.87
A_23_P125505	*PPEF1*	Homo sapiens protein phosphatase, EF-hand calcium binding domain 1 (PPEF1), transcript variant 1, mRNA [NM_006240]	NM_006240	-3.94
A_32_P217643		full-length cDNA clone CS0DI068YD14 of Placenta Cot 25-normalized of Homo sapiens (human). [CR624623]	CR624623	-4.01
A_23_P112289	*TMOD1*	Homo sapiens tropomodulin 1 (TMOD1), transcript variant 1, mRNA [NM_003275]	NM_003275	-4.02
A_32_P181297	*ST7OT1*	Homo sapiens ST7 overlapping transcript 1 (non-protein coding) (ST7OT1), antisense RNA [NR_002330]	NR_002330	-4.03
A_23_P86653	*SRGN*	Homo sapiens serglycin (SRGN), mRNA [NM_002727]	NM_002727	-4.05
A_24_P321525	*RERG*	Homo sapiens RAS-like, estrogen-regulated, growth inhibitor (RERG), mRNA [NM_032918]	NM_032918	-4.08
A_23_P102634	*WFDC6*	Homo sapiens WAP four-disulfide core domain 6 (WFDC6), mRNA [NM_080827]	NM_080827	-4.24
A_23_P56197	*CRLF1*	Homo sapiens cytokine receptor-like factor 1 (CRLF1), mRNA [NM_004750]	NM_004750	-4.24
A_24_P402825	*CACNA2D3*	Homo sapiens calcium channel, voltage-dependent, alpha 2/delta subunit 3 (CACNA2D3), mRNA	NM_018398	-4.34
A_24_P142503	*SLC47A1*	Homo sapiens solute carrier family 47, member 1 (SLC47A1), mRNA [NM_018242]	NM_018242	-4.42
A_23_P83838	*CA8*	Homo sapiens carbonic anhydrase VIII (CA8), mRNA [NM_004056]	NM_004056	-4.42
A_23_P421306	*SYT12*	Homo sapiens synaptotagmin XII (SYT12), mRNA [NM_177963]	NM_177963	-4.53
A_24_P171043	*DKFZP547L112*	Homo sapiens mRNA; cDNA DKFZp547L112 (from clone DKFZp547L112). [AL512723]	AL512723	-4.57
A_32_P185682		full-length cDNA clone CS0DJ002YF02 of T cells (Jurkat cell line) Cot 10-normalized of Homo sapiens (human). [CR613361]	CR613361	-4.62
A_23_P128362	*MYBPC1*	Homo sapiens myosin binding protein C, slow type (MYBPC1), transcript variant 2, mRNA [NM_206819]	NM_206819	-4.64
A_24_P302172	*PTGFR*	Homo sapiens prostaglandin F receptor (FP) (PTGFR), transcript variant 2, mRNA [NM_001039585]	NM_001039585	-4.64
A_24_P350546	*LOC646976*	Homo sapiens cDNA FLJ38763 fis, clone KIDNE2014119. [AK096082]	AK096082	-4.69
A_23_P114466	*TBL1Y*	Homo sapiens transducin (beta)-like 1, Y-linked (TBL1Y), transcript variant 1, mRNA [NM_033284]	NM_033284	-4.75
A_23_P121926	*SEPP1*	Homo sapiens selenoprotein P, plasma, 1 (SEPP1), transcript variant 1, mRNA [NM_005410]	NM_005410	-4.77
A_23_P85015	*MAOB*	Homo sapiens monoamine oxidase B (MAOB), nuclear gene encoding mitochondrial protein, mRNA	NM_000898	-4.81
A_23_P145644	*DDC*	Homo sapiens dopa decarboxylase (aromatic L-amino acid decarboxylase) (DDC), transcript variant 2, mRNA [NM_000790]	NM_000790	-5.03
A_23_P111583	*CD36*	Homo sapiens CD36 molecule (thrombospondin receptor) (CD36), transcript variant 2, mRNA	NM_001001547	-5.06
A_23_P147166	*CACNA2D3*	Homo sapiens mRNA for calcium channel alpha2-delta3 subunit. [AJ272268]	AJ272268	-5.27
A_23_P253791	*CAMP*	Homo sapiens cathelicidin antimicrobial peptide (CAMP), mRNA [NM_004345]	NM_004345	-5.34
A_23_P81158	*ADH1C*	Homo sapiens alcohol dehydrogenase 1C (class I), gamma polypeptide (ADH1C), mRNA [NM_000669]	NM_000669	-5.70
A_24_P925505	*CD36*	CD36=collagen type I/thrombospondin receptor {one exon} [human, mRNA Partial, 369 nt]. [S67044]	S67044	-5.99
A_23_P330611	*WIPF1*	Homo sapiens WAS/WASL interacting protein family, member 1 (WIPF1), transcript variant 2, mRNA	NM_001077269	-6.27
A_23_P5131	*ISYNA1*	Homo sapiens inositol-3-phosphate synthase 1 (ISYNA1), transcript variant 1, mRNA [NM_016368]	NM_016368	-6.72
A_23_P63209	*HSD11B1*	Homo sapiens hydroxysteroid (11-beta) dehydrogenase 1 (HSD11B1), transcript variant 2, mRNA	NM_181755	-6.79
A_23_P148879	*ATP1A2*	Homo sapiens ATPase, Na+/K+ transporting, alpha 2 (+) polypeptide (ATP1A2), mRNA [NM_000702]	NM_000702	-7.08

A_23_P102611	*WISP2*	Homo sapiens WNT1 inducible signaling pathway protein 2 (WISP2), mRNA [NM_003881]	NM_003881	-8.10
A_23_P69030	*COL8A1*	Homo sapiens collagen, type VIII, alpha 1 (COL8A1), transcript variant 1, mRNA [NM_001850]	NM_001850	-8.59
A_24_P291658	*ADH1A*	Homo sapiens alcohol dehydrogenase 1A (class I), alpha polypeptide (ADH1A), mRNA [NM_000667]	NM_000667	-8.71
A_24_P418744				-9.00
A_24_P139152	*COL8A1*	collagen, type VIII, alpha 1 [Source:HGNC Symbol;Acc:2215] [ENST00000261037]	AL359062	-9.67
A_23_P40415	*ADAMTS5*	Homo sapiens ADAM metallopeptidase with thrombospondin type 1 motif, 5 (ADAMTS5), mRNA [NM_007038]	NM_007038	-9.97
A_23_P58359	*ADH1A*	Homo sapiens alcohol dehydrogenase 1A (class I), alpha polypeptide (ADH1A), mRNA [NM_000667]	NM_000667	-10.62
A_23_P34597	*CDA*	Homo sapiens cytidine deaminase (CDA), mRNA [NM_001785]	NM_001785	-11.00
A_23_P43164	*SULF1*	Homo sapiens sulfatase 1 (SULF1), transcript variant 3, mRNA [NM_015170]	NM_015170	-11.64
