# Supplementary material for: Loss of the BRCA1-Interacting Helicase BRIP1 Results in Abnormal Mammary Acinar Morphogenesis
Source: PLoS One. 2013 Sep 6;8(9):e74013. doi: 10.1371/journal.pone.0074013 (PMC3765252; doi:10.1371/journal.pone.0074013)
Supplement: Table S3 — Up- and down-regulated genes (≥2-fold, P<0.05) in 3D culture of BRIP1-knockdown cells compared with control cells at day 12. (PDF) [file pone.0074013.s005.pdf]

Table S3. Up- and down-regulated genes (≥ 2-fold, $P < 0.05$) in 3D culture of *BRIP1*-knockdown cells compared with control cells at day 12

ProbeName	GeneSymbol	Description	GenbankAccession	Fold change
A_23_P26854	*RICH2*	Homo sapiens Rho-type GTPase-activating protein RICH2 (RICH2), mRNA [NM_014859]	NM_014859	44.26
A_23_P257003	*PCSK5*	Homo sapiens proprotein convertase subtilisin/kexin type 5 (PCSK5), mRNA [NM_006200]	NM_006200	22.72
A_23_P259741	*SATB1*	Homo sapiens SATB homeobox 1 (SATB1), transcript variant 1, mRNA [NM_002971]	NM_002971	9.05
A_23_P157926	*LINGO2*	Homo sapiens leucine rich repeat and Ig domain containing 2 (LINGO2), mRNA [NM_152570]	NM_152570	7.39
A_23_P169437	*LCN2*	Homo sapiens lipocalin 2 (LCN2), mRNA [NM_005564]	NM_005564	5.73
A_23_P209116	*CYP4F3*	Homo sapiens mRNA for leukotriene B4 omega-hydroxylase, complete cds. [AB002454]	AB002454	5.44
A_24_P7642	*FABP5*	Homo sapiens fatty acid binding protein 5 (psoriasis-associated) (FABP5), mRNA [NM_001444]	NM_001444	5.03
A_24_P11208	*CD1D*	Homo sapiens CD1d molecule (CD1D), mRNA [NM_001766]	NM_001766	4.88
A_23_P50638	*LRG1*	Homo sapiens leucine-rich alpha-2-glycoprotein 1 (LRG1), mRNA [NM_052972]	NM_052972	4.85
A_32_P139505	*RP1-21O18.1*	Homo sapiens kazrin (KAZ), transcript variant A, mRNA [NM_015209]	NM_015209	4.58
A_32_P25065				4.51
A_23_P17065	*CCL20*	Homo sapiens chemokine (C-C motif) ligand 20 (CCL20), transcript variant 1, mRNA [NM_004591]	NM_004591	4.27
A_24_P887857				3.67
A_24_P247587	*LOC348751*	Homo sapiens hypothetical protein LOC348751, mRNA (cDNA clone IMAGE:5311172). [BC039445]	BC039445	3.42
A_23_P87238	*SAA4*	Homo sapiens serum amyloid A4, constitutive (SAA4), mRNA [NM_006512]	NM_006512	3.40
A_24_P384422				3.26
A_32_P41996		AI500335 tm95e03.x1 NCI_CGAP_Brn25 Homo sapiens cDNA clone IMAGE:2165884 3', mRNA sequence	AI500335	3.23
A_24_P882732		Putative uncharacterized protein ENSP00000319235 [Source:UniProtKB/TrEMBL;Acc:C9JH30]	DR007925	3.19
A_24_P57977	*SRCIN1*	Homo sapiens SRC kinase signaling inhibitor 1 (SRCIN1), mRNA [NM_025248]	NM_025248	3.16
A_32_P459533	*FCHO1*	Homo sapiens FCH domain only 1 (FCHO1), transcript variant 2, mRNA [NM_015122]	NM_015122	3.11
A_24_P161813	*LOC441666*	Homo sapiens zinc finger protein 91 pseudogene (LOC441666), non-coding RNA [NR_024380]	NR_024380	3.04
A_32_P107208	*C1orf106*	Homo sapiens chromosome 1 open reading frame 106 (C1orf106), transcript variant 1, mRNA [NM_018265]	NM_018265	3.01
A_23_P167509	*CYFIP2*	Homo sapiens cytoplasmic FMR1 interacting protein 2 (CYFIP2), transcript variant 2, mRNA [NM_001037332]	NM_001037332	2.98
A_24_P324424				2.95
A_32_P139123		HMGA2_HUMAN (P52926) High mobility group protein HMGI-C (High mobility group AT-hook protein 2), partial (61%) [THC2560068]		2.89
A_23_P318881	*SIAE*	Homo sapiens sialic acid acetylesterase (SIAE), mRNA [NM_170601]	NM_170601	2.88
A_23_P205913	*SLC24A1*	Homo sapiens solute carrier family 24 (sodium/potassium/calcium exchanger), member 1 (SLC24A1), mRNA [NM_004727]	NM_004727	2.78
A_24_P115651	*ENKUR*	Homo sapiens enkurin, TRPC channel interacting protein (ENKUR), mRNA [NM_145010]	NM_145010	2.77
A_23_P66525	*HS3ST3A1*	Homo sapiens heparan sulfate (glucosamine) 3-O-sulfotransferase 3A1 (HS3ST3A1), mRNA [NM_006042]	NM_006042	2.75
A_23_P8913	*CA2*	Homo sapiens carbonic anhydrase II (CA2), mRNA [NM_000067]	NM_000067	2.74
A_24_P153035		Putative uncharacterized protein ENSP00000319235 [Source:UniProtKB/TrEMBL;Acc:C9JH30]	DR007925	2.72
A_24_P384200				2.67
A_23_P386320	*MFI2*	Homo sapiens antigen p97 (melanoma associated) identified by monoclonal antibodies 133.2 and 96.5 (MFI2), transcript variant 2, mRNA [NM_033316]	NM_033316	2.65
A_24_P383802				2.63
A_24_P384029	*LOC100130171*	PREDICTED: Homo sapiens similar to hematological and neurological expressed 1 (LOC100130171), miscRNA [XR_038676]	XR_038676	2.62
A_24_P278552	*MPZL2*	Homo sapiens myelin protein zero-like 2 (MPZL2), transcript variant 1, mRNA [NM_005797]	NM_005797	2.60
A_23_P4335	*KRT14*	Homo sapiens keratin 14 (KRT14), mRNA [NM_000526]	NM_000526	2.59
A_23_P119936	*REG3A*	Homo sapiens regenerating islet-derived 3 alpha (REG3A), transcript variant 2, mRNA [NM_138938]	NM_138938	2.58
A_24_P238896				2.54
A_24_P772103	*PITPNC1*	Homo sapiens cDNA FLJ37405 fis, clone BRAMY2028269. [AK094724]	AK094724	2.53
A_24_P265346	*KRT14*	Homo sapiens keratin 14 (KRT14), mRNA [NM_000526]	NM_000526	2.52
A_24_P743806	*ZNF618*	Homo sapiens zinc finger protein 618 (ZNF618), mRNA [NM_133374]	NM_133374	2.50
A_23_P50710	*CYP4F2*	Homo sapiens cytochrome P450, family 4, subfamily F, polypeptide 2 (CYP4F2), mRNA [NM_001082]	NM_001082	2.50
A_24_P375573				2.49
A_24_P940680	*KNDC1*	Homo sapiens mRNA for FLJ00252 protein. [AK074179]	AK074179	2.46
A_32_P76627		full-length cDNA clone CS0DI013YN06 of Placenta Cot 25-normalized of Homo sapiens (human). [CR597597]	CR597597	2.46
A_23_P141021	*LPCAT2*	Homo sapiens lysophosphatidylcholine acyltransferase 2 (LPCAT2), mRNA [NM_017839]	NM_017839	2.42
A_32_P213543		AA573434 nk99c02.s1 NCI_CGAP_Co3 Homo sapiens cDNA clone IMAGE:1028930 3', mRNA sequence	AA573434	2.42
A_23_P212830	*FGFR3*	Homo sapiens fibroblast growth factor receptor 3 (FGFR3), transcript variant 1, mRNA [NM_000142]	NM_000142	2.40
A_23_P28772	*DBNDD2*	Homo sapiens dysbindin (dystrobrevin binding protein 1) domain containing 2 (DBNDD2), transcript variant 6, mRNA [NM_001048226]	NM_001048226	2.37
A_24_P366465				2.36
A_24_P33429				2.36
A_24_P401036	*LOC392352*	PREDICTED: Homo sapiens similar to TRIMCyp (LOC392352), mRNA [XM_002342942]	XM_002342942	2.35
A_24_P307384				2.35
A_24_P943263	*RASA4*	Homo sapiens RAS p21 protein activator 4 (RASA4), transcript variant 1, mRNA [NM_006989]	NM_006989	2.34
A_24_P329870	*RHBDF2*	Homo sapiens rhomboid 5 homolog 2 (Drosophila) (RHBDF2), transcript variant 1, mRNA [NM_024599]	NM_024599	2.33
A_23_P94819	*RPH3AL*	Homo sapiens rabphilin 3A-like (without C2 domains) (RPH3AL), mRNA [NM_006987]	NM_006987	2.33
A_32_P156062				2.32
A_23_P86079	*hCG_20426*	Homo sapiens hypothetical protein LOC441869 (LOC441869), transcript variant 1, mRNA [NM_001145210]	NM_001145210	2.31
A_23_P66732	*GSG2*	Homo sapiens germ cell associated 2 (haspin) (GSG2), mRNA [NM_031965]	NM_031965	2.31
A_23_P254688	*TMEM108*	Homo sapiens transmembrane protein 108 (TMEM108), transcript variant 1, mRNA [NM_023943]	NM_023943	2.31
A_32_P84242	*FAM169A*	Homo sapiens family with sequence similarity 169, member A (FAM169A), mRNA [NM_015566]	NM_015566	2.30
A_24_P384059		Homo sapiens similar to Heterogeneous nuclear ribonucleoprotein A1 (Helix-destabilizing protein) (Single-strand binding protein) (hnRNP core protein A1) (HDP-1) (Topoisomerase-inhibitor suppressed), mRNA (cDNA clone IMAGE:2900557), containing frame-shift errors. [BC010266]	BC010266	2.30
A_23_P361679		Homo sapiens mRNA similar to hypothetical protein FLJ21463 (cDNA clone IMAGE:3504595). [BC008001]	BC008001	2.29
A_24_P304154	*AMPD3*	Homo sapiens adenosine monophosphate deaminase 3 (AMPD3), transcript variant 3, mRNA	NM_001025390	2.29
A_24_P418536				2.27
A_24_P144254				2.26
A_24_P204238	*tcag7.1239*	PREDICTED: Homo sapiens similar to Huntingtin interacting protein K (LOC643438), miscRNA [XR_041758]	XR_041758	2.25
A_23_P335486	*C19orf51*	Homo sapiens chromosome 19 open reading frame 51 (C19orf51), mRNA [NM_178837]	NM_178837	2.25
A_32_P226768				2.25
A_23_P81507	*FAT2*	Homo sapiens FAT tumor suppressor homolog 2 (Drosophila) (FAT2), mRNA [NM_001447]	NM_001447	2.25
A_24_P194881	*SHANK3*	Homo sapiens SH3 and multiple ankyrin repeat domains 3 (SHANK3), mRNA [NM_001080420]	NM_001080420	2.24
A_24_P388860	*OSMR*	Homo sapiens oncostatin M receptor (OSMR), transcript variant 1, mRNA [NM_003999]	NM_003999	2.24
A_23_P250358	*HERC6*	Homo sapiens hect domain and RLD 6 (HERC6), transcript variant 1, mRNA [NM_017912]	NM_017912	2.23
A_24_P560332				2.23
A_23_P76823	*ADSSL1*	Homo sapiens adenylosuccinate synthase like 1 (ADSSL1), transcript variant 1, mRNA [NM_199165]	NM_199165	2.22
A_24_P332651	*LOC650157*	PREDICTED: Homo sapiens similar to TRIM5/CypA fusion protein (LOC650157), mRNA [XM_001726994]	XM_001726994	2.22
A_23_P423074	*FAM169A*	Homo sapiens family with sequence similarity 169, member A (FAM169A), mRNA [NM_015566]	NM_015566	2.22
A_23_P333022	*PXT1*	Homo sapiens peroxisomal, testis specific 1 (PXT1), mRNA [NM_152990]	NM_152990	2.22
A_23_P56703	*C2orf89*	Homo sapiens chromosome 2 open reading frame 89 (C2orf89), mRNA [NM_001080824]	NM_001080824	2.20
A_24_P152325				2.19
A_23_P255896	*C2orf89*	Homo sapiens chromosome 2 open reading frame 89 (C2orf89), mRNA [NM_001080824]	NM_001080824	2.19
A_24_P409521				2.19
A_23_P154526	*GRB14*	Homo sapiens growth factor receptor-bound protein 14 (GRB14), mRNA [NM_004490]	NM_004490	2.18
A_23_P429581	*TMEM67*	Homo sapiens transmembrane protein 67 (TMEM67), transcript variant 1, mRNA [NM_153704]	NM_153704	2.18
A_24_P461497	*LOC646048*	PREDICTED: Homo sapiens similar to cytoskeletal beta actin (LOC646048), mRNA [XM_002343637]	XM_002343637	2.18
A_23_P34325	*LRP8*	Homo sapiens low density lipoprotein receptor-related protein 8, apolipoprotein e receptor (LRP8), transcript variant 2, mRNA [NM_033300]	NM_033300	2.17
A_24_P934135		Sequence 1258 from Patent EP1308459. [AX747733]	AX747733	2.17
A_24_P278684	*DFFB*	Homo sapiens DNA fragmentation factor, 40kDa, beta polypeptide (caspase-activated DNase) (DFFB), mRNA [NM_004402]	NM_004402	2.17
A_23_P152428	*MARVELD3*	Homo sapiens MARVEL domain containing 3 (MARVELD3), transcript variant 2, mRNA [NM_052858]	NM_052858	2.17
A_32_P105083	*LOC348751*	Homo sapiens hypothetical protein LOC348751, mRNA (cDNA clone IMAGE:5311172). [BC039445]	BC039445	2.16

A_24_P230388				2.16
A_24_P534290				2.16
A_24_P918762		ALU1_HUMAN (P39188) Alu subfamily J sequence contamination warning entry, partial (7%) [THC2609092]		2.14
A_32_P224234	*LOC645195*	Homo sapiens cDNA FLJ41456 fis, clone BRSTN2012320. [AK123450]	AK123450	2.14
A_32_P7611				2.13
A_23_P123522	*FAM86C*	Homo sapiens family with sequence similarity 86, member C (FAM86C), transcript variant 1, mRNA	NM_018172	2.13
A_24_P308229	*AIM1L*	Homo sapiens absent in melanoma 1-like (AIM1L), mRNA [NM_001039775]	NM_001039775	2.12
A_24_P402784	*SLC38A3*	Homo sapiens solute carrier family 38, member 3 (SLC38A3), mRNA [NM_006841]	NM_006841	2.12
A_23_P83098	*ALDH1A1*	Homo sapiens aldehyde dehydrogenase 1 family, member A1 (ALDH1A1), mRNA [NM_000689]	NM_000689	2.12
A_24_P400751				2.12
A_23_P43296	*FBXL6*	Homo sapiens F-box and leucine-rich repeat protein 6 (FBXL6), transcript variant 1, mRNA [NM_012162]	NM_012162	2.11
A_32_P198584				2.11
A_23_P170246	*OCRL*	Homo sapiens oculocerebrorenal syndrome of Lowe (OCRL), transcript variant a, mRNA [NM_000276]	NM_000276	2.11
A_24_P316586	*DUOX1*	Homo sapiens mRNA; cDNA DKFZp434L0610 (from clone DKFZp434L0610); partial cds. [AL137592]	AL137592	2.10
A_24_P255763				2.09
A_24_P375870				2.07
A_24_P780353				2.07
A_24_P246573	*RP1-21O18.1*	Homo sapiens kazrin (KAZ), transcript variant A, mRNA [NM_015209]	NM_015209	2.06
A_24_P392947				2.05
A_23_P116286	*AMPD3*	Homo sapiens adenosine monophosphate deaminase 3 (AMPD3), transcript variant 3, mRNA	NM_001025390	2.05
A_23_P213359	*PCDH1*	Homo sapiens protocadherin 1 (PCDH1), transcript variant 1, mRNA [NM_002587]	NM_002587	2.05
A_24_P315474	*LOC646973*	PREDICTED: Homo sapiens similar to eukaryotic translation elongation factor 1 beta 2 (LOC646973), miscRNA [XR_038411]	XR_038411	2.04
A_23_P63660	*C10orf58*	Homo sapiens chromosome 10 open reading frame 58 (C10orf58), transcript variant 1, mRNA [NM_032333]	NM_032333	2.04
A_24_P924484		Human melanoma-associated antigen p97 (melanotransferrin) mRNA, 3' flank. [K03200]	K03200	2.03
A_32_P141948		HUMKEREP keratin {Homo sapiens} (exp=-1; wgp=0; cg=0), partial (20%) [THC2564562]	AA595946	2.03
A_24_P187355				2.03
A_24_P82106	*MMP14*	Homo sapiens matrix metallopeptidase 14 (membrane-inserted) (MMP14), mRNA [NM_004995]	NM_004995	2.02
A_24_P170283				2.01
A_24_P320597	*PEX3*	Homo sapiens cDNA FLJ13531 fis, clone PLACE1006288, highly similar to VOLTAGE-DEPENDENT ANION-SELECTIVE CHANNEL PROTEIN 1. [AK023593]	AK023593	2.01
A_24_P16678		Uncharacterized protein C8orf34 (Protein VEST-1) [Source:UniProtKB/Swiss-Prot;Acc:Q49A92]	AK094191	2.01
A_24_P41309	*HNRPA1P5*	full-length cDNA clone CS0DN005YM24 of Adult brain of Homo sapiens (human). [CR623706]	CR623706	2.00
A_24_P475115				2.00
A_24_P143653				-2.01
A_23_P503127	*3-Sep*	Homo sapiens septin 3 (SEPT3), transcript variant A, mRNA [NM_145733]	NM_145733	-2.01
A_24_P339560	*SIGLEC11*	Homo sapiens sialic acid binding Ig-like lectin 11 (SIGLEC11), transcript variant 1, mRNA [NM_052884]	NM_052884	-2.01
A_32_P102300		Q333X5_SPAJD (Q333X5) Heparanase, partial (3%) [THC2687538]		-2.02
A_23_P79769	*BIRC7*	Homo sapiens baculoviral IAP repeat-containing 7 (BIRC7), transcript variant 2, mRNA [NM_022161]	NM_022161	-2.03
A_23_P121695	*CXCL13*	Homo sapiens chemokine (C-X-C motif) ligand 13 (CXCL13), mRNA [NM_006419]	NM_006419	-2.04
A_23_P431430		Putative uncharacterized protein C20orf187 [Source:UniProtKB/Swiss-Prot;Acc:Q9UGB4]	BX281604	-2.04
A_32_P37341	*LOC158863*	Homo sapiens mRNA; cDNA DKFZp586J1922 (from clone DKFZp586J1922) [AL110203]	AL110203	-2.04
A_23_P432626	*SLC25A40*	Homo sapiens solute carrier family 25, member 40 (SLC25A40), nuclear gene encoding mitochondrial protein, mRNA [NM_018843]	NM_018843	-2.05
A_23_P369210	*CLCN5*	H.sapiens voltage-gated chloride ion channel CLCN5. [X91906]	X91906	-2.05
A_23_P153524	*C19orf73*	Homo sapiens chromosome 19 open reading frame 73 (C19orf73), mRNA [NM_018111]	NM_018111	-2.05
A_32_P100109	*REPS2*	Homo sapiens RALBP1 associated Eps domain containing 2 (REPS2), transcript variant 1, mRNA	NM_004726	-2.06
A_24_P299308	*UNQ1887*	Homo sapiens cDNA FLJ39830 fis, clone SPLEN2012846. [AK097149]	AK097149	-2.07
A_32_P164168		BM508017 ij38a01.x1 Human insulinoma Homo sapiens cDNA clone IMAGE:5633160 3', mRNA sequence	BM508017	-2.07
A_24_P607645				-2.07
A_23_P256107	*HPSE*	Homo sapiens heparanase (HPSE), transcript variant 1, mRNA [NM_006665]	NM_006665	-2.09
A_32_P233405		Sequence 1563 from Patent EP1308459. [AX748038]	AX748038	-2.09
A_23_P142506	*GADD45B*	Homo sapiens growth arrest and DNA-damage-inducible, beta (GADD45B), mRNA [NM_015675]	NM_015675	-2.09
A_23_P400580	*FNIP2*	Homo sapiens folliculin interacting protein 2 (FNIP2), mRNA [NM_020840]	NM_020840	-2.09
A_32_P190737	*FNIP2*	Homo sapiens cDNA FLJ25252 fis, clone STM03814. [AK057981]	AK057981	-2.09
A_32_P89415				-2.09
A_32_P161262				-2.10
A_23_P140821	*PARD6A*	Homo sapiens par-6 partitioning defective 6 homolog alpha (C. elegans) (PARD6A), transcript variant 1, mRNA [NM_016948]	NM_016948	-2.10
A_32_P64919	*DIAPH3*	Homo sapiens diaphanous homolog 3 (Drosophila) (DIAPH3), transcript variant 1, mRNA [NM_001042517]	NM_001042517	-2.10
A_24_P102539	*KRIT1*	Homo sapiens KRIT1, ankyrin repeat containing (KRIT1), transcript variant 5, mRNA [NM_001013406]	NM_001013406	-2.10
A_32_P141546				-2.11
A_32_P108738	*CCDC149*	Homo sapiens cDNA FLJ13410 fis, clone PLACE1001720. [AK023472]	AK023472	-2.11
A_24_P920298	*NAIF1*	Homo sapiens nuclear apoptosis inducing factor 1, mRNA (cDNA clone MGC:32044 IMAGE:4810929), complete cds. [BC021580]	BC021580	-2.11
A_23_P300220	*YPEL4*	Homo sapiens yippee-like 4 (Drosophila) (YPEL4), mRNA [NM_145008]	NM_145008	-2.11
A_23_P76633	*NALCN*	Homo sapiens sodium leak channel, non-selective (NALCN), mRNA [NM_052867]	NM_052867	-2.11
A_23_P302005	*STON1*	Homo sapiens stonin 1 (STON1), mRNA [NM_006873]	NM_006873	-2.12
A_23_P160751	*FCRL2*	Homo sapiens Fc receptor-like 2 (FCRL2), mRNA [NM_030764]	NM_030764	-2.12
A_32_P42705	*LOC100144602*	Homo sapiens hypothetical, mRNA (cDNA clone IMAGE:4429392), partial cds. [BC017721]	BC017721	-2.14
A_32_P46594	*LOC145837*	Homo sapiens hypothetical LOC145837 (LOC145837), non-coding RNA [NR_026979]	NR_026979	-2.15
A_23_P161458	*OLAH*	Homo sapiens oleoyl-ACP hydrolase (OLAH), transcript variant 2, mRNA [NM_001039702]	NM_001039702	-2.15
A_32_P99347	*C9orf110*	Homo sapiens chromosome 9 open reading frame 110 (C9orf110), non-coding RNA [NR_024376]	NR_024376	-2.15
A_23_P153301	*CEACAM5*	Homo sapiens carcinoembryonic antigen-related cell adhesion molecule 5 (CEACAM5), mRNA [NM_004363]	NM_004363	-2.15
A_24_P300394	*GSTA2*	Homo sapiens glutathione S-transferase alpha 2 (GSTA2), mRNA [NM_000846]	NM_000846	-2.16
A_32_P78395				-2.17
A_23_P207221	*SLC47A1*	Homo sapiens solute carrier family 47, member 1 (SLC47A1), mRNA [NM_018242]	NM_018242	-2.17
A_24_P345866	*HPSE*	Homo sapiens, clone IMAGE:4690605, mRNA. [BC029464]	BC029464	-2.17
A_32_P188135		ALU1_HUMAN (P39188) Alu subfamily J sequence contamination warning entry, partial (13%) [THC2515921]		-2.17
A_32_P321223	*PMCH*	Homo sapiens pro-melanin-concentrating hormone (PMCH), mRNA [NM_002674]	NM_002674	-2.18
A_32_P155826	*LOC158572*	Homo sapiens hypothetical LOC158572 (LOC158572), non-coding RNA [NR_026742]	NR_026742	-2.19
A_24_P578571	*GFRAL*	Homo sapiens GDNF family receptor alpha like (GFRAL), mRNA [NM_207410]	NM_207410	-2.19
A_23_P428129	*CDKN1C*	Homo sapiens cyclin-dependent kinase inhibitor 1C (p57, Kip2) (CDKN1C), transcript variant 1, mRNA	NM_000076	-2.20
A_32_P59549	*GFRA1*	Homo sapiens GDNF family receptor alpha 1 (GFRA1), transcript variant 2, mRNA [NM_145793]	NM_145793	-2.21
A_32_P14295	*C14orf162*	Homo sapiens chromosome 14 open reading frame 162 (C14orf162), non-coding RNA [NR_024630]	NR_024630	-2.21
A_32_P190571	*RNF207*	Homo sapiens ring finger protein 207 (RNF207), mRNA [NM_207396]	NM_207396	-2.22
A_23_P126363	*ADAM30*	Homo sapiens ADAM metallopeptidase domain 30 (ADAM30), mRNA [NM_021794]	NM_021794	-2.23
A_32_P46351				-2.24
A_32_P48279		BX117479 NCI_CGAP_Lu19 Homo sapiens cDNA clone IMAGp998I075985, mRNA sequence [BX117479]	BX117479	-2.24
A_23_P200512	*ZNF238*	Homo sapiens zinc finger protein 238 (ZNF238), transcript variant 2, mRNA [NM_006352]	NM_006352	-2.24
A_32_P235358		Homo sapiens cDNA FLJ42179 fis, clone THYMU2030796. [AK124173]	AK124173	-2.25
A_24_P92683		immunoglobulin heavy variable 3-38 (non-functional) [Source:HGNC Symbol;Acc:5601] [ENST00000390618]	AK128476	-2.26
A_32_P142586		BQ323779 CM1-CI0092-271000-499-f04 CI0092 Homo sapiens cDNA, mRNA sequence [BQ323779]	BQ323779	-2.26
A_24_P19677	*IL28RA*	Homo sapiens interleukin 28 receptor, alpha (interferon, lambda receptor) (IL28RA), transcript variant 3, mRNA [NM_173065]	NM_173065	-2.26
A_24_P275199	*KCNC1*	Homo sapiens potassium voltage-gated channel, Shaw-related subfamily, member 1 (KCNC1), transcript variant A, mRNA [NM_001112741]	NM_001112741	-2.26
A_24_P179225	*MATN2*	Homo sapiens matrilin 2 (MATN2), transcript variant 2, mRNA [NM_030583]	NM_030583	-2.27
A_23_P81760	*QKI*	Homo sapiens quaking homolog, KH domain RNA binding (mouse) (QKI), transcript variant 1, mRNA	NM_006775	-2.27
A_24_P938015		Homo sapiens mRNA for Zinc-finger protein, partial cds. [D70834]	D70834	-2.28
A_24_P802145	*IRS1*	Homo sapiens insulin receptor substrate 1 (IRS1), mRNA [NM_005544]	NM_005544	-2.28
A_24_P548811		Homo sapiens cDNA FLJ11396 fis, clone HEMBA1000604. [AK021458]	AK021458	-2.29
A_23_P61466	*CD163L1*	Homo sapiens CD163 molecule-like 1 (CD163L1), mRNA [NM_174941]	NM_174941	-2.30

A_32_P140220				-2.30
A_23_P143334	*MACROD2*	Homo sapiens cDNA clone IMAGE:40022102. [BC128036]	BC128036	-2.30
A_23_P113056				-2.30
A_23_P419254	*DIAPH3*	Homo sapiens diaphanous homolog 3 (Drosophila) (DIAPH3), transcript variant 2, mRNA [NM_030932]	NM_030932	-2.31
A_24_P201360	*ACSL5*	Homo sapiens acyl-CoA synthetase long-chain family member 5 (ACSL5), transcript variant 3, mRNA	NM_203380	-2.32
A_24_P362805	*GK5*	Homo sapiens glycerol kinase 5 (putative) (GK5), transcript variant 2, non-coding RNA [NR_033289]	NR_033289	-2.33
A_24_P239606	*GADD45B*	Homo sapiens growth arrest and DNA-damage-inducible, beta (GADD45B), mRNA [NM_015675]	NM_015675	-2.33
A_23_P207003	*4-Sep*	Homo sapiens septin 4 (SEPT4), transcript variant 1, mRNA [NM_004574]	NM_004574	-2.34
A_23_P156087	*GHR*	Homo sapiens growth hormone receptor (GHR), mRNA [NM_000163]	NM_000163	-2.36
A_24_P760960	*SKINTL*	Homo sapiens Skint-like (pseudogene) (SKINTL), non-coding RNA [NR_026749]	NR_026749	-2.37
A_23_P202219	*CALHM2*	Homo sapiens calcium homeostasis modulator 2 (CALHM2), transcript variant 1, mRNA [NM_015916]	NM_015916	-2.38
A_23_P74778	*C1orf54*	Homo sapiens chromosome 1 open reading frame 54 (C1orf54), mRNA [NM_024579]	NM_024579	-2.38
A_23_P102731	*SMOX*	Homo sapiens spermine oxidase (SMOX), transcript variant 1, mRNA [NM_175839]	NM_175839	-2.39
A_24_P321525	*RERG*	Homo sapiens RAS-like, estrogen-regulated, growth inhibitor (RERG), mRNA [NM_032918]	NM_032918	-2.40
A_24_P342829	*SLC16A14*	Homo sapiens solute carrier family 16, member 14 (monocarboxylic acid transporter 14) (SLC16A14), mRNA [NM_152527]	NM_152527	-2.41
A_23_P356004	*KCNIP3*	Homo sapiens Kv channel interacting protein 3, calsenilin (KCNIP3), transcript variant 1, mRNA [NM_013434]	NM_013434	-2.41
A_23_P19482	*DDAH2*	Homo sapiens dimethylarginine dimethylaminohydrolase 2 (DDAH2), mRNA [NM_013974]	NM_013974	-2.41
A_24_P60403	*MGC2848*	Homo sapiens hypothetical protein MGC2848, mRNA (cDNA clone IMAGE:2967248), partial cds. [BC007984]	BC007984	-2.42
A_23_P135164	*UAP1L1*	Homo sapiens UDP-N-acteylglucosamine pyrophosphorylase 1-like 1 (UAP1L1), mRNA [NM_207309]	NM_207309	-2.44
A_23_P354609		Homo sapiens cDNA FLJ38264 fis, clone FCBBF3001657. [AK095583]	AK095583	-2.44
A_23_P251387	*REPS2*	Homo sapiens RALBP1 associated Eps domain containing 2 (REPS2), transcript variant 1, mRNA	NM_004726	-2.45
A_23_P417974	*AQP11*	Homo sapiens aquaporin 11 (AQP11), mRNA [NM_173039]	NM_173039	-2.46
A_24_P579356	*ARHGAP28*	Homo sapiens Rho GTPase activating protein 28 (ARHGAP28), transcript variant 1, mRNA [NM_001010000]	NM_001010000	-2.46
A_32_P65589		Homo sapiens partial mRNA for immunoglobulin anti-gp96/grp94 variable region of heavy chain (DP-3 derivative gene), clone H11B. [AJ252276]	AJ252276	-2.46
A_23_P348876		Homo sapiens cDNA FLJ12616 fis, clone NT2RM4001650. [AK022678]	AK022678	-2.49
A_23_P151710	*PTGER2*	Homo sapiens prostaglandin E receptor 2 (subtype EP2), 53kDa (PTGER2), mRNA [NM_000956]	NM_000956	-2.49
A_23_P380298	*ProSAPiP1*	Homo sapiens ProSAPiP1 protein (ProSAPiP1), mRNA [NM_014731]	NM_014731	-2.51
A_32_P64004		NM_067624 Y55F3AM.3a {Caenorhabditis elegans} (exp=-1; wgp=0; cg=0), partial (4%) [THC2539939]		-2.53
A_24_P109887		LOC554207 protein [Source:UniProtKB/TrEMBL;Acc:Q8N1B8] [ENST00000320322]	BC031469	-2.55
A_23_P429977	*KCNQ1*	Homo sapiens potassium voltage-gated channel, KQT-like subfamily, member 1 (KCNQ1), transcript variant 1, mRNA [NM_000218]	NM_000218	-2.56
A_24_P592570		Q5CVE8_CRYPV (Q5CVE8) Fibrillarin. RNA methylase, partial (6%) [THC2507877]		-2.58
A_24_P185689	*ACSS1*	Homo sapiens acyl-CoA synthetase short-chain family member 1 (ACSS1), nuclear gene encoding mitochondrial protein, mRNA [NM_032501]	NM_032501	-2.62
A_32_P148146		BF955364 RC5-NN0245-171100-021-G10 NN0245 Homo sapiens cDNA, mRNA sequence [BF955364]	BF955364	-2.66
A_24_P185117	*RILP*	Homo sapiens Rab interacting lysosomal protein (RILP), mRNA [NM_031430]	NM_031430	-2.67
A_24_P315256				-2.68
A_23_P53176	*FOLR1*	Homo sapiens folate receptor 1 (adult) (FOLR1), transcript variant 1, mRNA [NM_016725]	NM_016725	-2.70
A_23_P6099	*PLCB1*	Homo sapiens phospholipase C, beta 1 (phosphoinositide-specific) (PLCB1), transcript variant 1, mRNA	NM_015192	-2.72
A_23_P26815	*RILP*	Homo sapiens Rab interacting lysosomal protein (RILP), mRNA [NM_031430]	NM_031430	-2.73
A_32_P83811	*FAM47E*	Homo sapiens family with sequence similarity 47, member E (FAM47E), mRNA [NM_001136570]	NM_001136570	-2.74
A_23_P397320	*JSRP1*	Homo sapiens junctional sarcoplasmic reticulum protein 1 (JSRP1), mRNA [NM_144616]	NM_144616	-2.75
A_23_P85008	*MAOB*	Homo sapiens monoamine oxidase B (MAOB), nuclear gene encoding mitochondrial protein, mRNA	NM_000898	-2.81
A_24_P323941	*C20orf106*	Homo sapiens chromosome 20 open reading frame 106 (C20orf106), mRNA [NM_001012971]	NM_001012971	-2.87
A_32_P58029				-2.88
A_23_P258582	*GK5*	Homo sapiens glycerol kinase 5 (putative) (GK5), transcript variant 1, mRNA [NM_001039547]	NM_001039547	-2.89
A_23_P37785	*KCTD19*	Homo sapiens cDNA FLJ52078 complete cds, highly similar to Mus musculus potassium channel tetramerisation domain containing 19 (Kctd19), mRNA. [AK302567]	AK302567	-2.91
A_23_P48988	*SH3GL3*	Homo sapiens SH3-domain GRB2-like 3 (SH3GL3), transcript variant 1, mRNA [NM_003027]	NM_003027	-2.92
A_23_P148015	*AXIN2*	Homo sapiens axin 2 (AXIN2), mRNA [NM_004655]	NM_004655	-2.92
A_23_P304373	*HTR5A*	Homo sapiens 5-hydroxytryptamine (serotonin) receptor 5A (HTR5A), mRNA [NM_024012]	NM_024012	-2.92
A_23_P113748	*ZNF385D*	Homo sapiens zinc finger protein 385D (ZNF385D), mRNA [NM_024697]	NM_024697	-2.94
A_32_P17484		Homo sapiens mRNA; cDNA DKFZp686J0581 (from clone DKFZp686J0581). [BX647685]	BX647685	-3.02
A_23_P200976	*HYI*	Homo sapiens hydroxypyruvate isomerase homolog (E. coli) (HYI), transcript variant 1, mRNA [NM_031207]	NM_031207	-3.03
A_32_P25419		Synthetic construct Homo sapiens gateway clone IMAGE:100021719 3' read SEPP1 mRNA. [CU691335]	CU691335	-3.05
A_24_P319923	*MYLK*	Homo sapiens myosin light chain kinase (MYLK), transcript variant 1, mRNA [NM_053025]	NM_053025	-3.06
A_24_P558135		tx28f05.x1 NCI_CGAP_Lu24 Homo sapiens cDNA clone IMAGE:2270913 3', mRNA sequence [AI801879]	AI801879	-3.07
A_23_P160582	*HYI*	Homo sapiens hydroxypyruvate isomerase homolog (E. coli) (HYI), transcript variant 1, mRNA [NM_031207]	NM_031207	-3.08
A_23_P338134	*KIAA1920*	Homo sapiens mRNA for KIAA1920 protein, partial cds. [AB067507]	AB067507	-3.10
A_32_P117232		AW450111 UI-H-BI3-akw-e-08-0-UI.s1 NCI_CGAP_Sub5 Homo sapiens cDNA clone IMAGE:2735726 3', mRNA sequence [AW450111]	AW450111	-3.11
A_23_P128503	*IL26*	Homo sapiens interleukin 26 (IL26), mRNA [NM_018402]	NM_018402	-3.12
A_24_P364591	*FBLN5*	Homo sapiens fibulin 5 (FBLN5), mRNA [NM_006329]	NM_006329	-3.22
A_32_P181297	*ST7OT1*	Homo sapiens ST7 overlapping transcript 1 (non-protein coding) (ST7OT1), antisense RNA [NR_002330]	NR_002330	-3.24
A_23_P210425	*MYL9*	Homo sapiens myosin, light chain 9, regulatory (MYL9), transcript variant 2, mRNA [NM_181526]	NM_181526	-3.26
A_23_P5131	*ISYNA1*	Homo sapiens inositol-3-phosphate synthase 1 (ISYNA1), transcript variant 1, mRNA [NM_016368]	NM_016368	-3.27
A_24_P418744				-3.27
A_24_P71904	*HPGD*	Homo sapiens hydroxyprostaglandin dehydrogenase 15-(NAD) (HPGD), transcript variant 1, mRNA	NM_000860	-3.28
A_32_P7176		AW949170 QV4-FT0006-110500-206-c01 FT0006 Homo sapiens cDNA, mRNA sequence [AW949170]	AW949170	-3.28
A_23_P104522	*NEBL*	Homo sapiens nebulette (NEBL), transcript variant 1, mRNA [NM_006393]	NM_006393	-3.31
A_32_P207169	*C1orf133*	Homo sapiens chromosome 1 open reading frame 133 (C1orf133), non-coding RNA [NR_024337]	NR_024337	-3.32
A_23_P145935	*EPHB6*	Homo sapiens EPH receptor B6 (EPHB6), mRNA [NM_004445]	NM_004445	-3.35
A_23_P128728	*ARG2*	Homo sapiens arginase, type II (ARG2), nuclear gene encoding mitochondrial protein, mRNA [NM_001172]	NM_001172	-3.36
A_32_P118675		Q3AUE7_CHLCH (Q3AUE7) Glucosamine-fructose-6-phosphate aminotransferase, isomerising , partial (4%) [THC2660784]		-3.36
A_23_P93141	*GSTA5*	Homo sapiens glutathione S-transferase alpha 5 (GSTA5), mRNA [NM_153699]	NM_153699	-3.45
A_32_P183718	*SP5*	Homo sapiens Sp5 transcription factor (SP5), mRNA [NM_001003845]	NM_001003845	-3.51
A_23_P143817	*MYLK*	Homo sapiens myosin light chain kinase (MYLK), transcript variant 1, mRNA [NM_053025]	NM_053025	-3.62
A_23_P131394	*SLC16A14*	Homo sapiens solute carrier family 16, member 14 (monocarboxylic acid transporter 14) (SLC16A14), mRNA [NM_152527]	NM_152527	-3.64
A_24_P104407	*SYNM*	Homo sapiens synemin, intermediate filament protein (SYNM), transcript variant A, mRNA [NM_145728]	NM_145728	-3.66
A_23_P213050	*HPGD*	Homo sapiens hydroxyprostaglandin dehydrogenase 15-(NAD) (HPGD), transcript variant 1, mRNA	NM_000860	-3.72
A_23_P128235	*KRT1*	Homo sapiens keratin 1 (KRT1), mRNA [NM_006121]	NM_006121	-3.75
A_23_P118203	*ZG16B*	Homo sapiens zymogen granule protein 16 homolog B (rat) (ZG16B), mRNA [NM_145252]	NM_145252	-3.78
A_24_P925505	*CD36*	CD36=collagen type I/thrombospondin receptor {one exon} [human, mRNA Partial, 369 nt]. [S67044]	S67044	-3.82
A_23_P140748	*NDRG4*	Homo sapiens NDRG family member 4 (NDRG4), transcript variant 3, mRNA [NM_022910]	NM_022910	-3.85
A_23_P83134	*GAS1*	Homo sapiens growth arrest-specific 1 (GAS1), mRNA [NM_002048]	NM_002048	-3.88
A_23_P120594	*ACSS1*	Homo sapiens acyl-CoA synthetase short-chain family member 1 (ACSS1), nuclear gene encoding mitochondrial protein, mRNA [NM_032501]	NM_032501	-3.90
A_23_P212258	*KNG1*	Homo sapiens kininogen 1 (KNG1), transcript variant 2, mRNA [NM_000893]	NM_000893	-3.91
A_23_P111583	*CD36*	Homo sapiens CD36 molecule (thrombospondin receptor) (CD36), transcript variant 2, mRNA	NM_001001547	-3.93
A_32_P218734	*COL5A2*	Homo sapiens collagen, type V, alpha 2 (COL5A2), mRNA [NM_000393]	NM_000393	-4.00
A_32_P146309	*CACNB2*	Homo sapiens cDNA FLJ45229 fis, clone BRCAN2020972. [AK128769]	AK128769	-4.01
A_23_P69030	*COL8A1*	Homo sapiens collagen, type VIII, alpha 1 (COL8A1), transcript variant 1, mRNA [NM_001850]	NM_001850	-4.09
A_24_P171043	*DKFZP547L112*	Homo sapiens mRNA; cDNA DKFZp547L112 (from clone DKFZp547L112). [AL512723]	AL512723	-4.17
A_23_P125505	*PPEF1*	Homo sapiens protein phosphatase, EF-hand calcium binding domain 1 (PPEF1), transcript variant 1, mRNA [NM_006240]	NM_006240	-4.17
A_23_P128362	*MYBPC1*	Homo sapiens myosin binding protein C, slow type (MYBPC1), transcript variant 2, mRNA [NM_206819]	NM_206819	-4.18
A_23_P36901	*CPB2*	Homo sapiens carboxypeptidase B2 (plasma) (CPB2), transcript variant 1, mRNA [NM_001872]	NM_001872	-4.30
A_24_P402825	*CACNA2D3*	Homo sapiens calcium channel, voltage-dependent, alpha 2/delta subunit 3 (CACNA2D3), mRNA	NM_018398	-4.36
A_23_P46879	*PNLIP*	Homo sapiens pancreatic lipase (PNLIP), mRNA [NM_000936]	NM_000936	-4.51

A_23_P169978	*ZNF608*	Homo sapiens zinc finger protein 608 (ZNF608), mRNA [NM_020747]	NM_020747	-4.51
A_23_P135381	*SP5*	Homo sapiens Sp5 transcription factor (SP5), mRNA [NM_001003845]	NM_001003845	-4.52
A_23_P147166	*CACNA2D3*	Homo sapiens mRNA for calcium channel alpha2-delta3 subunit. [AJ272268]	AJ272268	-4.67
A_32_P197561	*EBF1*	Homo sapiens early B-cell factor 1 (EBF1), mRNA [NM_024007]	NM_024007	-4.67
A_23_P422071	*B3GALT4*	Homo sapiens UDP-Gal:betaGlcNAc beta 1,3-galactosyltransferase, polypeptide 4 (B3GALT4), mRNA	NM_003782	-4.71
A_32_P71032				-4.71
A_23_P24129	*DKK1*	Homo sapiens dickkopf homolog 1 (Xenopus laevis) (DKK1), mRNA [NM_012242]	NM_012242	-4.77
A_23_P111171	*B3GALT4*	Homo sapiens UDP-Gal:betaGlcNAc beta 1,3-galactosyltransferase, polypeptide 4 (B3GALT4), mRNA	NM_003782	-4.86
A_32_P36905				-4.93
A_23_P104493	*PAPSS2*	Homo sapiens 3'-phosphoadenosine 5'-phosphosulfate synthase 2 (PAPSS2), transcript variant 2, mRNA [NM_001015880]	NM_001015880	-5.00
A_23_P162171	*MCAM*	Homo sapiens melanoma cell adhesion molecule (MCAM), mRNA [NM_006500]	NM_006500	-5.00
A_24_P10137	*C13orf15*	Homo sapiens chromosome 13 open reading frame 15 (C13orf15), mRNA [NM_014059]	NM_014059	-5.25
A_23_P204937	*C13orf15*	Homo sapiens chromosome 13 open reading frame 15 (C13orf15), mRNA [NM_014059]	NM_014059	-5.25
A_23_P156880	*ENPP1*	Homo sapiens ectonucleotide pyrophosphatase/phosphodiesterase 1 (ENPP1), mRNA [NM_006208]	NM_006208	-5.27
A_23_P349566	*CCDC85A*	Homo sapiens coiled-coil domain containing 85A (CCDC85A), mRNA [NM_001080433]	NM_001080433	-5.32
A_24_P940469		alcohol dehydrogenase 1B (class I), beta polypeptide [Source:HGNC Symbol;Acc:250] [ENST00000305046]	AF153821	-5.53
A_23_P58359	*ADH1A*	Homo sapiens alcohol dehydrogenase 1A (class I), alpha polypeptide (ADH1A), mRNA [NM_000667]	NM_000667	-5.53
A_24_P291658	*ADH1A*	Homo sapiens alcohol dehydrogenase 1A (class I), alpha polypeptide (ADH1A), mRNA [NM_000667]	NM_000667	-5.62
A_23_P56197	*CRLF1*	Homo sapiens cytokine receptor-like factor 1 (CRLF1), mRNA [NM_004750]	NM_004750	-5.95
A_23_P11674	*PTGFR*	Homo sapiens prostaglandin F receptor (FP) (PTGFR), transcript variant 2, mRNA [NM_001039585]	NM_001039585	-5.99
A_23_P40415	*ADAMTS5*	Homo sapiens ADAM metallopeptidase with thrombospondin type 1 motif, 5 (ADAMTS5), mRNA [NM_007038]	NM_007038	-6.27
A_23_P81158	*ADH1C*	Homo sapiens alcohol dehydrogenase 1C (class I), gamma polypeptide (ADH1C), mRNA [NM_000669]	NM_000669	-6.63
A_23_P253896	*NPNT*	Homo sapiens nephronectin (NPNT), mRNA [NM_001033047]	NM_001033047	-6.75
A_24_P940166	*PAPSS2*	Homo sapiens 3'-phosphoadenosine 5'-phosphosulfate synthase 2 (PAPSS2), transcript variant 2, mRNA [NM_001015880]	NM_001015880	-6.88
A_24_P302172	*PTGFR*	Homo sapiens prostaglandin F receptor (FP) (PTGFR), transcript variant 2, mRNA [NM_001039585]	NM_001039585	-7.04
A_23_P151805	*FBLN5*	Homo sapiens fibulin 5 (FBLN5), mRNA [NM_006329]	NM_006329	-7.16
A_24_P139152	*COL8A1*	collagen, type VIII, alpha 1 [Source:HGNC Symbol;Acc:2215] [ENST00000261037]	AL359062	-7.73
A_32_P735429		RNSAP seum amyloid P component {Rattus norvegicus} (exp=-1; wgp=0; cg=0), partial (30%) [THC2654992]		-7.88
A_23_P330611	*WIPF1*	Homo sapiens WAS/WASL interacting protein family, member 1 (WIPF1), transcript variant 2, mRNA	NM_001077269	-8.04
A_23_P34597	*CDA*	Homo sapiens cytidine deaminase (CDA), mRNA [NM_001785]	NM_001785	-8.11
A_23_P43164	*SULF1*	Homo sapiens sulfatase 1 (SULF1), transcript variant 3, mRNA [NM_015170]	NM_015170	-10.08
A_23_P102611	*WISP2*	Homo sapiens WNT1 inducible signaling pathway protein 2 (WISP2), mRNA [NM_003881]	NM_003881	-11.46
A_23_P214208	*CNR1*	Homo sapiens cannabinoid receptor 1 (brain) (CNR1), transcript variant 2, mRNA [NM_033181]	NM_033181	-11.89
A_23_P102000	*CXCR4*	Homo sapiens chemokine (C-X-C motif) receptor 4 (CXCR4), transcript variant 1, mRNA [NM_001008540]	NM_001008540	-12.66
