# Supplementary material for: Loss of the BRCA1-Interacting Helicase BRIP1 Results in Abnormal Mammary Acinar Morphogenesis
Source: PLoS One. 2013 Sep 6;8(9):e74013. doi: 10.1371/journal.pone.0074013 (PMC3765252; doi:10.1371/journal.pone.0074013)
Supplement: Table S4 — Gene Set Enrichment Analysis (GSEA) gene sets positively or negatively correlated with 3D cultured BRIP1-knockdown cells compared with control cells. (PDF) [file pone.0074013.s006.pdf]

Table S4. Gene Set Enrichment Analysis (GSEA) gene sets positively or negatively correlated with 3D cultured *BRIP1* -knockdown cells compared with control cells

ES, GSEA enrichment score; NES, GSEA normalized enrichment score; FDR, false discovery rate. Only gene sets that show *P* < 0.05 and FDR *q* < 0.25 are listed.

No.	Database	Name of Gene Set	Size (number of genes)	ES	NES	Nominal *P* -value	FDR *q* -value
**Gene sets positively correlated with *BRIP* 1-knockdown cells compared with control cells**							
1	Curated (C2)	UVB_NHEK4_24HRS_DN	16	0.745	2.019	0.002	0.012
2	Curated (C2)	HINATA_NFKB_DN	19	0.742	1.844	0.000	0.113
3	Curated (C2)	TSA_CD4_UP	25	0.601	1.792	0.004	0.166
4	Curated (C2)	CROMER_HYPOPHARYNGEAL_MET_VS_NON_DN	77	0.579	1.750	0.002	0.215
5	Curated (C2)	VEGF_HUVEC_2HRS_UP	29	0.597	1.742	0.006	0.190
6	Curated (C2)	PTDINSPATHWAY	21	0.622	1.741	0.014	0.161
7	Curated (C2)	ET743_SARCOMA_6HRS_UP	28	0.519	1.732	0.004	0.155
8	Curated (C2)	UVB_NHEK4_6HRS_UP	27	0.655	1.731	0.000	0.139
9	Curated (C2)	HSA03010_RIBOSOME	64	0.486	1.710	0.030	0.159
10	Curated (C2)	UVB_NHEK3_C8	64	0.497	1.697	0.006	0.171
11	Curated (C2)	ATMPATHWAY	20	0.634	1.691	0.002	0.169
12	Curated (C2)	BLEO_HUMAN_LYMPH_HIGH_4HRS_UP	21	0.745	1.678	0.020	0.180
13	Curated (C2)	AGED_MOUSE_RETINA_ANY_UP	20	0.543	1.675	0.012	0.172
14	Curated (C2)	HDACI_COLON_CURSUL_UP	39	0.505	1.661	0.008	0.187
15	Curated (C2)	MMS_HUMAN_LYMPH_HIGH_24HRS_UP	19	0.692	1.653	0.008	0.193
16	Curated (C2)	UVC_HIGH_ALL_UP	15	0.724	1.648	0.015	0.192
17	Curated (C2)	KANNAN_P53_UP	37	0.624	1.645	0.018	0.187
18	Curated (C2)	FERRANDO_T_CELL_DIFFERENTIATION_PATHWAY	16	0.555	1.630	0.035	0.210
19	Curated (C2)	UVB_NHEK4_6HRS_DN	18	0.582	1.623	0.006	0.213
20	Curated (C2)	HSA05131_PATHOGENIC_ESCHERICHIA_COLI_INFECTION_EPE	38	0.515	1.600	0.022	0.249
21	Curated (C2)	ERKPATHWAY	29	0.541	1.598	0.031	0.245
22	Curated (C2)	NI2_MOUSE_UP	40	0.473	1.596	0.016	0.238
**Gene sets negatively correlated with *BRIP1* -knockdown cells compared with control cells**							
23	Curated (C2)	PROSTAGLANDIN_SYNTHESIS_REGULATION	28	-0.625	-1.979	0.000	0.018
24	Curated (C2)	SA_PTEN_PATHWAY	17	-0.651	-1.785	0.002	0.149
25	Curated (C2)	HSA00530_AMINOSUGARS_METABOLISM	26	-0.628	-1.728	0.004	0.197
26	Curated (C2)	STARCH_AND_SUCROSE_METABOLISM	29	-0.551	-1.721	0.008	0.161
27	Curated (C2)	HSA00040_PENTOSE_AND_GLUCURONATE_INTERCONVERSIO	16	-0.575	-1.689	0.014	0.190
28	Curated (C2)	CCR5PATHWAY	17	-0.571	-1.670	0.027	0.200
29	Curated (C2)	ANDROGEN_AND_ESTROGEN_METABOLISM	21	-0.491	-1.655	0.012	0.200
30	Curated (C2)	SMALL_LIGAND_GPCRS	17	-0.629	-1.632	0.028	0.218
31	Curated (C2)	INOSITOL_PHOSPHATE_METABOLISM	21	-0.618	-1.620	0.014	0.217
32	Curated (C2)	GLYCOLYSIS	50	-0.383	-1.585	0.008	0.231
33	Curated (C2)	TYROSINE_METABOLISM	31	-0.526	-1.579	0.027	0.226
