# Supplementary material for: Loss of the BRCA1-Interacting Helicase BRIP1 Results in Abnormal Mammary Acinar Morphogenesis
Source: PLoS One. 2013 Sep 6;8(9):e74013. doi: 10.1371/journal.pone.0074013 (PMC3765252; doi:10.1371/journal.pone.0074013)
Supplement: Table S5 — Oligonucleotide sequences used for quantitative RT-PCR. (PDF) [file pone.0074013.s007.pdf]

Table S5. Oligonucleotide sequences used for quantitative RT-PCR

Gene	Forward (5' to 3')	Reverse (5' to 3')
SATB1	AGGAAATGAAGCGTGCTAAAGTG	AGAGGTTCTCCCACAGGGTTC
RICH2	CTGTGCAGGGACTCAACCAG	AATCGGTGCCAGCTTCTTTG
PCSK5	ATGGGAGATGCGTGCAGAG	CACTAGGGCAGCTTGTACAGTTCTT
ELF3	GAACAGCAACATGACCTACGAGA	TGGAGAACCTCTTCCTCCTTCC
WIPF1	AGCAAACTGGCAAGAAACGAA	GGGTAGAGAAGAGCAGGCAAAG
MCAM	CAGAAGAGATGGGCCTCCTG	CAGGGAAGGGAGCTGAAGTG
COL8A1	TGGCAAAGAGTATCCACACCTACC	TTCCCCTCGTAAACTGGCTAATG
GAS1	GCGAGTCGGTCAAGGAGAA	CGTCATCGTAGTCCTCATCGTAG
