# Supplementary material for: Loss of the BRCA1-Interacting Helicase BRIP1 Results in Abnormal Mammary Acinar Morphogenesis
Source: PLoS One. 2013 Sep 6;8(9):e74013. doi: 10.1371/journal.pone.0074013 (PMC3765252; doi:10.1371/journal.pone.0074013)
Supplement: Methods S1 — DNA microarray data analysis. (DOC) [file pone.0074013.s008.doc]

**Supplementary Methods**

**DNA Microarray Data Analysis**

To visualize the differences in gene expression profiles in the 3D culture of the *BRIP1*-knockdown and control cells, principal component analysis was performed using GeneSpring GX 11.5.1 software (Agilent Technologies) (as shown in Fig. S2A). The differentially expressed genes between the *BRIP1*-knockdown and control cells at each time point in the 3D culture (days 4, 8, and 12) were identified by Student’s *t*-test (unpaired; *P* < 0.05) (as provided in Tables S1–3). Gene Ontology (GO) terms were assigned to each differentially expressed gene (as shown in Fig. S2B). To examine the enrichment of the differentially expressed genes in signaling pathways, the Find Significant Pathway tool in GeneSpring GX 11.5.1 was used (as shown in Fig. 3C). Alternatively, an ANOVA analysis (*P* < 0.05, Benjamini-Hochberg false discovery rate as multiple testing correction) was applied to identify significantly differentially expressed genes during the 3D culture of the *BRIP1*-knockdown and control cells (as shown in Fig. 3A and Table 1). Gene Set Enrichment Analysis (GSEA) was used as a computational method to identify sets of genes with coordinate differences in gene expression (<http://www.broad.mit.edu/gsea/>). Genes were ranked using the provided signal-to-noise ranking statistic, and GSEA was run using a weighted statistic and evaluated for statistical significance by comparison with the results obtained using 1,000 random permutations of each gene set. For all other GSEA parameters, default settings were used. Gene sets analyzed were obtained from the Molecular Signatures Database (MSigDB C2 collection, v2.5, <http://www.broad.mit.edu/gsea/msigdb/index.jsp>).
